# Embeddings from language models are good learners for single-cell data analysis

**DOI:** 10.1016/j.patter.2025.101431

**Published:** 2026-01-30

**Authors:** Tianyu Liu, Tianqi Chen, Wangjie Zheng, Xiao Luo, Yiqun Chen, Hongyu Zhao

**Affiliations:** 1 Interdepartmental Program in Computational Biology & Bioinformatics, Yale University, New Haven, CT 06511, USA; 2 Department of Biostatistics, Yale University, New Haven, CT 06511, USA; 3 Department of Statistics, University of Wisconsin-Madison, Madison, WI 53706, USA; 4 Department of Biostatistics, Johns Hopkins University, Baltimore, MD 21218, USA

**Keywords:** single-cell data analysis, foundation model, large language model, *in-silico* treatment analysis, perturbation analysis

## Abstract

Foundation models (FMs) have been built to analyze single-cell data with different degrees of success. Here, we present scELMo (single-cell embedding from language models), a method for analyzing single-cell data with the help of large language models (LLMs). LLMs can generate both the description of metadata information and the embeddings for such descriptions. We then combine the embeddings from LLMs with the raw data under the zero-shot learning framework to further extend its function by using the fine-tuning framework to handle different tasks. We demonstrate that scELMo is capable of cell clustering, batch effect correction, and cell-type annotation without training a new model. Moreover, the fine-tuning framework of scELMo can help with more challenging tasks, including *in silico* treatment analysis or modeling perturbation. scELMo has a lighter structure and lower requirements for resources, suggesting a more promising path.

## Highlights

• scELMo leverages large language models to integrate knowledge
• scELMo supports multiple settings
• scELMo achieves superior performance with fewer resources
• scELMo establishes a scalable and interpretable framework

## The bigger picture

Single-cell technologies allow scientists to measure the activity of thousands of genes in individual cells, revealing how tissues develop, age, and respond to disease. Yet, analyzing these massive datasets often demands substantial computing resources and specialized expertise. Our method, single-cell embedding from language models (scELMo), offers an accessible solution by harnessing large language models—the same artificial intelligence systems behind modern chatbots—to interpret biological information. Instead of training large models from scratch, scELMo uses pre-trained language models’ knowledge of gene functions and biological concepts to generate detailed numerical representations, or embeddings, of genes. These embeddings capture complex biological information and can be integrated with cellular data to facilitate tasks such as identifying cell types, understanding developmental processes, or exploring disease mechanisms. By uniting advances in computational linguistics and genomics, scELMo transforms language models into engines of biological discovery, expanding access to powerful single-cell analysis tools and accelerating the pace of biomedical insight.

## Teaser

scELMo introduces a way to analyze massive single-cell datasets by harnessing large language models to summarize biological knowledge about each gene. These summaries are transformed into mathematical embeddings that integrate with cell data, enabling efficient cell grouping, batch correction, and treatment prediction. By merging language understanding with biological data, scELMo reduces computational demands and democratizes advanced analysis—offering a faster, more accessible path to discoveries that could inform new therapies and deepen understanding of human health.

## Introduction

The development of foundation models (FMs) has become increasingly critical across a wide range of domains, including engineering and the sciences.^1^^,^^2^^,^^3^ Large language models (LLMs) serve as prominent examples of successful FMs. In the field of biology, FMs have been employed for various applications, such as analyzing DNA sequences^4^^,^^5^ and representing cellular and gene-level information,^6^^,^^7^^,^^8^^,^^9^ among others. These models have been shown to enhance the performance of diverse downstream tasks. In this work, we concentrate on the intersection of FMs and a specific type of biomedical tabular data: single-cell sequencing data.^10^^,^^11^ Single-cell sequencing captures molecular profiles at the resolution of individual cells, enabling detailed characterization of cellular activity and identity. Typical features include gene expression levels,^11^ protein abundance,^12^^,^^13^ and DNA methylation states,^14^ among others.

A number of pre-trained FMs have been developed to analyze single-cell data using large-scale sequencing datasets compiled from diverse studies. Notable examples include scGPT,^7^ Geneformer,^8^ and scFoundation,^15^ which leverage extensive single-cell transcriptomic data to learn biological patterns. These models are subsequently applied to downstream tasks by either utilizing cell or gene embeddings as learned representations or by fine-tuning the pre-trained models on newly generated datasets. However, such training protocols require substantial computational resources and storage capacity, and their performance can be highly sensitive to data pre-processing choices and gene selection strategies. Recent benchmarking studies^16^^,^^17^ have highlighted limitations in the scalability, robustness, and generalizability of current single-cell FMs. In parallel, the developers of GenePT^18^ identified a key constraint in relying solely on gene expression profiles for biological inference. To address this, they proposed augmenting gene representations using prompts derived from external biological knowledge. Specifically, GenePT-w extracts gene embeddings from LLMs by feeding them information from the National Center for Biotechnology Information (NCBI),^19^ while GenePT-s converts single-cell profiles into ranked gene lists and uses them as prompts to generate cell embeddings.

Although these strategies demonstrate potential for incorporating prior knowledge, they have notable limitations. GenePT-w depends on the structure and completeness of NCBI annotations, which may not fully capture gene function or interactions. GenePT-s is constrained by the intrinsic sparsity of single-cell datasets, which leads to many unexpressed genes that cannot be ranked reliably. Additionally, its scalability is limited by reliance on the OpenAI application programming interface (API), which imposes usage and latency restrictions.^20^ GenePT also explores zero-shot transfer learning, but its inability to integrate cell-level metadata, such as cell-type annotations, restricts its applicability to a broader set of tasks. Meanwhile, the emergence of advanced LLMs, such as GPT-3.5,^21^ GPT-4,^22^ and LLaMA,^23^ provides opportunities to integrate large-scale biological knowledge into single-cell analysis. These models have already been applied to scientific knowledge extraction,^24^ neural architecture design,^25^ and broader applications in computational research.^26^^,^^27^

In this manuscript, we explore the ability of using LLMs in a different manner. We generate meaningful text descriptions of cell-level or feature-level metadata as well as embeddings of such descriptions based on LLMs. We assume that the embeddings from LLMs carry biological properties and can be utilized in various downstream applications. Here, we introduce single-cell embedding from language models (scELMo) as a pipeline for analyzing single-cell multi-omics data based on the text description and embeddings directly from LLMs.^28^ Using genes as one example, we leverage LLMs to summarize the functional information of a given gene with a suitable prompt and also use the same LLM to extract the embeddings of such a description. We then either incorporate the embeddings directly into the single-cell data by matrix operation or combine the embeddings with other models with fine-tuning targets for various tasks. Different from traditional single-cell FMs, scELMo does not require pre-training with LLM embeddings, and it saves resources to accelerate biological discoveries and insight validation. We demonstrate that scELMo is a simple but effective tool for single-cell data analysis under both the zero-shot learning framework and the fine-tuning framework, supported by comprehensive benchmarking analyses.

## Methods

### Problem definition

For a typical single-cell dataset *X*^*n*×*m*^ after normalization^29^ with *n* cells and *m* features, our target is to utilize the text description from a mapping function M for feature-level metadata information *f*^*m*×1^ and cell-level metadata information *c*^*n*×1^ to learn the embeddings of cells. Each cell or gene has one corresponding metadata information description, and thus, *f* and *g* are vectors. If we define the embedding generation layer of M as $M_{e}$, our cell embeddings ($e_{cells}^{n\times t}$, where *t* represents the dimension of LLM embeddings) can be represented as

$$\begin{array}{l} e_{f}^{m\times t}=M_{e}\left(M\left(Prompt\left(f\right)\right)\right)\text{,}\\ e_{c}^{n\times t}=M_{e}\left(M\left(Prompt\left(c\right)\right)\right)\text{,}\\ e_{cells}^{n\times t}=AVG\left(X\right)e_{f}+e_{c}\text{,} \end{array}\;\text{and}$$

where *Prompt* is a mapping function that can transfer the name of input data to the prompt space. The prompts can be used as the input for language models. The function *AVG*() represents the method we used to average the embeddings of all genes for each cell. If the mode is the arithmetic average (*aa*), we divide *X* by *m*. If the mode is the *wa*, we divide each row of *X* by the sum of this row. Considering the cell with index *i*, we can define these two processes as

$$\begin{array}{l} AVG_{aa}\left(X_{i}\right)=\frac{X_{i}}{m}\;\text{and}\\ AVG_{wa}\left(X_{i}\right)=\frac{X_{i}}{sum\left(X_{i}\right)}\text{.} \end{array}$$

Then, we use matrix multiplication to combine the feature embeddings and the expression profile. Our default setting of the mapping function is an LLM. GenePT can be treated as a special case of scELMo, that is, replacing the LLM with a known database and using the *aa* mode. Incorporating the embeddings of cell-level metadata is an optional choice. We intend to investigate if the cell embeddings can offer a better representation than the raw data.

Moreover, with the embeddings of feature-level metadata information and cell-level metadata information, we also consider if incorporating our embeddings with task-specific model T can improve the performance of T, that is

$$Score\left(T\left(X\right)\right)<Score\left(T\left(X,e_{f},e_{c}\right)\right)\text{,}$$

where *Score*() is a metric to evaluate the output of the given model, where a higher value represents a better output. We also may not need to have *e*_*f*_ and *e*_*c*_ for every model.

### Method explanation

By default, our framework utilizes embeddings generated from a closed-source OpenAI model. The design of the *AVG* strategy is motivated by prior work^18^ and informed by the intrinsic noise characteristics of single-cell RNA sequencing (scRNA-seq) data.^30^ By compressing high-dimensional gene expression profiles into a lower-dimensional space, we can jointly encode both quantitative expression levels and functional knowledge of genes.

We hypothesize and empirically demonstrate that these enriched cell embeddings can more effectively represent cellular identity by integrating external gene function annotations and cell-type-specific information derived from LLMs. As a result, access to raw expression data becomes unnecessary for many downstream analyses. This hypothesis is further supported by a recent systematic evaluation of LLM embeddings in medical machine learning.^31^ In our benchmarking, the weighted average (*wa*) mode outperformed other approaches in clustering and batch effect correction tasks. We attribute this to the importance of capturing both individual gene contributions and collective gene group effects when constructing cell embeddings in a zero-shot learning framework. For fine-tuning scenarios, task-specific methods offer more flexible designs to incorporate LLM-derived embeddings as prior knowledge, enabling further performance optimization tailored to specific biological questions.

### scELMo under the zero-shot learning framework

To evaluate the performance of *e*_*cells*_ under the zero-shot learning framework, we consider three tasks: clustering, batch effect correction, and cell-type annotation based on a k-nearest-neighbor (kNN) classifier. In this section, T is defined as a kNN classifier.

For clustering and batch effect correction, we directly use *e*_*cells*_ as a new representation for *X* and evaluate *e*_*cells*_ for these two tasks. For cell-type annotation based on a kNN classifier, we consider training dataset *X*_*train*_ and testing dataset *X*_*test*_, and their corresponding cell embeddings $e_{cells}^{train}$ and $e_{cells}^{test}$. We use $e_{cells}^{train}$ with its cell types to train a kNN classifier and perform cell-type annotation based on $e_{cells}^{test}$. Since kNN is based on similarity searching, we treat this method as an ability of zero-shot learning. We follow the settings from GenePT for this classifier and set *k* = 10.

### scELMo under the fine-tuning framework

To evaluate the performance of *e*_*f*_ and *e*_*c*_ under the fine-tuning framework, we considered three tasks: cell-type annotation, *in silico* treatment analysis, and perturbation analysis with task-specific models as adaptors.

For the cell-type annotation and *in silico* treatment analysis tasks, we present a light-structured neural network with a contrastive learning^32^^,^^33^ design. Here, T is a neural network with rectified linear units (ReLU^34^) as the activation function. Our intuition comes from the requirement for a good representation of cells with different labels and conditions. Therefore, we formalize the loss function of our model as

$$L_{total}=L_{classifier}+\lambda L_{contrastive}\text{,}$$

where $L_{classifier}$ represents the classification loss of the model output, as we use cell-type labels for model training, and $L_{contrastive}$ represents the contrastive learning loss we use to distinguish the representations of cells under different conditions in the latent space. *λ* is a hyper-parameter, where we set *λ* = 100 in this manuscript to assign a larger weight for label-aware clustering. We utilize the embeddings after fine-tuning as the training and testing datasets and use a kNN classifier to annotate the cell types to evaluate the representation we learn based on scELMo.

To analyze the target of *in silico* treatment, we first compute the cosine similarity (*CS*_*old*_) between the average cell embeddings based on the diseased case and the control case. Then, we delete the target gene by setting its expression profile to zero and compute the new embeddings and cosine similarity (*CS*_*new*_). We define the score of our targeted gene *g* as

$$Score\left(g\right)=CS_{new}-CS_{old}\text{.}$$

If such a score is larger than 1e−4, we treat the gene we analyze as a candidate for therapeutic targets. This threshold is based on the upper bound of the tiny quantities determined by Numpy^35^ for scientific notation representation and the smallest non-zero scale of the *y* axis in Figures 4A and 4B. We utilize the selected genes to run gene pathway analysis based on Gene Ontology Enrichment Analysis (GOEA)^36^^,^^37^^,^^38^ and Ingenuity Pathway Analysis (IPA).^39^

For the perturbation analysis task, we consider three different models for the three tasks. Here, T represents different models corresponding to different perturbation analysis tasks. For the causal factor analysis task and CINEMA-OT, we replace the original input of CINEMA-OT with *e*_*cells*_. We follow the default settings of CINEMA-OT for processing related datasets. For the gene expression prediction task and CPA, we add an additional neural network component to make *e*_*cells*_ learnable and combine the output of this component with the latent space from the original CPA. We do not modify the training process of CPA. We follow the default settings of CPA for processing related datasets. For the gene expression prediction task based on perturb-seq-based datasets and GEARS, we add the *e*_*f*_ to the original gene embeddings of GEARS. We do not modify the training process of GEARS. We follow the default settings of GEARS for processing Dixit, Norman, Adamson, and Replogle datasets.

### Data pre-processing

We follow the data pre-processing steps from Scanpy^29^ for scRNA-seq datasets. For single-cell proteomic datasets, we follow the pre-processing steps from TotalVI^40^ and MARIO^41^ and do not change the distribution of the original data because of its density.

#### Metrics

To evaluate the hallucinations of LLM outputs, we consider two metrics: the bilingual evaluation understudy (BLEU)^42^ score and the Human-Eval score.^43^

The BLEU score is used to evaluate the similarity between observed string $\hat{y}$ and ground-truth string *y* based on the *n*-*grams* strategy. Considering we have a function *C*(*s*,*y*) to generate the number of appearances of *s* as a substring of *y* and a set $G_{n}\left(\hat{y}\right)$ as the *n*-*grams* set, the BLEU score is defined as

$$BLEU=\frac{\sum_{s\in G_{n}\left(\hat{y}\right)}\;\min\left(C\left(s,\hat{y}\right),C\left(s,y\right)\right)}{\sum_{s\in G_{n}\left(\hat{y}\right)}C\left(s,\hat{y}\right)}\text{.}$$

The score is in [0,1], and a higher value means better performance.

The Human-Eval score means we compare the truthfulness between the LLM outputs and references from NCBI and GeneCard databases to assign scores for the string pairs. We assign 1 if the outputs contain the correct information and 0 if the outputs do not contain the correct information. We have one human expert for evaluation. A higher value means better performance.

For the evaluations of clustering and batch effect correction, we utilize the metrics described and implemented by scIB.^44^ We compute all the metrics we could in the evaluation process. All of the scores of the metrics in scIB are in [0,1], and a higher value means better performance.

For clustering, we use normalized mutual information (NMI), adjusted Rand index (ARI), and average silhouette width (ASW)_*label*_ for evaluation. For batch effect correction, we compute ASW_*batch*_, principal-component (PC) regression (PCR), graph connectivity (GC), kBET, and integration LISI (iLISI) and average the scores from these metrics to generate *S*_*batch*_. We compute ASW_*label*_, NMI, ARI, and cell-type LISI (cLISI) and average the scores from these metrics to generate *S*_*bio*_. Details of these metrics, modified based on Liu et al.^16^ and Luecken et al.,^44^ are introduced here.

(1) NMI: NMI is a score to evaluate the performance of biological information conservation. We compute this score based on the mutual information between the optimal Leiden clusters and the known cell-type labels and then take the normalization. NMI ∈ (0,1), and a higher NMI means better performance.
(2) ARI: ARI is a score to evaluate the performance of biological information conservation. ARI is used to evaluate the agreement between optimal Louvain clusters and cell-type labels. ARI ∈ (0,1), and a higher ARI means better performance.
(3) ASW: We have cell-type ASW (*ASW*_*cell*_) and batch ASW (*ASW*_*batch*_) for this metric. For one cell, ASW calculates the ratio between the inner cluster distance and the intra-cluster distance for this cell. Therefore, higher *ASW*_*cell*_ means better biological information conservation, and lower *ASW*_*batch*_ means better batch effect correction. To make them consistent, for *ASW*_*cell*_, we take the normalization, that is,

$$ASW_{cell}=\frac{ASW_{cell}^{raw}+1}{2}\text{.}$$

Similarly, for *ASW*_*batch*_, we take the inverse value of the normalized result, that is,

$$ASW_{batch}=1-\frac{ASW_{batch}^{raw}+1}{2}\text{.}$$

Both metrics are in (0,1), and a higher score means better model performance.

(4) Local inverse Simpson’s index (LISI): LISI is a metric to evaluate whether datasets are well-mixed under batch labels (*iLISI*) or can be discerned with different cell types (*cLISI*). We first compute the kNN list of one cell and count the number of cells that can be extracted from the neighbors before one label is observed twice. Furthermore, we take the normalization for *iLISI* with *B* batches, that is,

$$iLISI=\frac{iLISI^{raw}-1}{B-1}\text{.}$$

Similarly, for *cLISI* with *C* cell types, we take the inverse value of the normalized result, that is,

$$cLISI=1-\frac{cLISI^{raw}-1}{C-1}\text{.}$$

Both of metrics are in (0,1), and a higher score means better model performance.

(6) GC: GC measures the connectivity of cells in different cell types. If the batch effect is substantially removed, the connectivity of cells of the same cell type from different batches will have a higher connectivity score based on the kNN graph. Therefore, we can compute the GC score for each cell type and take the average. GC score is in (0,1), and a higher score means better batch effect correction performance.
(6) PCR: PCR is a metric to evaluate the performance of batch effect correction. We calculate the R2 for a linear regression of the covariate of interest onto each PC. The variance contribution of the batch effect for all the PCs is based on the sum of the product between the variance of each PC and the R2 of each PC across all PCs. Therefore, the score can be represented as

$$PCR=\sum_{i=1}^{G}\text{Var}\;\left(C|{\text{PC}}_{i}\right)\times R^{2}\left({\text{PC}}_{i}|B\right)\text{,}$$

where *G* denotes the number of PCs and *B* denotes the batch information. PCR is in (0,1), and a higher score means better performance.

(7) kBET: The kBET algorithm is used to determine if the label composition of the kNNs of a cell is similar to the expected label composition. For the batch label mixture of cells in the same cell types, the proportion of cells from different batches for the neighbors of one cell should match the global-level distribution. The kBET score ∈ (0,1), and a higher score means better batch effect correction performance.

For the evaluations of cell-type annotation, we use Scikit-learn^45^ to calculate accuracy, precision, recall, and F1 score by comparing the predicted cell-type labels and ground-truth cell-type labels. All of the metrics are in [0,1], and a higher value means better performance.

For the evaluations of *in silico* treatment analysis, we use Scipy^46^ to compute the cosine similarity between the mean cell embeddings from the control case and the mean cell embeddings from the diseased case. The definition of the score here is described in the methods.

For the evaluations of perturbation analysis, we have three different tasks with different metrics. For the causal factor analysis task, the metrics are the same as those we use in the batch effect correction task. For the gene expression prediction task based on CPA, we use the R2 score as a metric to evaluate the performance for regression. The R2 score is defined as

$$R2=1-\frac{SS_{\text{res}}}{SS_{\text{tot}}}=1-\frac{\sum_{i}{\left(y_{i}-f_{i}\right)}^{2}}{\sum_{i}{\left(y_{i}-\bar{y}\right)}^{2}}\text{,}$$

where *y*_*i*_ represents the ground-truth gene expression level, *f*_*i*_ represents the predicted gene expression level, and $\bar{y}$ represents the average expression levels of the given gene. A higher average R2 score and a lower variance mean better performance.

For the evaluation of gene expression prediction tasks based on GEARS, we use the Pearson correlation coefficient (PCC) and mean squared error (MSE) as metrics. We define PCC as

$$PCC=\frac{1}{m}\sum_{i}^{m}\frac{\text{cov}\left(y_{i},f_{i}\right)}{\sigma_{y_{i}}\sigma_{f_{i}}}\text{,}$$

where *m* represents the number of used genes, cov represents the covariance, and *σ* represents the standard deviation. *ρ* is in [0,1], and a higher value means better performance. We can also define MSE as

$$MSE=\frac{1}{mn}\sum_{i}^{m}\sum_{j}^{n}{\left(y_{ij}-f_{ij}\right)}^{2}\text{,}$$

where *y*_*ij*_ represents the ground-truth gene expression level of gene *i* in cell *j* and *f*_*ij*_ represents the predicted gene expression level of gene *i* in cell *j*. A lower MSE score means better performance.

We consider computing these two metrics for both the all-genes case and the top 20 differentially expressed genes (DEGs) case.

### Explanations of baseline models

For tasks related to description generation, we consider MetaPrompt^47^ and chain of thought (COT)^47^ as baseline models for prompt engineering. MetaPrompt introduces a system prompt for LLMs and generates the outputs conditioned on the context in the system prompt. COT allows LLMs to obtain complex reasoning capabilities by allowing models to address the problem with intermediate steps.

For tasks related to clustering, we consider principal-component analysis (PCA) (raw),^45^ GenePT,^18^ SC3,^48^ and scVI^49^ as baseline models. PCA is widely used for dimension reduction of single-cell data. The principles of GenePT are summarized in the introduction. SC3 utilizes consensus clustering for analyzing scRNA-seq data, which is based on aggregating the *k*-means results after PCA transformation to generate the consensus, and then performs the clustering based on consensus. scVI utilizes a generative model to learn the embeddings in the latent space of scRNA-seq data.

For tasks related to batch effect correction, we consider PCA (raw), GenePT, MARIO,^41^ Harmony,^50^ and MNN^51^ as baseline models. MARIO considers both the shared features and distinct features for proteomic data and performs paired matching for cells from different batches to generate integrated results in the latent space. Harmony assigns labels for different cells with a soft-clustering method, computes the centroids for different clusters, and updates cell embeddings based on the soft cluster membership. MNN utilizes mutual nearest neighbors to learn the relationships for cells in different batches and updates the cell embeddings based on the relationships.

For tasks related to cell-type annotation, we consider GPT-2,^52^ GPT-4,^22^ scGPT,^7^ Geneformer,^8^ GPTCelltype,^53^ multi-layer perceptron (MLP), and PCA for evaluation. To evaluate GPT-2 and GPT-4, we transfer the cell information into a sentence based on the rank of genes for each cell and treat this task as a question-answer task. scGPT is a pre-training-based FM for multiple tasks in single-cell research. The authors utilized multi-layer transformers to construct the model architecture and pre-trained scGPT based on large-scale scRNA-seq datasets. They fine-tuned scGPT to address problems in downstream applications such as cell-type annotation. Geneformer is also a model based on pre-training. The authors utilized BERT^54^ as a base model and pre-trained BERT from the sketch using scRNA-seq data transferred into sentences. They also fine-tuned Geneformer to address problems in downstream applications, including cell-type annotation, *in silico* treatment analysis, etc. GPTCelltype utilizes GPT-4 to extract markers of cell types to annotate cell clusters. To evaluate this model, we unify the model outputs and ground-truth cell-type labels. MLP means we fit an MLP based on gene expression profiles and cell types. We extract the embeddings from these two models and PCs based on both the zero-shot learning framework and the fine-tuning framework and utilize kNN to perform classification based on the embeddings.

For tasks of perturbation analysis, we consider CINEMA-OT,^55^ CPA,^56^ and GEARS^57^ for modification and evaluation. We included gene embeddings from GPT-3.5, NCBI (GenePT), and scGPT as inputs. CINEMA-OT is a method for separating confounding signals or causal factors from perturbations at the single-cell level. The first step is to initialize the expected matrix rank based on biwhitening.^58^ The second step is to separate confounder signals and treatment-associated signals based on independent component analysis (ICA).^45^ The last step is to match the cells based on entropy regularized optimal transport.^59^ We analyze the confounder embeddings for CINEMA-OT and follow the benchmarking pipeline mentioned in the original paper. CPA is a method for modeling the gene expression levels of scRNA-seq data with perturbations. CPA treats perturbations of cells as covariates and encodes these covariates as embeddings into the training process of a conditional variational autoencoder (CVAE). CPA can also be used to predict the gene expression levels of out-of-distribution (OOD) samples. GEARS is a method for predicting gene expression levels of perturb-seq-based datasets. It models the perturbations of genes based on knowledge graphs and gene embeddings, thus utilizing graph neural networks (GNNs) and MLPs to predict the gene expression levels under gene-level perturbation.

## Results

### Overview of scELMo

Embeddings generated by LLMs are highly effective at preserving the contextual and semantic structure of their input.^60^^,^^61^^,^^62^ This property makes them compelling priors for integrating enriched biological knowledge into downstream analyses. The core concept of scELMo is to map cellular information from the sequencing data space into an LLM-derived embedding space. This transformation is accomplished by incorporating metadata at either the feature level (e.g., genes and proteins) or the cell level (e.g., cell types and cell states). For feature-level integration, we extract functional information for each feature either from curated databases such as NCBI or by prompting an LLM to summarize relevant biological knowledge. The textual summaries are then passed through the LLM’s embedding layers to obtain feature representations in the embedding space. In this study, we use GPT-3.5 to both summarize feature functions and generate embeddings, based on its superior performance in our benchmarking analyses.

**Figure 1:**
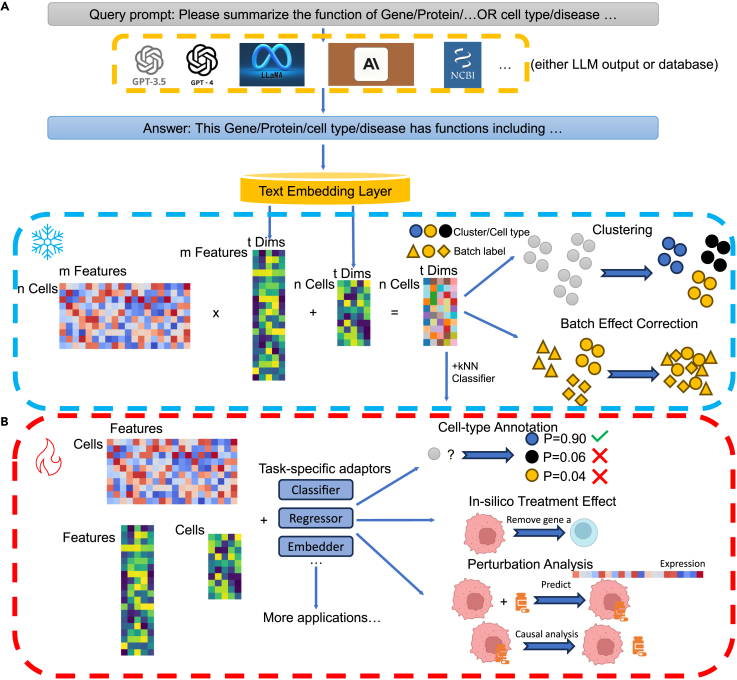
Workflow of scELMo (A) Zero-shot learning (denoted as ice) framework of scELMo. We extract the text description of metadata by using either databases or LLMs. The prompts are adjustable. We use GPT-3.5 to generate the embeddings of the text descriptions as the embeddings of features (including genes, peaks, etc., which are biological features from cell profiles) or cell states. We then aggregate these embeddings with single-cell profiles to generate cell embeddings. (B) Fine-tuning (denoted as fire) framework of scELMo. We combine embeddings of metadata and single-cell profiles with task-specific adaptors and train the adaptors to address downstream applications.

Once embeddings are extracted from LLMs and transcriptomic profiles are obtained from single-cell data, they can be integrated under a zero-shot learning framework to generate enriched cell embeddings. These integrated embeddings—constructed via matrix operations that combine LLM-derived feature embeddings with cellular expression profiles—can be directly used for tasks such as cell clustering or batch effect correction. Given recent evidence highlighting the benefits of incorporating biological knowledge into single-cell modeling,^7^^,^^63^^,^^64^ we also explore a fine-tuning framework. In this setup, LLM-derived embeddings serve as enhanced biological priors that are fed into task-specific models, which are subsequently retrained for downstream applications. This approach provides greater flexibility and allows for improved performance on task-specific objectives by leveraging the contextual richness of LLM-generated embeddings. Figure 1 provides an overview of scELMo under both the zero-shot and fine-tuning paradigms. Detailed implementations for each framework are described in the methods.

### Evaluation of hallucinations in LLMs

**Figure 2:**
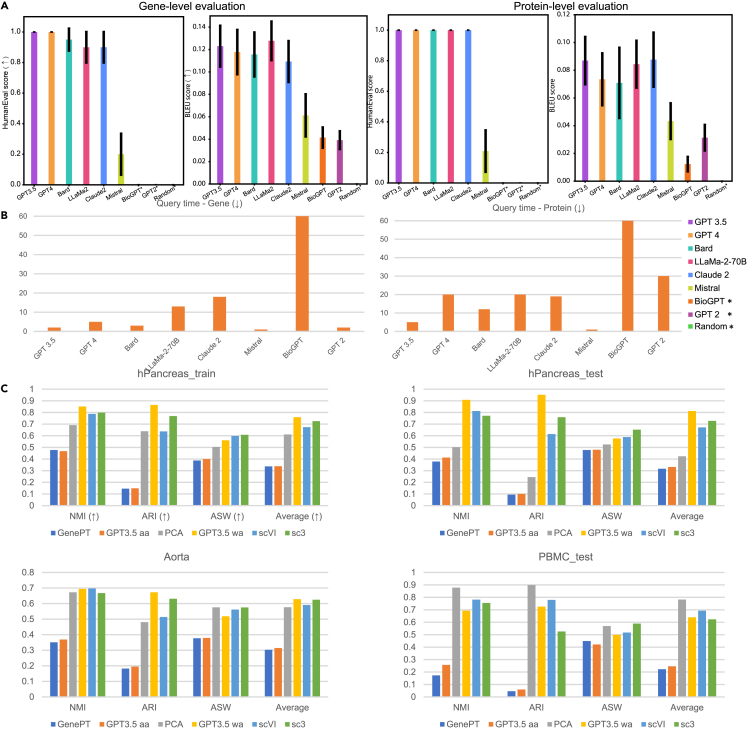
Evaluations of the outputs of LLMs and the clustering performance (A) Metrics for evaluating meaningful outputs of biological features across different LLMs. The left image represents the BLEU score and Human-Eval score of genes, while the right image represents the BLEU score and the Human-Eval score of proteins. (B) Average query time for each LLM. The left image represents the query time of genes, and the right image represents the query time of proteins. (C) Evaluations of the clustering performance based on major methods. Different images represent the results of different datasets. ∗ represents that the selected method has a zero Human-Eval score.

The first step in our pipeline is to select an appropriate LLM for generating both textual descriptions and corresponding embeddings. An ideal LLM for this application should minimize hallucinations,^65^ i.e., avoid generating fabricated or incorrect biological information based on the provided feature or cell metadata. To this end, we evaluated several candidate models, including GPT-2,^52^ GPT-3.5, GPT-4, LLaMA-2 (70B), Mistral,^66^ BioGPT,^67^ Claude 2,^68^ and Bard (PaLM 2).^69^ After considering factors such as token diversity, accuracy, and accessibility to embedding layers, we selected GPT-3.5 as the default model for generating embeddings. To benchmark the quality of its textual outputs, we randomly sampled 20 proteins from a known list (∼200 total)^70^ and 20 genes from the NCBI database (out of ∼30,000 genes) and prompted each of the listed LLMs to generate functional descriptions for these features. We assessed the correctness of the outputs by comparing them to curated references from GeneCards^71^ and NCBI, using both the BLEU^42^ score and human evaluation.^43^ BLEU measures the degree of *n-gram* overlap between the LLM outputs and human-written references, providing an estimate of textual similarity and accuracy. The evaluation results for genes and proteins are shown in Figure 2A, with corresponding query times per model reported in Figure 2B. Additional results for cell-type descriptions are provided in Figure S1A.

Our evaluation showed that GPT-3.5 and GPT-4 produced the most accurate functional descriptions of biological features. However, compared to GPT-3.5, GPT-4 incurred significantly longer response times and occasionally failed to comply with the required prompt formatting. To further assess the impact of prompt design, we explored additional prompting strategies, including MetaPrompt^47^ and COT^72^ prompting. Neither approach improved BLEU scores relative to the default prompt, as shown in Figure S1B. In fact, COT and MetaPrompt led to lower Human-Eval scores compared to the original format, indicating reduced alignment with expert-curated references. To expand our analysis, we scaled the evaluation to 100 randomly selected genes and computed BLEU scores by comparing LLM-generated descriptions against NCBI entries. GPT-3.5 continued to outperform other models, yielding the highest BLEU score with low variance, followed by Claude (Figure S2). Factoring in both description accuracy and runtime performance, we selected GPT-3.5 as the default LLM for generating feature descriptions. The results shown in Figure S1A further confirm GPT-3.5’s capability in generating accurate cell-type descriptions.

We also assessed the stability and biological specificity of GPT-3.5-derived embeddings. As shown in Figure S3A, the cosine similarity between embeddings generated across multiple GPT-3.5 responses for the same gene exceeded 0.9, indicating high consistency. By contrast, pairwise cosine similarity between embeddings from distinct genes was substantially lower (range: 0.74–0.84), demonstrating the ability of the embeddings to differentiate functionally distinct genes (Figure S3B). Additional evaluation of embedding variability using different random seeds is presented in the discussion. To further validate the functional relevance of the embeddings, we applied a kNN classifier (*k* = 10) using Geneformer’s functional gene annotations.^8^ A train-test split (80%-20%) yielded an accuracy of 0.931, underscoring the informative nature of the embeddings. Figures S4A and S4B illustrate functional clustering patterns, where genes with similar roles are embedded proximally.

To evaluate the biological coherence of these clusters, we performed GOEA^36^^,^^37^^,^^38^ and IPA^39^ on the top 10 protein-coding gene clusters, ranked by size. Across all clusters, top enriched pathways (ranked by −*log*(adjusted *p* value)) involved essential biological processes, including metabolism, transport, and genetic regulation (Figures S5 and S9). These results demonstrate that GPT-3.5-derived embeddings preserve gene functional information and support downstream analyses. Additional text analyses comparing NCBI and LLM-generated gene descriptions are detailed in Note S1, with raw outputs summarized in Data S1. We also demonstrate the applicability of LLM-derived embeddings to single-cell and spatial transcriptomics datasets across different species, highlighting potential for cross-species analyses in Note S2.

### scELMo for clustering and batch effect correction

In this section, we investigated the contribution of feature embeddings from scELMo for cell-level tasks, including clustering and batch effect correction.

Clustering provides an effective means of evaluating whether feature embeddings encode biologically relevant information. To assess this, we integrated gene or protein embeddings into single-cell sequencing data and performed clustering based on the resulting cell embeddings. We quantified clustering performance using three widely adopted metrics in single-cell analysis: NMI, ARI, and ASW.^44^ Unless otherwise noted, GenePT refers to the GenePT-w variant in the following analyses. Because we generated gene embeddings using both NCBI and Ensembl^73^ gene identifiers, our embedding set contained a larger number of genes than that used by GenePT. In GenePT, the default strategy for integrating feature embeddings into cells is a naive *aa*, where cell embeddings are obtained by computing the dot product between the gene expression vector and the matrix of gene embeddings generated by GPT-3.5. However, this approach disregards differences in the magnitude of log-normalized gene expression across cells, even though gene expression levels are known to significantly influence cellular function.^74^^,^^75^

To address this limitation, we propose an alternative approach that incorporates gene expression values as weights, computing a *wa* for each cell. This approach better captures the functional contributions of genes with varying expression levels. Both the naive and *wa* strategies were implemented for generating cell embeddings, with further details provided in the methods. Using the *wa* mode was better than using the *aa* mode for cell clustering under different metrics, as shown in Figure 2C. Meanwhile, the *wa* mode has better performances in three out of four datasets compared with the task-specific methods SC3^48^ and scVI.^49^ Moreover, using embeddings from scELMo also improved the clustering performance compared with the embeddings from GenePT, advocating the use of LLMs as a tool for summarizing scientific concepts. However, various approaches to combining the embeddings from GenePT and GPT-3.5 (e.g., GPT-3.5 + GenePT *wa* means sum by genes and GPT-3.5 ‖ GenePT *wa* means concatenation by genes) did not improve the scores compared with the individual setting. The average ranks of all methods across different datasets are summarized in Figure S6A, with the GPT-3.5 *wa* mode having the lowest rank. Finally, if we combined cell embeddings with the embeddings of cell-type information from GPT-3.5, we could get scores close to one, suggesting that the cell-type embeddings contained meaningful cell-type information. Note S3 summarizes factors that could affect clustering performance.

For the batch effect correction task, we first focused on single-cell proteomic data—specifically CITE-seq^13^ and CyTOF^12^ datasets—since GenePT had already assessed performance on small-scale scRNA-seq data. To ensure fair benchmarking, we adopted a projection-based strategy, aligning datasets from different batches into a shared embedding space without further training. Two scenarios were considered: batch correction for datasets generated using (1) the same experimental protocol and (2) distinct protocols. We compared the batch correction performance of embeddings produced by scELMo and GenePT. Evaluation was conducted using scIB metrics,^44^ separating the contribution to batch effect removal (*S*_*batch*_) from the preservation of biological variation (*S*_*bio*_). A detailed description of these metrics is provided in the methods. Additionally, we benchmarked against task-specific batch correction methods, including MARIO,^41^ Harmony,^50^ and MNN.^51^

**Figure 3:**
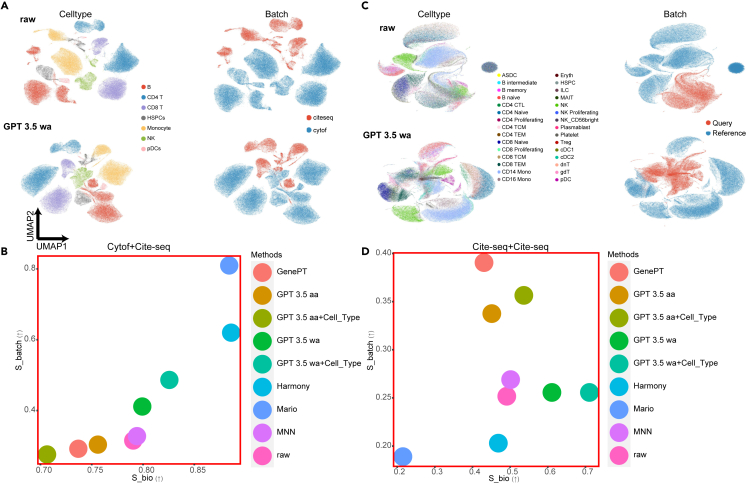
Results of batch effect correction for single-cell proteomic data (A) Uniform manifold approximation and projections (UMAPs)^76^ for the cell-type information (left) and batch information (right) of CITE-seq-based datasets. The top image represents the raw data. The bottom image represents the cell embeddings from GPT-3.5 *wa* mode. (B) Evaluations of the batch effect correction for CITE-seq-based datasets across different methods. (C) UMAPs for the cell-type information (left) and batch information (right) of CITE-seq-based dataset and CyTOF-based dataset. The top image represents the raw data. The bottom image represents the cell embeddings from GPT-3.5 *wa* mode. (D) Evaluations of the batch effect correction for CITE-seq-based dataset and CyTOF-based dataset across different methods.

Results for two CITE-seq datasets are shown in Figures 3A and 3B, and the results for a combined CITE-seq and CyTOF dataset are shown in Figures 3C and 3D. In both scenarios, the naive *aa* approach failed to improve batch correction performance for scELMo, a trend consistent across task-specific methods. Although MARIO exhibited strong performance in integrating CITE-seq and CyTOF datasets, it showed no advantage when integrating two CITE-seq datasets. MNN underperformed compared to both *aa* and *wa* approaches in all cases.

Notably, the *aa* method reduced both *S*_*batch*_ and *S*_*bio*_ in the CyTOF+CITE-seq case, suggesting an increased batch effect and a diminished biological signal. In contrast, the *wa* approach consistently reduced batch effects while maintaining biological variation. Incorporating cell-type embeddings under the *aa* mode did not improve either metric. However, combining cell-type embeddings with cell embeddings under the *wa* mode enhanced *S*_*bio*_ in both scenarios, underscoring the importance of a robust base embedding space for effectively leveraging cell-state information. We extended our evaluation to atlas-scale scRNA-seq datasets,^77^^,^^78^^,^^79^ and as shown in Figure S7, scELMo outperformed GenePT in integrating large-scale transcriptomic datasets. A summary of our multi-omics data integration analysis^80^ is provided in Note S4.

### scELMo for cell-type annotation

Cell-type annotation is a fundamental task in single-cell data analysis.^81^ In this work, we evaluated the performance of scELMo for cell-type classification under two different frameworks: zero-shot learning and fine-tuning. Under the zero-shot learning framework, we incorporated gene embeddings into both the training and testing datasets to generate cell embeddings and then used the kNN classifier implemented in GenePT to assign cell types in the testing set. However, this approach failed to generalize effectively when the training data originated from heterogeneous sources or displayed pronounced batch effects, as evidenced in the peripheral blood mononuclear cell (PBMC) dataset results shown in Table 1. To address this limitation, we designed a simple neural-network-based classifier enhanced with contrastive learning,^32^ similar in spirit to adaptors in natural language processing (NLP) applications.^28^ The architecture and training rationale are provided in Figure S8 and the accompanying methods description. The model was trained on combined gene expression profiles and LLM-derived gene embeddings—thus integrating both quantitative and semantic information. We evaluated this model across three datasets from distinct tissues. For the hPancreas and PBMC datasets, all but one batch were used for training, with the held-out batch used for testing. For the Aorta dataset, we adopted the 80/20 train-test split used in the original GenePT study.^18^ These experiments enabled us to assess the effectiveness of LLM-enriched embeddings for cell-type annotation across varying tissue types and dataset complexities.

**Table 1:** Scores of cell-type annotation task under different settings

hPancreas^82^^,^^83^^,^^84^^,^^85^	Accuracy (↑)	Precision (↑)	Recall (↑)	F1
**Zero-shot settings**				

GPT-2 query	0.000	0.000	0.000	0.000
GPT-4 query	0.000	0.000	0.000	0.000
scGPT (z)	0.770	0.610	0.560	0.550
Geneformer (z)	0.500	0.250	0.340	0.270
GenePT-w	0.940	0.720	0.630	0.650
GenePT-s	0.890	0.650	0.530	0.560
GPTCelltype	0.501	0.386	0.415	0.366
PCA	0.633	0.369	0.382	0.357
GPT3.5 *aa*	0.933	0.702	0.614	0.629
GPT3.5 *wa*	0.933	0.702	0.613	0.629

**Fine-tuning settings**				

scGPT	**0.970**	0.742	**0.758**	**0.741**
Geneformer	0.321	0.105	0.094	0.086
scELMo+GenePT	0.963	**0.790**	0.662	0.680
scELMo+random emb	0.968	0.683	0.693	0.680
scELMo+GPT-3.5	**0.970**	0.769	0.674	0.687
Aorta^86^	Accuracy	Precision	Recall	F1

**Zero-shot settings**				

GPT-2 query	0.000	0.000	0.000	0.000
GPT-4 query	0.000	0.000	0.000	0.000
scGPT (z)	0.938	0.921	0.894	0.901
Geneformer (z)	0.860	0.700	0.600	0.620
GenePT-w	0.870	0.910	0.680	0.720
GenePT-s	0.860	0.700	0.600	0.620
GPTCelltype	0.340	0.254	0.261	0.228
PCA	0.929	0.923	0.911	0.910
GPT3.5 *aa*	0.877	0.867	0.678	0.719
GPT3.5 *wa*	0.877	0.867	0.677	0.718

**Fine-tuning settings**				

scGPT	**0.962**	0.938	0.939	0.937
Geneformer	0.340	0.087	0.092	0.080
scELMo+GenePT	0.958	**0.952**	**0.940**	**0.946**
scELMo+random emb	0.951	0.913	0.874	0.882
scELMo+GPT-3.5	0.957	0.950	0.936	0.942
PBMC^87^^,^^88^^,^^89^^,^^90^	Accuracy	Precision	Recall	F1

**Zero-shot settings**				

GPT-2 query	0.000	0.000	0.000	0.000
GPT-4 query	0.000	0.000	0.000	0.000
scGPT (z)	0.915	0.781	0.803	0.789
Geneformer (z)	0.126	0.128	0.130	0.095
GenePT-w	0.286	0.607	0.270	0.315
GenePT-s	–	–	–	–
GPTCelltype	0.501	0.387	0.415	0.366
PCA	0.436	0.392	0.278	0.285
GPT3.5 *aa*	0.190	0.564	0.181	0.230
GPT3.5 *wa*	0.190	0.562	0.181	0.231

**Fine-tuning settings**				

scGPT	0.933	0.798	0.822	0.807
Geneformer	0.235	0.146	0.147	0.131
scELMo+GenePT	0.919	0.785	0.823	0.801
scELMo+random emb	0.903	0.854	0.826	0.835
scELMo+GPT-3.5	**0.955**	**0.940**	**0.929**	**0.934**

Parts of the results are directly extracted from GenePT. Here, PCA represents principal-component analysis, and scELMo+random emb represents fine-tuning scELMo with random numbers as meaningless gene embeddings. Average ranks of all methods across datasets are summarized in Figure S6B. The highest score of each metric for each dataset is set in bold.

Table 1 demonstrates that GPT-3.5-derived embeddings exhibit strong zero-shot learning capabilities for cell-type annotation in both the hPancreas and PBMC datasets. Notably, scELMo outperformed GPTCelltype,^53^ a method that relies on LLM-extracted marker genes for annotation. We attribute this improvement to GPTCelltype’s dependency on pre-clustering and pseudo-labeling steps prior to LLM-based annotation, which can reduce marker quality—particularly in complex datasets with subtle or overlapping cell-type signatures.

In contrast, scELMo enhances cell representation by directly integrating gene-level LLM embeddings, leading to improved accuracy in cell-level tasks such as annotation. We also found that representing cells using only ranked gene expression or embeddings from GPT-2 or GPT-4 resulted in substantially inferior performance. These observations suggest that while zero-shot cell-type annotation is feasible using LLM-derived gene embeddings, its success is contingent upon the level of batch heterogeneity in the dataset. For example, in the PBMC dataset containing samples from multiple sources, the zero-shot performance lagged behind the results obtained via fine-tuning. Importantly, fine-tuning scELMo’s adaptor with GPT-3.5 or GenePT embeddings yielded performance comparable to state-of-the-art FMs such as scGPT^7^ and Geneformer.^8^ However, both scGPT and Geneformer require substantially higher computational resources (e.g., NVIDIA A100 GPUs) and longer fine-tuning times,^16^ as shown in Figures S10A and S10B. Across all tested datasets, the fine-tuning framework of scELMo consistently outperformed the zero-shot learning framework, with the added benefit of adapting to embeddings from various sources.

Overall, our results underscore that fine-tuning an adaptor model using gene embeddings from LLMs enables accurate, resource-efficient cell-type annotation, making scELMo both practical and scalable for diverse single-cell datasets.

### scELMo for *in silico* treatment analysis

**Figure 4:**
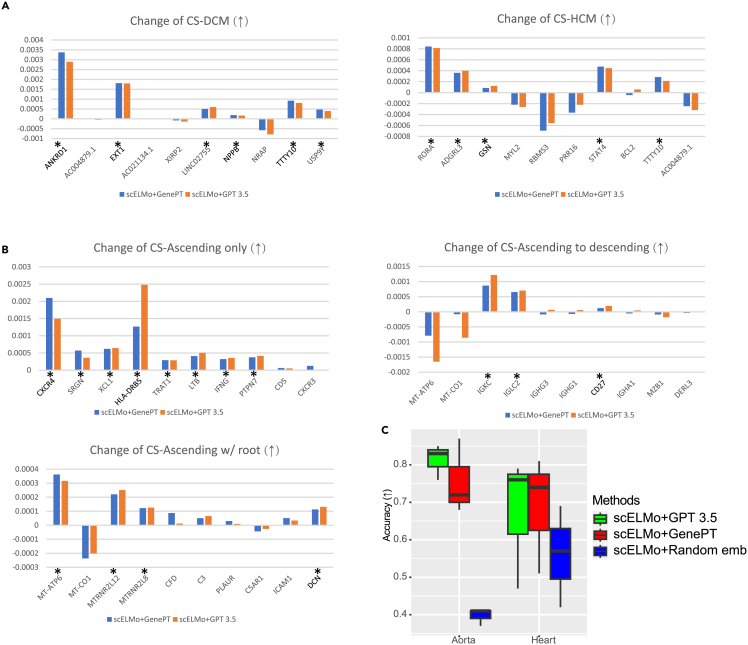
Results of *in silico* treatment analysis A gene is treated as a potential therapeutic target if the change of cosine similarity (CS) by removing the gene is larger than 1e−4. The reason for setting such a threshold is discussed in the methods. We chose the top 10 DEGs as candidates. (A) The change of CS by removing different genes in the expression space for hypertrophic (HCM) or dilated cardiomyopathy (DCM) states. (B) The change of CS by removing different genes in the expression space for ascending aortic aneurysm (ascending only, ascending to descending, and ascending with root). (C) The accuracy of disease-state annotation under different settings of scELMo. We highlighted the genes detected by both GenePT and scELMo using asterisks (∗) and marked the genes that were discovered by previous research as therapeutic targets using bold type.

Computational methods for therapeutic target discovery and drug development have garnered significant interest.^91^^,^^92^^,^^93^ In this section, we extend the functionality of scELMo by combining its adaptor-based cell-type annotation model with gene embeddings from GPT-3.5 or NCBI to model human diseases and identify candidate therapeutic targets. This represents a novel direction not previously explored in GenePT. Inspired by Geneformer,^8^ we fine-tuned our adaptor model on a cell-condition classification task and used the resulting cell embeddings to reveal potential molecular targets. A train-validation split was employed to select the optimal adaptor configuration, and the model was then evaluated by systematically removing DEGs between disease and control conditions to observe changes in cell embedding profiles. A detailed discussion on the role of fine-tuning in this context is provided in Note S5. As shown in Figure 4C, embeddings derived from GPT-3.5 consistently yielded the most accurate results. Specifically, when a candidate therapeutic target was removed, cell embeddings under the disease condition became more similar to those under the control condition—an outcome quantified using cosine similarity between disease-specific embeddings and the mean embedding of control cells. This direct embedding-based metric enables *in silico* prioritization of therapeutic targets. We further validated the identified targets using GOEA and literature review, with the findings summarized in Data S2. Overall, these results illustrate how scELMo can facilitate computational disease modeling and target discovery by leveraging LLM-derived gene embeddings and fine-tuned adaptation mechanisms.

We first investigated hypertrophic and dilated cardiomyopathy (HCM and DCM, respectively)^94^ using a scRNA-seq dataset derived from human heart tissue.^95^ By simulating *in silico* gene deletions under disease conditions, we identified genes whose removal led to significant shifts in cell embeddings toward the non-failing (NF) control state. These changes were quantified by comparing cosine similarity values before and after *in silico* deletion. The results, summarized in Figure 4A, show that scELMo identified two putative therapeutic targets for DCM and four for HCM. Notably, gene embeddings derived from both GPT-3.5 and NCBI sources yielded consistent predictions. The *in silico* silencing of these genes resulted in embedding-level shifts resembling cells under control conditions, suggesting their potential as therapeutic targets. Literature review supports the involvement of several identified genes in cardiomyopathy pathology, including ANKRD1,^96^ EXT1,^97^ NPPB,^98^ and TTTY10.^99^ In addition, GSN has been validated as a therapeutic target using CRISPR-based approaches^100^ and was also recovered by our model, consistent with prior findings.^8^ We further validated the functional relevance of the predicted targets via GOEA and IPA. Figures S11A and S11B display enriched pathways—each with at least ten significantly enriched terms ranked by −*log*(adjusted *p* value)—while the IPA results are summarized in Figure S11C. These analyses highlight key roles of the selected genes in cardiac muscle contraction, ion transport, and other pathways central to heart physiology, providing biological plausibility for their role in disease modulation. Together, these findings demonstrate the potential of scELMo for *in silico* therapeutic target discovery by integrating LLM-derived gene embeddings with condition-specific cellular embeddings.

Next, we investigated ascending aortic aneurysm,^101^ which comprises three distinct disease states. For this analysis, we used the scRNA-seq Aorta dataset and applied the same *in silico* gene deletion strategy as described above. The resulting changes in cosine similarity between disease and control embeddings are summarized in Figure 4B. Using scELMo, we identified six candidate therapeutic genes for the ascending-only state, two for the ascending-to-descending state, and three for the ascending-with-root-involvement state. These predictions are visualized in Figure 4B. Notably, MT-ATP6 was highlighted as a potential target across states. This mitochondrial gene has previously been linked to variable rates of cell death^102^ and neurodegeneration,^103^ suggesting that cells in distinct aneurysm states may exhibit differing susceptibilities to degeneration or stress. We further investigated the biological relevance of the selected genes using GOEA, with the results shown in Figures S11C–S11E, and IPA, summarized in Figure S12B. Consistent across analyses, the identified genes were significantly enriched in pathways associated with immune responses. These findings highlight the potential mechanistic links between immune dysregulation and aneurysm progression and underscore the value of scELMo in uncovering gene-disease relationships with clinical relevance.

### scELMo for perturbation analysis

Analyzing the effects of perturbations on cell states using single-cell data is a critical task in modern biology. Since gene embeddings generated by LLMs encode functional information about potential perturbation targets, we sought to investigate how scELMo could be applied to perturbation modeling and analysis. To this end, we focused on three distinct tasks spanning both chemical perturbations^104^ and gene-level perturbations (e.g., CRISPR-based perturb-seq).^105^ Our strategy was to leverage cell or gene embeddings generated under the zero-shot learning framework of scELMo, either as replacements for or complements to the original inputs of existing perturbation modeling methods. We evaluated three representative tasks: (1) causal factor analysis using CINEMA-OT,^55^ (2) gene expression prediction under chemical perturbation using CPA,^56^ and (3) gene expression prediction under gene-level perturbation using GEARS.^57^ In each case, we assessed the contribution of scELMo by comparing performance using the original model inputs vs. inputs augmented or replaced with scELMo-derived embeddings. Evaluation metrics were consistent with those defined by the respective methods.

**Figure 5:**
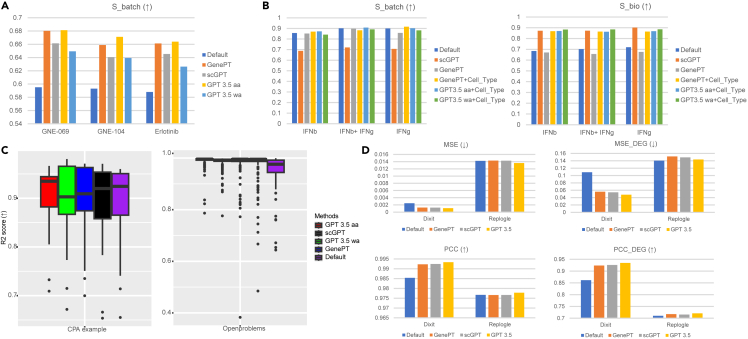
Results of perturbation analysis (A) Scores of causal factor analysis for the Chang et al. dataset^106^^,^^107^ based on CINEMA-OT across different input data. We considered four different types of extension. We still use *S*_*batch*_ to represent the levels of perturbation effect removal because we used the metrics for benchmarking batch integration. (B) Scores of causal factor analysis for perturbed PBMC dataset^55^ based on CINEMA-OT across different input data. (C) Scores of gene expression prediction under different perturbation cases based on CPA. We considered four different methods and two datasets. (D) Scores of gene expression prediction using perturb-seq datasets based on GEARS. We considered three different methods and three datasets.

CINEMA-OT is a causal learning framework based on optimal transport that separates perturbation effects from intrinsic cell-state effects in scRNA-seq datasets. To evaluate the contribution of our embeddings, we replaced CINEMA-OT’s default input (PCA features) with cell embeddings from scELMo. First, in the intrinsic cell-state (confounder) space where perturbation effects are removed, embeddings from the same cell type should not differ by perturbation cases. Using our cell embeddings as input improved CINEMA-OT’s performance compared with PCA, leading to reduced batch effects for cells of the same type, as shown in Figure 5A. Furthermore, in this setting, GenePT gene embeddings outperformed gene embeddings derived from GPT-3.5. Second, we examined whether gene embeddings alone are sufficient for causal factor analysis across different cell types. As shown in Figure 5B, relying solely on gene embeddings did not significantly improve the separation of perturbation effects and intrinsic cell states. In contrast, when we integrated both gene-level and cell-type embeddings, the modified CINEMA-OT achieved substantially better separation across perturbation cases. This improvement was supported by Wilcoxon rank sum tests comparing GenePT (default) and scELMo embeddings, yielding significant differences in two datasets (*p* = 0.031 for both cases).

Moreover, Figures S13A and S13B show that the visualization could also be improved by using the updated embeddings as model input. Figures S13C and S13D show that incorporating the cell embeddings into CINEMA-OT did not affect the analysis of the gene synergy effect, and the difference between the synergy of monocytes and the synergy of other cell types was also obvious. For the causal factor analysis task, using the *wa* mode did not improve the score. One possible reason is that CINEMA-OT can learn the best representation of cell embeddings with a good start for optimization, so the *aa* mode is adequate. Therefore, scELMo can improve the performance of CINEMA-OT on the causal factor analysis task by offering another candidate of input data.

CPA is a tool based on CVAE to predict the gene expression levels for the OOD samples of scRNA-seq data under chemical perturbations. Here, we combined the gene embeddings from scELMo with the original input dataset and learned a latent space for gene expression prediction. We investigated the contribution of gene embeddings by comparing the R2 score between these two different settings. We computed the R2 score based on the predicted gene expression levels and the observed gene expression levels. Figure 5C shows the performance of CPA under different methods for two datasets. For the CPA example dataset shown on the left, using the cell embeddings from GenePT and GPT-3.5 in the training process slightly improved the average R2 score, while its median value was still lower than the default mode. For the Openproblems dataset^108^ shown on the right, using cell embeddings from scGPT, GPT-3.5, and GenePT improved the performance of CPA. Moreover, the R2 score based on combining cell embeddings with CPA had a higher average value and lower variance compared with the default mode. We further performed Wilcoxon rank sum tests for the R2 scores between different conditions and set the significant threshold for *p* values as 0.05. Based on our computation, the pairs of settings with significant differences include GPT-3.5 *aa* vs. default (*p* = 0.003) and GPT-3.5 *aa* vs. GenePT (*p* = 0.002) based on the CPA example dataset and GPT-3.5 *aa* vs. default (*p* = 7e−11) and GPT-3.5 *wa* vs. default (*p* = 6e−12) based on the Openproblems dataset. Therefore, scELMo could improve the performance of CPA on the prediction task by introducing the cell embeddings into the training process.

GEARS is a tool based on GNNs^109^ to predict the gene expression levels for perturb-seq-based datasets. Here, we combined the gene embeddings from scELMo with the original gene embeddings of GEARS to learn the predicted value of target genes. We studied the contribution of gene embeddings by comparing the PCCs and MSEs between the default settings and updated settings when all genes were considered and when only DEGs were considered. The results are summarized in Figures 5D and S14A. For both Replogle^110^ and Dixit^105^ datasets, scELMo based on gene embeddings from GPT-3.5 outperformed the default settings as well as scELMo based on gene embeddings from GenePT and scGPT, supported by the comparisons based on all the metrics. For the Dixit dataset, using gene embeddings from GPT-3.5 obviously improved the gene expression prediction made by GEARS for both the all-genes case and the DEGs case. By considering the four datasets (Dixit, Norman,^111^ Adamson,^112^ and Replogle^110^) together, we performed a Wilcoxon rank sum test between the PCC and the PCC_DEG of scELMo and the default mode, and the improvement of scELMo was significant (*p* = 0.027). Furthermore, we also simulated datasets with different numbers of cells based on the Dixit dataset to test the robustness and consistency of our improvement, inspired by Wenteler et al.^113^ Figure S14B shows that scELMo has an overall better performance as well as a lower variance compared with the default mode. Therefore, we believe that the introduction of gene embeddings from LLMs can also contribute to perturbation effect prediction by incorporating more knowledge about perturbed genes.

## Discussion

Modeling genetic and cellular functionality is a central challenge in computational biology. Traditionally, biological questions are addressed by designing targeted experiments. However, the advent of FMs has introduced a complementary paradigm that offers generalized, data-driven tools capable of addressing a wide range of known and unforeseen biological questions. In the context of single-cell data analysis, many recent efforts have focused on developing large-scale pre-trained models, often referred to as FMs, and validating them across diverse downstream tasks. While such models demonstrate competitive performance, it is often difficult to identify tasks that truly require resource-intensive pre-training and fine-tuning pipelines.^16^ This raises questions about the necessity and efficiency of such approaches. Motivated by GenePT, we explored an alternative strategy for building FMs in single-cell biology—leveraging the generative and representational capabilities of LLMs to produce meaningful feature- and cell-level embeddings. These embeddings can be used directly for unsupervised tasks, such as clustering and batch effect correction, or can be combined with task-specific models to enhance their performance in supervised settings. Both strategies are unified within the scELMo framework. Importantly, the gene embeddings generated by scELMo are robust to stochasticity, as demonstrated in Figure S15, where downstream performances exhibit low variance across different random seeds. Furthermore, accessing LLM-derived embeddings (e.g., via GPT-3.5) does not require substantial computational resources, making this approach highly cost effective. The comprehensive results presented here highlight the capability of scELMo to address a range of biological questions efficiently, without the need for extensive pre-training.

For scELMo under the zero-shot learning framework, we could utilize cell embeddings to perform clustering and batch effect correction. These contributions are based on the fact that embeddings from the text description of features in single-cell datasets are good for representing biological concepts or functions. We also discussed the factors that could affect the performance of generating meaningful cell embeddings, including the approach to computing the average embeddings, the number of cells in one dataset, the number of features we need, and other factors. We showed that such embeddings could be used for multi-omics data analysis, which illustrates the power of using LLMs as tools for incorporating prior information to enhance task-specific analysis.

Considering the limitations of the zero-shot learning framework, we also proposed a fine-tuning framework for scELMo. By combining the feature embeddings from GPT-3.5 with a light-structured neural network, we could use the embeddings to annotate cell types with performance similar to those of FMs that require many more resources for pre-training and fine-tuning. Moreover, scELMo can also be used for detecting novel therapeutic targets by examining the change of embeddings corresponding to the removal of certain genes, supported by related biological experiments. We could also directly incorporate the cell embeddings or feature embeddings with task-specific models for better performance in modeling the data with perturbation. *In silico* treatment analysis and perturbation analysis are two challenging and important tasks with cell-level knowledge, which further support the potential of scELMo and related work.

We provide the following guidelines for users interested in applying scELMo. For unsupervised tasks such as clustering and batch effect correction, we recommend using the *wa* mode, as it more effectively incorporates quantitative gene expression information. In contrast, the *aa* mode is better suited for perturbation analysis, where the contribution of individual features is more evenly distributed. The rationale behind these recommendations is elaborated in the methods. Both embedding strategies—*wa* and *aa*—can be effectively applied to cell-type annotation and *in silico* treatment analysis. We encourage users to consider enriching their analyses by integrating relevant metadata embeddings based on the specific biological context and task requirements. Finally, we encourage the community to further explore and expand the capabilities of scELMo, particularly in novel or underexplored domains of single-cell and systems biology, as its modular design lends itself well to broad and creative applications.

However, scELMo also has the following limitations. Firstly, the rapid development of LLMs likely leads to embeddings better than GPT-3.5. With a more powerful LLM, we will have a better representation of features and cells. Secondly, LLMs could not generate meaningful information for genes that were recently discovered or analyzed. Although GPT-3.5 does not make up concepts for genes, the lack of knowledge still presents a question for the application of scELMo. This shortcoming might limit the performance of applying scELMo for low-resource biological data, such as cells from patients with rare diseases. Finally, extracting features from other biomedical data, such as GWAS^114^ or scATAC-seq^115^ data, will be difficult since the number of features for these data is quite large.

Looking ahead, we aim to develop a comprehensive database containing textual descriptions and corresponding embeddings of biological features generated by multiple LLMs. Such a resource would enable broader application of scELMo, including its use in OOD prediction tasks, such as those handled by models such as CPA. To further address limitations observed in perturbation modeling, we plan to incorporate additional contextual information—such as perturbation-specific annotations of target genes—and explore improved evaluation metrics tailored to perturbation-related analyses. In parallel, we aim to investigate more efficient strategies for developing gene-specific LLMs, which may allow for even richer contextual embeddings with reduced resource demands. Given that scELMo is intrinsically compatible with arbitrary biomedical datasets in tabular formats, we believe its use can be generalized to a variety of domains beyond single-cell analysis. We anticipate that future extensions of scELMo will continue to unlock novel applications and insights across computational biology and related fields.

## Resource availability

### Lead contact

Further information and requests for resources and reagents should be directed to and will be fulfilled by the lead contact, Prof. Hongyu Zhao (hongyu.zhao@yale.edu).

### Materials availability

This study did not generate new or unique reagents.

### Data and code availability

The code of scELMo can be found at https://github.com/HelloWorldLTY/scELMo and has been archived at Zenodo.^116^ The license is a MIT license. To generate the text descriptions and embeddings, we rely on the API of OpenAI. To run scELMo, we rely on the Yale High-performance Computing Center (YCRC) and utilize one NVIDIA A5000 GPU with up to 30 GB RAM for fine-tuning. We utilize the bigmem node with up to 1,000 GB RAM for analysis, and the minimal RAM requirement to analyze ∼1,000,000 cells is 70 GB.

We did not generate new sequencing datasets in this project. The feature embeddings used in this manuscript can be downloaded through the scELMo embedding library.^117^ All of the data used in this study can be downloaded^18^^,^^55^^,^^57^^,^^77^^,^^78^^,^^79^^,^^87^^,^^88^^,^^89^^,^^90^^,^^95^^,^^106^^,^^108^^,^^110^^,^^118^^,^^119^ using the following links:

• https://github.com/JackieHanLab/TOSICA
• https://drive.google.com/drive/folders/1LgFvJqWNq9BqHbuxB2tYf62kXs9KqL4t
• https://academic.oup.com/bioinformatics/article/38/16/3942/6623406
• https://www.biorxiv.org/content/10.1101/2022.05.09.490241v2.abstract
• https://www.ncbi.nlm.nih.gov/geo/query/acc.cgi?acc=GSE139369
• https://www.ncbi.nlm.nih.gov/geo/query/acc.cgi?acc=GSE174072
• https://www.nature.com/articles/s41591-021-01329-2
• https://www.nature.com/articles/s41591-023-02327-2
• https://www.nature.com/articles/s41586-020-2797-4
• https://www.nature.com/articles/s41586-022-04817-8
• http://projects.sanderlab.org/scperturb/datavzrd/scPerturb_vzrd_v1/dataset_info/index_1.html
• https://github.com/vandijklab/CINEMA-OT/tree/main
• https://www.kaggle.com/competitions/open-problems-single-cell-perturbations
• https://cellxgene.cziscience.com/e/3faad104-2ab8-4434-816d-474d8d2641db.cxg/
• https://www.nature.com/articles/s41587-023-01905-6
• https://www.sciencedirect.com/science/article/pii/S0092867422005979?via%3Dihub

The download links and data statistics of all data are summarized in Data S3. The source data of the presented figures are available in Data S4.

## Acknowledgments

We thank Gefei Wang and Chen Lin for the suggestions on datasets. We thank Mingze Dong for helpful discussions. We also acknowledge ChatGPT for helping us revise the text. This project is supported in part by 10.13039/100000002NIH grants U24HG012108 and U01HG013840.

## Author contributions

T.L. designed this study. T.L., X.L., and Y.C. designed the model. T.L., T.C., and W.Z. ran all the experiments. T.L. and H.Z. wrote the manuscript. H.Z. supervised this work. We did not use AI tools to write this manuscript.

## Declaration of interests

The authors declare no competing interests.

## Supplemental information

Document S1. Figures S1–S23 and Notes S1–S5

Data S1. LLM output

Data S2. *In silico* information

Data S3. Dataset information

Data S4. Raw score

Document S2. Article plus supplemental information
