# Supplementary material for: Embeddings from language models are good learners for single-cell data analysis
Source: Patterns (N Y). 2026 Jan 30;7(2):101431. doi: 10.1016/j.patter.2025.101431 (PMC12921509; doi:10.1016/j.patter.2025.101431)
Supplement: Document S2. Article plus supplemental information [file mmc6.pdf]

Patterns

Article

Embeddings from language models are good learners for single-cell data analysis

Highlights

- scELMo leverages large language models to integrate knowledge
- scELMo supports multiple settings
- scELMo achieves superior performance with fewer resources
- scELMo establishes a scalable and interpretable framework

Authors

Tianyu Liu, Tianqi Chen, Wangjie Zheng, Xiao Luo, Yiqun Chen, Hongyu Zhao

Correspondence

hongyu.zhao@yale.edu

In brief

scELMo introduces a way to analyze massive single-cell datasets by harnessing large language models to summarize biological knowledge about each gene. These summaries are transformed into mathematical embeddings that integrate with cell data, enabling efficient cell grouping, batch correction, and treatment prediction. By merging language understanding with biological data, scELMo reduces computational demands and democratizes advanced analysis—offering a faster, more accessible path to discoveries that could inform new therapies and deepen understanding of human health.

Liu et al., 2026, Patterns *7*, 101431
February 13, 2026 © 2025 The Author(s). Published by Elsevier Inc.
https://doi.org/10.1016/j.patter.2025.101431

Patterns

CellPress OPEN ACCESS

Article

Embeddings from language models are good learners for single-cell data analysis

Tianyu Liu,[1,2] Tianqi Chen,[2,5] Wangjie Zheng,[2,5] Xiao Luo,[3] Yiqun Chen,[4] and Hongyu Zhao[1,2,6,*]
[1]Interdepartmental Program in Computational Biology & Bioinformatics, Yale University, New Haven, CT 06511, USA
[2]Department of Biostatistics, Yale University, New Haven, CT 06511, USA
[3]Department of Statistics, University of Wisconsin-Madison, Madison, WI 53706, USA
[4]Department of Biostatistics, Johns Hopkins University, Baltimore, MD 21218, USA
[5]These authors contributed equally
[6]Lead contact
*Correspondence: hongyu.zhao@yale.edu
https://doi.org/10.1016/j.patter.2025.101431

THE BIGGER PICTURE Single-cell technologies allow scientists to measure the activity of thousands of genes in individual cells, revealing how tissues develop, age, and respond to disease. Yet, analyzing these massive datasets often demands substantial computing resources and specialized expertise. Our method, single-cell embedding from language models (scELMo), offers an accessible solution by harnessing large language models—the same artificial intelligence systems behind modern chatbots—to interpret biological information. Instead of training large models from scratch, scELMo uses pre-trained language models' knowledge of gene functions and biological concepts to generate detailed numerical representations, or embeddings, of genes. These embeddings capture complex biological information and can be integrated with cellular data to facilitate tasks such as identifying cell types, understanding developmental processes, or exploring disease mechanisms. By uniting advances in computational linguistics and genomics, scELMo transforms language models into engines of biological discovery, expanding access to powerful single-cell analysis tools and accelerating the pace of biomedical insight.

SUMMARY

Foundation models (FMs) have been built to analyze single-cell data with different degrees of success. Here, we present scELMo (single-cell embedding from language models), a method for analyzing single-cell data with the help of large language models (LLMs). LLMs can generate both the description of metadata information and the embeddings for such descriptions. We then combine the embeddings from LLMs with the raw data under the zero-shot learning framework to further extend its function by using the fine-tuning framework to handle different tasks. We demonstrate that scELMo is capable of cell clustering, batch effect correction, and cell-type annotation without training a new model. Moreover, the fine-tuning framework of scELMo can help with more challenging tasks, including *in silico* treatment analysis or modeling perturbation. scELMo has a lighter structure and lower requirements for resources, suggesting a more promising path.

INTRODUCTION

The development of foundation models (FMs) has become increasingly critical across a wide range of domains, including engineering and the sciences.[1–3] Large language models (LLMs) serve as prominent examples of successful FMs. In the field of biology, FMs have been employed for various applications, such as analyzing DNA sequences[4,5] and representing cellular and gene-level information,[6–9] among others. These models have been shown to enhance the performance of diverse downstream tasks. In this work, we concentrate on the intersection of FMs and a specific type of biomedical tabular data: single-cell sequencing data.[10,11] Single-cell sequencing captures molecular profiles at the resolution of individual cells, enabling detailed characterization of cellular activity and identity. Typical features include gene expression levels,[11] protein abundance,[12,13] and DNA methylation states,[14] among others.

A number of pre-trained FMs have been developed to analyze single-cell data using large-scale sequencing datasets compiled from diverse studies. Notable examples include scGPT,[7] Geneformer,[8] and scFoundation,[15] which leverage extensive single-cell transcriptomic data to learn biological patterns. These

© 2025 The Author(s). Published by Elsevier Inc.
This is an open access article under the CC BY license (http://creativecommons.org/licenses/by/4.0/).

models are subsequently applied to downstream tasks by either utilizing cell or gene embeddings as learned representations or by fine-tuning the pre-trained models on newly generated datasets. However, such training protocols require substantial computational resources and storage capacity, and their performance can be highly sensitive to data pre-processing choices and gene selection strategies. Recent benchmarking studies[16,17] have highlighted limitations in the scalability, robustness, and generalizability of current single-cell FMs. In parallel, the developers of GenePT[18] identified a key constraint in relying solely on gene expression profiles for biological inference. To address this, they proposed augmenting gene representations using prompts derived from external biological knowledge. Specifically, GenePT-w extracts gene embeddings from LLMs by feeding them information from the National Center for Biotechnology Information (NCBI),[19] while GenePT-s converts single-cell profiles into ranked gene lists and uses them as prompts to generate cell embeddings.

Although these strategies demonstrate potential for incorporating prior knowledge, they have notable limitations. GenePT-w depends on the structure and completeness of NCBI annotations, which may not fully capture gene function or interactions. GenePT-s is constrained by the intrinsic sparsity of single-cell datasets, which leads to many unexpressed genes that cannot be ranked reliably. Additionally, its scalability is limited by reliance on the OpenAI application programming interface (API), which imposes usage and latency restrictions.[20] GenePT also explores zero-shot transfer learning, but its inability to integrate cell-level metadata, such as cell-type annotations, restricts its applicability to a broader set of tasks. Meanwhile, the emergence of advanced LLMs, such as GPT-3.5,[21] GPT-4,[22] and LLaMA,[23] provides opportunities to integrate large-scale biological knowledge into single-cell analysis. These models have already been applied to scientific knowledge extraction,[24] neural architecture design,[25] and broader applications in computational research.[26,27]

In this manuscript, we explore the ability of using LLMs in a different manner. We generate meaningful text descriptions of cell-level or feature-level metadata as well as embeddings of such descriptions based on LLMs. We assume that the embeddings from LLMs carry biological properties and can be utilized in various downstream applications. Here, we introduce single-cell embedding from language models (scELMo) as a pipeline for analyzing single-cell multi-omics data based on the text description and embeddings directly from LLMs.[28] Using genes as one example, we leverage LLMs to summarize the functional information of a given gene with a suitable prompt and also use the same LLM to extract the embeddings of such a description. We then either incorporate the embeddings directly into the single-cell data by matrix operation or combine the embeddings with other models with fine-tuning targets for various tasks. Different from traditional single-cell FMs, scELMo does not require pre-training with LLM embeddings, and it saves resources to accelerate biological discoveries and insight validation. We demonstrate that scELMo is a simple but effective tool for single-cell data analysis under both the zero-shot learning framework and the fine-tuning framework, supported by comprehensive benchmarking analyses.

METHODS

Problem definition

For a typical single-cell dataset $X^{n \times m}$ after normalization[29] with n cells and m features, our target is to utilize the text description from a mapping function $\mathcal{M}$ for feature-level metadata information $f^{m \times 1}$ and cell-level metadata information $c^{n \times 1}$ to learn the embeddings of cells. Each cell or gene has one corresponding metadata information description, and thus, f and g are vectors. If we define the embedding generation layer of $\mathcal{M}$ as $\mathcal{M}_e$, our cell embeddings ($e_{cells}^{n \times t}$, where t represents the dimension of LLM embeddings) can be represented as

$$e_f^{m \times t} = \mathcal{M}_e(\mathcal{M}(Prompt(f))),$$
$$e_c^{n \times t} = \mathcal{M}_e(\mathcal{M}(Prompt(c))), \text{ and}$$
$$e_{cells}^{n \times t} = AVG(X)e_f + e_c,$$

where *Prompt* is a mapping function that can transfer the name of input data to the prompt space. The prompts can be used as the input for language models. The function *AVG*() represents the method we used to average the embeddings of all genes for each cell. If the mode is the arithmetic average (*aa*), we divide X by m. If the mode is the *wa*, we divide each row of X by the sum of this row. Considering the cell with index i, we can define these two processes as

$$AVG_{aa}(X_i) = \frac{X_i}{m} \text{ and}$$
$$AVG_{wa}(X_i) = \frac{X_i}{sum(X_i)}.$$

Then, we use matrix multiplication to combine the feature embeddings and the expression profile. Our default setting of the mapping function is an LLM. GenePT can be treated as a special case of scELMo, that is, replacing the LLM with a known database and using the *aa* mode. Incorporating the embeddings of cell-level metadata is an optional choice. We intend to investigate if the cell embeddings can offer a better representation than the raw data.

Moreover, with the embeddings of feature-level metadata information and cell-level metadata information, we also consider if incorporating our embeddings with task-specific model $\mathcal{T}$ can improve the performance of $\mathcal{T}$, that is

$$Score(\mathcal{T}(X)) < Score(\mathcal{T}(X, e_f, e_c)),$$

where *Score*() is a metric to evaluate the output of the given model, where a higher value represents a better output. We also may not need to have e_f and e_c for every model.

Method explanation

By default, our framework utilizes embeddings generated from a closed-source OpenAI model. The design of the *AVG* strategy is motivated by prior work[18] and informed by the intrinsic noise characteristics of single-cell RNA sequencing (scRNA-seq) data.[30] By compressing high-dimensional gene expression profiles into a lower-dimensional space, we can jointly encode both quantitative expression levels and functional knowledge of genes.

We hypothesize and empirically demonstrate that these enriched cell embeddings can more effectively represent cellular

identity by integrating external gene function annotations and cell-type-specific information derived from LLMs. As a result, access to raw expression data becomes unnecessary for many downstream analyses. This hypothesis is further supported by a recent systematic evaluation of LLM embeddings in medical machine learning.[31] In our benchmarking, the weighted average (*wa*) mode outperformed other approaches in clustering and batch effect correction tasks. We attribute this to the importance of capturing both individual gene contributions and collective gene group effects when constructing cell embeddings in a zero-shot learning framework. For fine-tuning scenarios, task-specific methods offer more flexible designs to incorporate LLM-derived embeddings as prior knowledge, enabling further performance optimization tailored to specific biological questions.

scELMo under the zero-shot learning framework

To evaluate the performance of e_{cells} under the zero-shot learning framework, we consider three tasks: clustering, batch effect correction, and cell-type annotation based on a k-nearest-neighbor (kNN) classifier. In this section, $\mathcal{T}$ is defined as a kNN classifier.

For clustering and batch effect correction, we directly use e_{cells} as a new representation for X and evaluate e_{cells} for these two tasks. For cell-type annotation based on a kNN classifier, we consider training dataset X_{train} and testing dataset X_{test}, and their corresponding cell embeddings e^{train}_{cells} and e^{test}_{cells}. We use e^{train}_{cells} with its cell types to train a kNN classifier and perform cell-type annotation based on e^{test}_{cells}. Since kNN is based on similarity searching, we treat this method as an ability of zero-shot learning. We follow the settings from GenePT for this classifier and set $k = 10$.

scELMo under the fine-tuning framework

To evaluate the performance of e_f and e_c under the fine-tuning framework, we considered three tasks: cell-type annotation, *in silico* treatment analysis, and perturbation analysis with task-specific models as adaptors.

For the cell-type annotation and *in silico* treatment analysis tasks, we present a light-structured neural network with a contrastive learning[32,33] design. Here, $\mathcal{T}$ is a neural network with rectified linear units (ReLU[34]) as the activation function. Our intuition comes from the requirement for a good representation of cells with different labels and conditions. Therefore, we formalize the loss function of our model as

$$\mathcal{L}_{total} = \mathcal{L}_{classifier} + \lambda \mathcal{L}_{contrastive},$$

where $\mathcal{L}_{classifier}$ represents the classification loss of the model output, as we use cell-type labels for model training, and $\mathcal{L}_{contrastive}$ represents the contrastive learning loss we use to distinguish the representations of cells under different conditions in the latent space. λ is a hyper-parameter, where we set $\lambda = 100$ in this manuscript to assign a larger weight for label-aware clustering. We utilize the embeddings after fine-tuning as the training and testing datasets and use a kNN classifier to annotate the cell types to evaluate the representation we learn based on scELMo.

To analyze the target of *in silico* treatment, we first compute the cosine similarity (CS_{old}) between the average cell embeddings based on the diseased case and the control case. Then, we delete the target gene by setting its expression profile to zero and compute the new embeddings and cosine similarity (CS_{new}). We define the score of our targeted gene g as

$$Score(g) = CS_{new} - CS_{old}.$$

If such a score is larger than 1e−4, we treat the gene we analyze as a candidate for therapeutic targets. This threshold is based on the upper bound of the tiny quantities determined by Numpy[35] for scientific notation representation and the smallest non-zero scale of the y axis in Figures 4A and 4B. We utilize the selected genes to run gene pathway analysis based on Gene Ontology Enrichment Analysis (GOEA)[36–38] and Ingenuity Pathway Analysis (IPA).[39]

For the perturbation analysis task, we consider three different models for the three tasks. Here, $\mathcal{T}$ represents different models corresponding to different perturbation analysis tasks. For the causal factor analysis task and CINEMA-OT, we replace the original input of CINEMA-OT with e_{cells}. We follow the default settings of CINEMA-OT for processing related datasets. For the gene expression prediction task and CPA, we add an additional neural network component to make e_{cells} learnable and combine the output of this component with the latent space from the original CPA. We do not modify the training process of CPA. We follow the default settings of CPA for processing related datasets. For the gene expression prediction task based on perturb-seq-based datasets and GEARS, we add the e_f to the original gene embeddings of GEARS. We do not modify the training process of GEARS. We follow the default settings of GEARS for processing Dixit, Norman, Adamson, and Replogle datasets.

Data pre-processing

We follow the data pre-processing steps from Scanpy[29] for scRNA-seq datasets. For single-cell proteomic datasets, we follow the pre-processing steps from TotalVI[40] and MARIO[41] and do not change the distribution of the original data because of its density.

Metrics

To evaluate the hallucinations of LLM outputs, we consider two metrics: the bilingual evaluation understudy (BLEU)[42] score and the Human-Eval score.[43]

The BLEU score is used to evaluate the similarity between observed string $\hat{y}$ and ground-truth string y based on the *n-grams* strategy. Considering we have a function $C(s,y)$ to generate the number of appearances of s as a substring of y and a set $G_n(\hat{y})$ as the *n-grams* set, the BLEU score is defined as

$$BLEU = \frac{\sum_{s \in G_n(\hat{y})} \min(C(s,\hat{y}), C(s,y))}{\sum_{s \in G_n(\hat{y})} C(s,\hat{y})}.$$

The score is in [0,1], and a higher value means better performance.

The Human-Eval score means we compare the truthfulness between the LLM outputs and references from NCBI and GeneCard databases to assign scores for the string pairs. We assign 1 if the outputs contain the correct information and 0 if the outputs do not contain the correct information. We have one human expert for evaluation. A higher value means better performance.

For the evaluations of clustering and batch effect correction, we utilize the metrics described and implemented by scIB.[44] We compute all the metrics we could in the evaluation process. All of the scores of the metrics in scIB are in [0,1], and a higher value means better performance.

For clustering, we use normalized mutual information (NMI), adjusted Rand index (ARI), and average silhouette width $(\mathrm{ASW})_{label}$ for evaluation. For batch effect correction, we compute ASW_{batch}, principal-component (PC) regression (PCR), graph connectivity (GC), kBET, and integration LISI (iLISI) and average the scores from these metrics to generate S_{batch}. We compute ASW_{label}, NMI, ARI, and cell-type LISI (cLISI) and average the scores from these metrics to generate S_{bio}. Details of these metrics, modified based on Liu et al.[16] and Luecken et al.,[44] are introduced here.

(1) NMI: NMI is a score to evaluate the performance of biological information conservation. We compute this score based on the mutual information between the optimal Leiden clusters and the known cell-type labels and then take the normalization. NMI $\in$ (0,1), and a higher NMI means better performance.

(2) ARI: ARI is a score to evaluate the performance of biological information conservation. ARI is used to evaluate the agreement between optimal Louvain clusters and cell-type labels. ARI $\in$ (0,1), and a higher ARI means better performance.

(3) ASW: We have cell-type ASW (ASW_{cell}) and batch ASW (ASW_{batch}) for this metric. For one cell, ASW calculates the ratio between the inner cluster distance and the intra-cluster distance for this cell. Therefore, higher ASW_{cell} means better biological information conservation, and lower ASW_{batch} means better batch effect correction. To make them consistent, for ASW_{cell}, we take the normalization, that is,

$$ASW_{cell} = \frac{ASW_{cell}^{raw} + 1}{2}.$$

Similarly, for ASW_{batch}, we take the inverse value of the normalized result, that is,

$$ASW_{batch} = 1 - \frac{ASW_{batch}^{raw} + 1}{2}.$$

Both metrics are in (0,1), and a higher score means better model performance.

(4) Local inverse Simpson's index (LISI): LISI is a metric to evaluate whether datasets are well-mixed under batch labels (*iLISI*) or can be discerned with different cell types (*cLISI*). We first compute the kNN list of one cell and count the number of cells that can be extracted from the neighbors before one label is observed twice. Furthermore, we take the normalization for *iLISI* with *B* batches, that is,

$$iLISI = \frac{iLISI^{raw} - 1}{B - 1}.$$

Similarly, for *cLISI* with *C* cell types, we take the inverse value of the normalized result, that is,

$$cLISI = 1 - \frac{cLISI^{raw} - 1}{C - 1}.$$

Both of metrics are in (0,1), and a higher score means better model performance.

(6) GC: GC measures the connectivity of cells in different cell types. If the batch effect is substantially removed, the connectivity of cells of the same cell type from different batches will have a higher connectivity score based on the kNN graph. Therefore, we can compute the GC score for each cell type and take the average. GC score is in (0,1), and a higher score means better batch effect correction performance.

(6) PCR: PCR is a metric to evaluate the performance of batch effect correction. We calculate the R2 for a linear regression of the covariate of interest onto each PC. The variance contribution of the batch effect for all the PCs is based on the sum of the product between the variance of each PC and the R2 of each PC across all PCs. Therefore, the score can be represented as

$$PCR = \sum_{i=1}^{G} \mathrm{Var}\ (C|\mathrm{PC}_i) \times R^2(\mathrm{PC}_i|B),$$

where *G* denotes the number of PCs and *B* denotes the batch information. PCR is in (0,1), and a higher score means better performance.

(7) kBET: The kBET algorithm is used to determine if the label composition of the kNNs of a cell is similar to the expected label composition. For the batch label mixture of cells in the same cell types, the proportion of cells from different batches for the neighbors of one cell should match the global-level distribution. The kBET score $\in$ (0,1), and a higher score means better batch effect correction performance.

For the evaluations of cell-type annotation, we use Scikit-learn[45] to calculate accuracy, precision, recall, and F1 score by comparing the predicted cell-type labels and ground-truth cell-type labels. All of the metrics are in [0,1], and a higher value means better performance.

For the evaluations of *in silico* treatment analysis, we use Scipy[46] to compute the cosine similarity between the mean cell embeddings from the control case and the mean cell embeddings from the diseased case. The definition of the score here is described in the methods.

For the evaluations of perturbation analysis, we have three different tasks with different metrics. For the causal factor analysis task, the metrics are the same as those we use in the batch effect correction task. For the gene expression prediction task based on CPA, we use the R2 score as a metric to evaluate the performance for regression. The R2 score is defined as

$$R2 = 1 - \frac{SS_{res}}{SS_{tot}} = 1 - \frac{\sum_i (y_i - f_i)^2}{\sum_i (y_i - \bar{y})^2},$$

where y_i represents the ground-truth gene expression level, f_i represents the predicted gene expression level, and $\bar{y}$ represents the average expression levels of the given gene. A higher

average R2 score and a lower variance mean better performance.

For the evaluation of gene expression prediction tasks based on GEARS, we use the Pearson correlation coefficient (PCC) and mean squared error (MSE) as metrics. We define PCC as

$$PCC = \frac{1}{m}\sum_{i}^{m}\frac{\mathrm{cov}(y_i, f_i)}{\sigma_{y_i}\sigma_{f_i}},$$

where m represents the number of used genes, cov represents the covariance, and σ represents the standard deviation. ρ is in [0,1], and a higher value means better performance. We can also define MSE as

$$MSE = \frac{1}{mn}\sum_{i}^{m}\sum_{j}^{n}\left(y_{ij} - f_{ij}\right)^2,$$

where y_{ij} represents the ground-truth gene expression level of gene i in cell j and f_{ij} represents the predicted gene expression level of gene i in cell j. A lower MSE score means better performance.

We consider computing these two metrics for both the all-genes case and the top 20 differentially expressed genes (DEGs) case.

Explanations of baseline models

For tasks related to description generation, we consider MetaPrompt[47] and chain of thought (COT)[47] as baseline models for prompt engineering. MetaPrompt introduces a system prompt for LLMs and generates the outputs conditioned on the context in the system prompt. COT allows LLMs to obtain complex reasoning capabilities by allowing models to address the problem with intermediate steps.

For tasks related to clustering, we consider principal-component analysis (PCA) (raw),[45] GenePT,[18] SC3,[48] and scVI[49] as baseline models. PCA is widely used for dimension reduction of single-cell data. The principles of GenePT are summarized in the introduction. SC3 utilizes consensus clustering for analyzing scRNA-seq data, which is based on aggregating the k-means results after PCA transformation to generate the consensus, and then performs the clustering based on consensus. scVI utilizes a generative model to learn the embeddings in the latent space of scRNA-seq data.

For tasks related to batch effect correction, we consider PCA (raw), GenePT, MARIO,[41] Harmony,[50] and MNN[51] as baseline models. MARIO considers both the shared features and distinct features for proteomic data and performs paired matching for cells from different batches to generate integrated results in the latent space. Harmony assigns labels for different cells with a soft-clustering method, computes the centroids for different clusters, and updates cell embeddings based on the soft cluster membership. MNN utilizes mutual nearest neighbors to learn the relationships for cells in different batches and updates the cell embeddings based on the relationships.

For tasks related to cell-type annotation, we consider GPT-2,[52] GPT-4,[22] scGPT,[7] Geneformer,[8] GPTCelltype,[53] multi-layer perceptron (MLP), and PCA for evaluation. To evaluate GPT-2 and GPT-4, we transfer the cell information into a sentence based on the rank of genes for each cell and treat this task as a question-answer task. scGPT is a pre-training-based FM for multiple tasks in single-cell research. The authors utilized multi-layer transformers to construct the model architecture and pre-trained scGPT based on large-scale scRNA-seq datasets. They fine-tuned scGPT to address problems in downstream applications such as cell-type annotation. Geneformer is also a model based on pre-training. The authors utilized BERT[54] as a base model and pre-trained BERT from the sketch using scRNA-seq data transferred into sentences. They also fine-tuned Geneformer to address problems in downstream applications, including cell-type annotation, *in silico* treatment analysis, etc. GPTCelltype utilizes GPT-4 to extract markers of cell types to annotate cell clusters. To evaluate this model, we unify the model outputs and ground-truth cell-type labels. MLP means we fit an MLP based on gene expression profiles and cell types. We extract the embeddings from these two models and PCs based on both the zero-shot learning framework and the fine-tuning framework and utilize kNN to perform classification based on the embeddings.

For tasks of perturbation analysis, we consider CINEMA-OT,[55] CPA,[56] and GEARS[57] for modification and evaluation. We included gene embeddings from GPT-3.5, NCBI (GenePT), and scGPT as inputs. CINEMA-OT is a method for separating confounding signals or causal factors from perturbations at the single-cell level. The first step is to initialize the expected matrix rank based on biwhitening.[58] The second step is to separate confounder signals and treatment-associated signals based on independent component analysis (ICA).[45] The last step is to match the cells based on entropy regularized optimal transport.[59] We analyze the confounder embeddings for CINEMA-OT and follow the benchmarking pipeline mentioned in the original paper. CPA is a method for modeling the gene expression levels of scRNA-seq data with perturbations. CPA treats perturbations of cells as covariates and encodes these covariates as embeddings into the training process of a conditional variational autoencoder (CVAE). CPA can also be used to predict the gene expression levels of out-of-distribution (OOD) samples. GEARS is a method for predicting gene expression levels of perturb-seq-based datasets. It models the perturbations of genes based on knowledge graphs and gene embeddings, thus utilizing graph neural networks (GNNs) and MLPs to predict the gene expression levels under gene-level perturbation.

RESULTS

Overview of scELMo

Embeddings generated by LLMs are highly effective at preserving the contextual and semantic structure of their input.[60–62] This property makes them compelling priors for integrating enriched biological knowledge into downstream analyses. The core concept of scELMo is to map cellular information from the sequencing data space into an LLM-derived embedding space. This transformation is accomplished by incorporating metadata at either the feature level (e.g., genes and proteins) or the cell level (e.g., cell types and cell states). For feature-level integration, we extract functional information for each feature either from curated databases such as NCBI or by prompting an LLM to summarize relevant biological knowledge. The textual summaries are then passed through the LLM's embedding layers to obtain feature representations in the embedding space. In

this study, we use GPT-3.5 to both summarize feature functions and generate embeddings, based on its superior performance in our benchmarking analyses.

Once embeddings are extracted from LLMs and transcriptomic profiles are obtained from single-cell data, they can be integrated under a zero-shot learning framework to generate enriched cell embeddings. These integrated embeddings—constructed via matrix operations that combine LLM-derived feature embeddings with cellular expression profiles—can be directly used for tasks such as cell clustering or batch effect correction. Given recent evidence highlighting the benefits of incorporating biological knowledge into single-cell modeling,[7,63,64] we also explore a fine-tuning framework. In this setup, LLM-derived embeddings serve as enhanced biological priors that are fed into task-specific models, which are subsequently retrained for downstream applications. This approach provides greater flexibility and allows for improved performance on task-specific objectives by leveraging the contextual richness of LLM-generated embeddings. Figure 1 provides an overview of scELMo under both the zero-shot and fine-tuning paradigms. Detailed implementations for each framework are described in the methods.

Evaluation of hallucinations in LLMs

The first step in our pipeline is to select an appropriate LLM for generating both textual descriptions and corresponding embeddings. An ideal LLM for this application should minimize hallucinations,[65] i.e., avoid generating fabricated or incorrect biological information based on the provided feature or cell metadata. To this end, we evaluated several candidate models, including GPT-2,[52] GPT-3.5, GPT-4, LLaMA-2 (70B), Mistral,[66] BioGPT,[67] Claude 2,[68] and Bard (PaLM 2).[69] After considering factors such as token diversity, accuracy, and accessibility to embedding layers, we selected GPT-3.5 as the default model for generating embeddings. To benchmark the quality of its textual outputs, we randomly sampled 20 proteins from a known list (~200 total)[70] and 20 genes from the NCBI database (out of ~30,000 genes) and prompted each of the listed LLMs to generate functional descriptions for these features. We assessed the correctness of the outputs by comparing them to curated references from GeneCards[71] and NCBI, using both the BLEU[42] score and human evaluation.[43] BLEU measures the degree of *n-gram* overlap between the LLM outputs and human-written references, providing an estimate of textual similarity and accuracy. The evaluation results for genes and proteins are shown in Figure 2A, with corresponding query times per model reported in Figure 2B. Additional results for cell-type descriptions are provided in Figure S1A.

Our evaluation showed that GPT-3.5 and GPT-4 produced the most accurate functional descriptions of biological features. However, compared to GPT-3.5, GPT-4 incurred significantly longer response times and occasionally failed to comply with the required prompt formatting. To further assess the impact of prompt design, we explored additional prompting strategies, including MetaPrompt[47] and COT[72] prompting. Neither approach improved BLEU scores relative to the default prompt, as shown in Figure S1B. In fact, COT and MetaPrompt led to lower Human-Eval scores compared to the original format, indicating reduced alignment with expert-curated references. To expand our analysis, we scaled the evaluation to 100 randomly selected genes and computed BLEU scores by comparing LLM-generated descriptions against NCBI entries. GPT-3.5 continued to outperform other models, yielding the highest BLEU score with low variance, followed by Claude (Figure S2). Factoring in both description accuracy and runtime performance, we selected GPT-3.5 as the default LLM for generating feature descriptions. The results shown in Figure S1A further confirm GPT-3.5's capability in generating accurate cell-type descriptions.

We also assessed the stability and biological specificity of GPT-3.5-derived embeddings. As shown in Figure S3A, the cosine similarity between embeddings generated across multiple GPT-3.5 responses for the same gene exceeded 0.9, indicating high consistency. By contrast, pairwise cosine similarity between embeddings from distinct genes was substantially lower (range: 0.74–0.84), demonstrating the ability of the embeddings to differentiate functionally distinct genes (Figure S3B). Additional evaluation of embedding variability using different random seeds is presented in the discussion. To further validate the functional relevance of the embeddings, we applied a kNN classifier (k = 10) using Geneformer's functional gene annotations.[8] A train-test split (80%-20%) yielded an accuracy of 0.931, underscoring the informative nature of the embeddings. Figures S4A and S4B illustrate functional clustering patterns, where genes with similar roles are embedded proximally.

To evaluate the biological coherence of these clusters, we performed GOEA[36–38] and IPA[39] on the top 10 protein-coding gene clusters, ranked by size. Across all clusters, top enriched pathways (ranked by $-log$(adjusted p value)) involved essential biological processes, including metabolism, transport, and genetic regulation (Figures S5 and S9). These results demonstrate that GPT-3.5-derived embeddings preserve gene functional information and support downstream analyses. Additional text analyses comparing NCBI and LLM-generated gene descriptions are detailed in Note S1, with raw outputs summarized in Data S1. We also demonstrate the applicability of LLM-derived embeddings to single-cell and spatial transcriptomics datasets across different species, highlighting potential for cross-species analyses in Note S2.

scELMo for clustering and batch effect correction

In this section, we investigated the contribution of feature embeddings from scELMo for cell-level tasks, including clustering and batch effect correction.

Clustering provides an effective means of evaluating whether feature embeddings encode biologically relevant information. To assess this, we integrated gene or protein embeddings into single-cell sequencing data and performed clustering based on the resulting cell embeddings. We quantified clustering performance using three widely adopted metrics in single-cell analysis: NMI, ARI, and ASW.[44] Unless otherwise noted, GenePT refers to the GenePT-w variant in the following analyses. Because we generated gene embeddings using both NCBI and Ensembl[73] gene identifiers, our embedding set contained a larger number of genes than that used by GenePT. In GenePT, the default strategy for integrating feature embeddings into cells is a naive *aa*, where cell embeddings are obtained by computing the dot product between the gene expression vector and the matrix of gene embeddings generated by GPT-3.5. However, this approach

CellPress
OPEN ACCESS

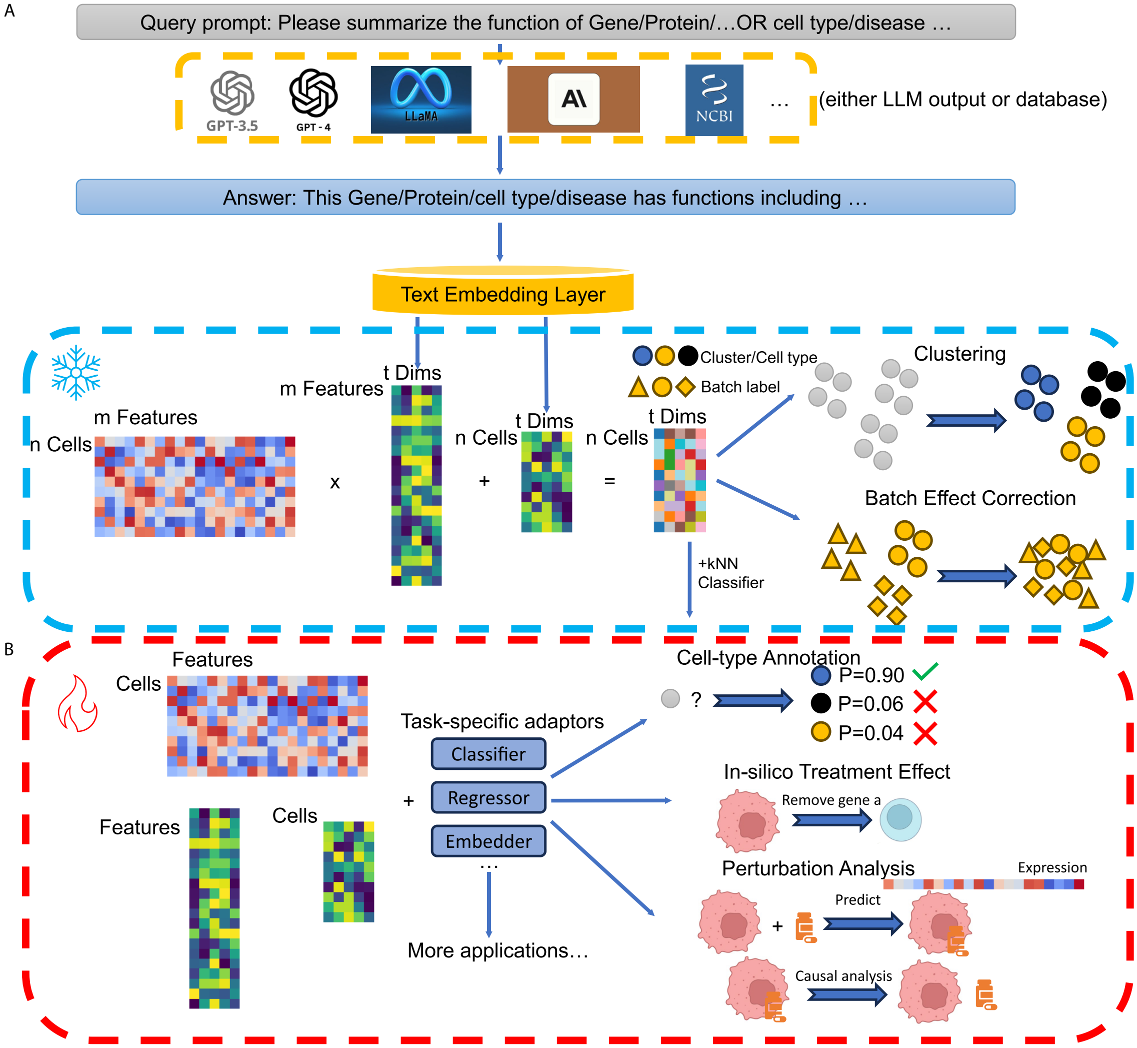

Figure 1. Workflow of scELMo

(A) Zero-shot learning (denoted as ice) framework of scELMo. We extract the text description of metadata by using either databases or LLMs. The prompts are adjustable. We use GPT-3.5 to generate the embeddings of the text descriptions as the embeddings of features (including genes, peaks, etc., which are biological features from cell profiles) or cell states. We then aggregate these embeddings with single-cell profiles to generate cell embeddings.

(B) Fine-tuning (denoted as fire) framework of scELMo. We combine embeddings of metadata and single-cell profiles with task-specific adaptors and train the adaptors to address downstream applications.

disregards differences in the magnitude of log-normalized gene expression across cells, even though gene expression levels are known to significantly influence cellular function.[74,75]

To address this limitation, we propose an alternative approach that incorporates gene expression values as weights, computing a *wa* for each cell. This approach better captures the functional contributions of genes with varying expression levels. Both the naive and *wa* strategies were implemented for generating cell embeddings, with further details provided in the methods. Using the *wa* mode was better than using the *aa* mode for cell clustering under different metrics, as shown in Figure 2C. Meanwhile, the *wa* mode has better performances in three out of four datasets compared with the task-specific methods SC3[48] and scVI.[49] Moreover, using embeddings from scELMo also improved the clustering performance compared with the embeddings from GenePT, advocating the use of LLMs as a tool for summarizing scientific concepts. However, various approaches to combining the embeddings from GenePT and GPT-3.5 (e.g., GPT-3.5 + GenePT *wa* means sum by genes and GPT-3.5 || GenePT *wa* means concatenation by genes) did not improve the scores compared with the individual setting. The average ranks of all methods across different datasets are summarized in Figure S6A, with the GPT-3.5 *wa* mode having the lowest rank. Finally, if we combined cell embeddings with the embeddings of cell-type information from GPT-3.5, we could get scores close to one, suggesting that the cell-type embeddings contained meaningful cell-type information. Note S3 summarizes factors that could affect clustering performance.

For the batch effect correction task, we first focused on single-cell proteomic data—specifically CITE-seq[13] and CyTOF[12]

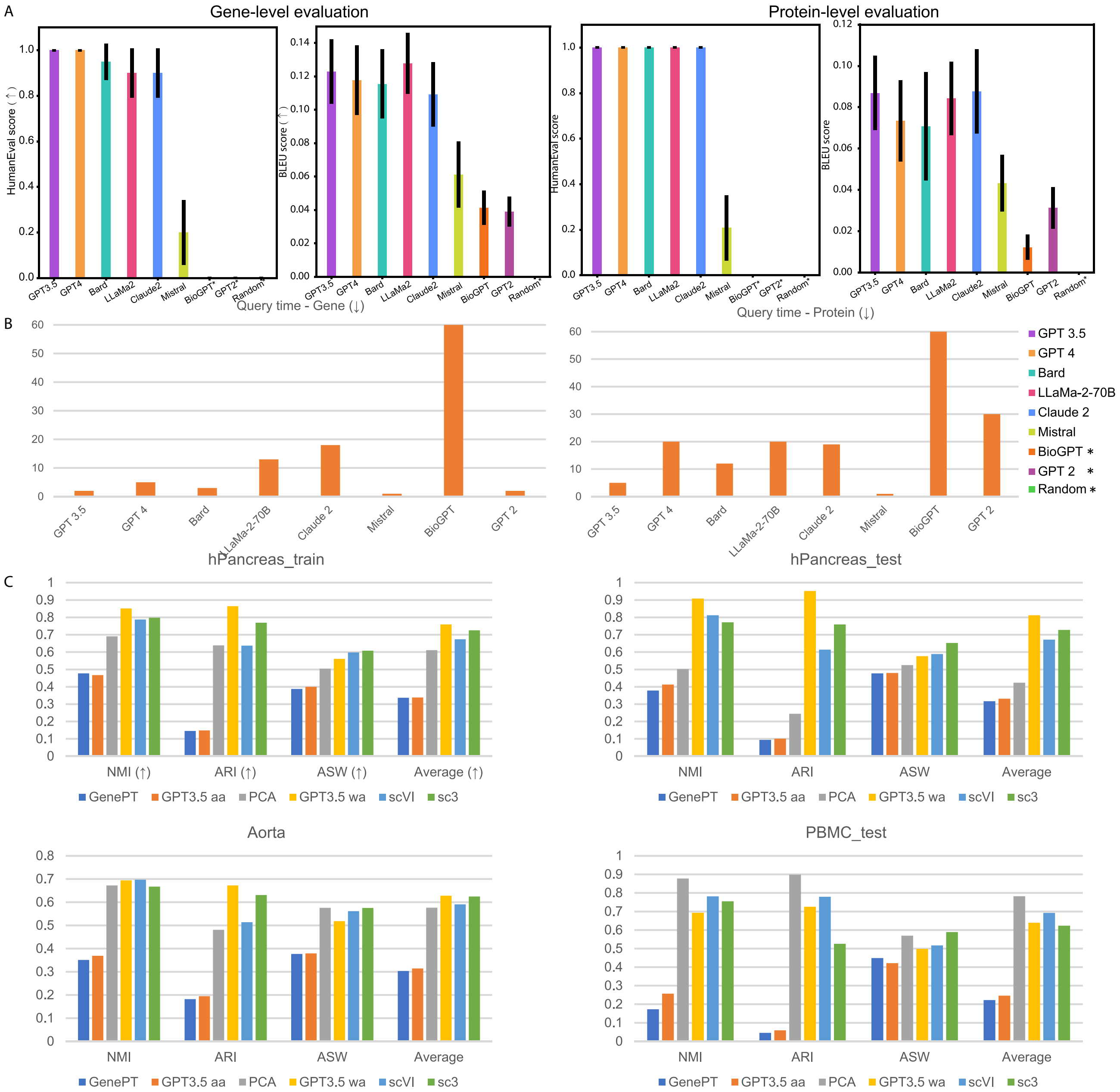

Figure 2. Evaluations of the outputs of LLMs and the clustering performance

(A) Metrics for evaluating meaningful outputs of biological features across different LLMs. The left image represents the BLEU score and Human-Eval score of genes, while the right image represents the BLEU score and the Human-Eval score of proteins.

(B) Average query time for each LLM. The left image represents the query time of genes, and the right image represents the query time of proteins.

(C) Evaluations of the clustering performance based on major methods. Different images represent the results of different datasets.

* represents that the selected method has a zero Human-Eval score.

datasets—since GenePT had already assessed performance on small-scale scRNA-seq data. To ensure fair benchmarking, we adopted a projection-based strategy, aligning datasets from different batches into a shared embedding space without further training. Two scenarios were considered: batch correction for datasets generated using (1) the same experimental protocol and (2) distinct protocols. We compared the batch correction performance of embeddings produced by scELMo and GenePT. Evaluation was conducted using scIB metrics,[44] separating the contribution to batch effect removal (S_{batch}) from the preservation of biological variation (S_{bio}). A detailed description of these metrics is provided in the methods. Additionally, we benchmarked against task-specific batch correction methods, including MARIO,[41] Harmony,[50] and MNN.[51]

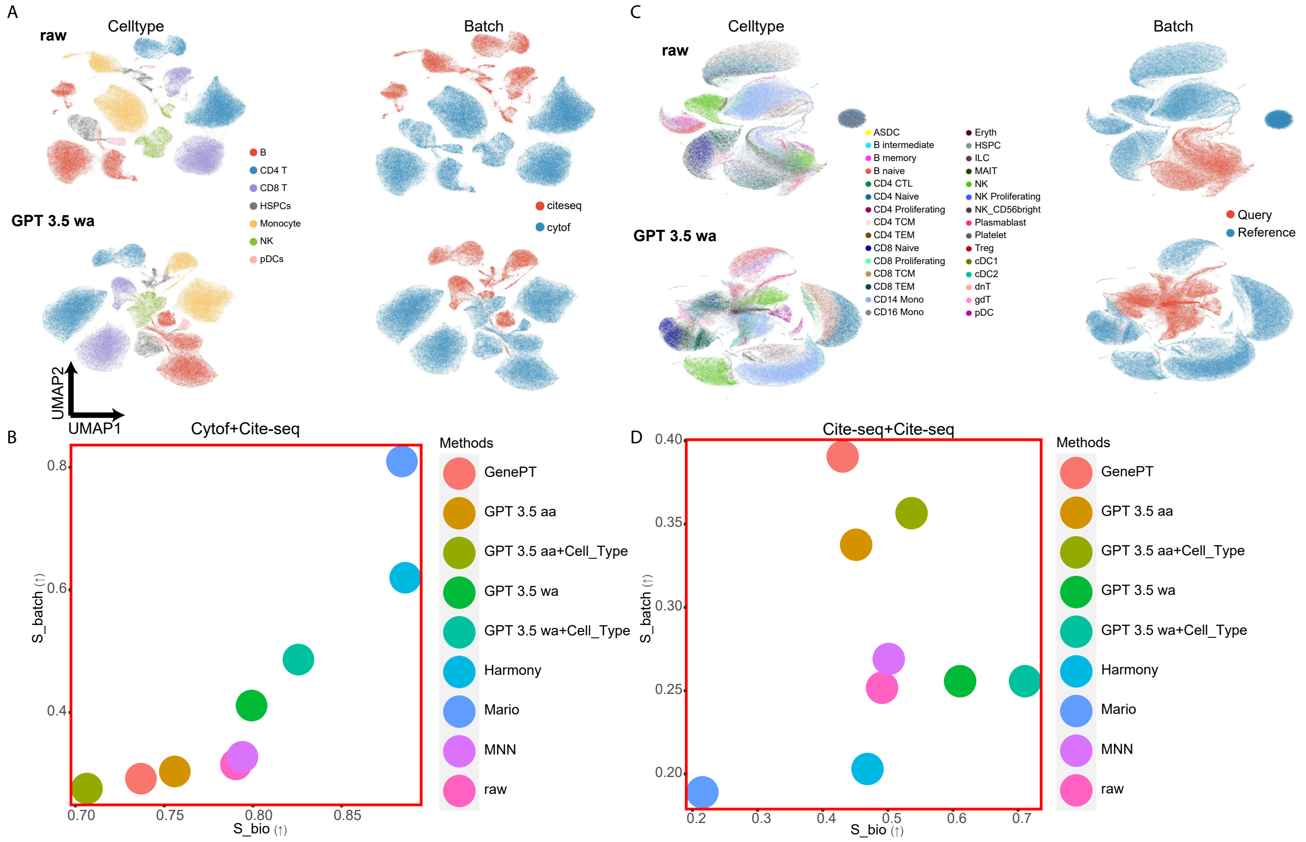

Figure 3. Results of batch effect correction for single-cell proteomic data

(A) Uniform manifold approximation and projections (UMAPs)[76] for the cell-type information (left) and batch information (right) of CITE-seq-based datasets. The top image represents the raw data. The bottom image represents the cell embeddings from GPT-3.5 *wa* mode.

(B) Evaluations of the batch effect correction for CITE-seq-based datasets across different methods.

(C) UMAPs for the cell-type information (left) and batch information (right) of CITE-seq-based dataset and CyTOF-based dataset. The top image represents the raw data. The bottom image represents the cell embeddings from GPT-3.5 *wa* mode.

(D) Evaluations of the batch effect correction for CITE-seq-based dataset and CyTOF-based dataset across different methods.

Results for two CITE-seq datasets are shown in Figures 3A and 3B, and the results for a combined CITE-seq and CyTOF dataset are shown in Figures 3C and 3D. In both scenarios, the naive *aa* approach failed to improve batch correction performance for scELMo, a trend consistent across task-specific methods. Although MARIO exhibited strong performance in integrating CITE-seq and CyTOF datasets, it showed no advantage when integrating two CITE-seq datasets. MNN underperformed compared to both *aa* and *wa* approaches in all cases.

Notably, the *aa* method reduced both S_{batch} and S_{bio} in the CyTOF+CITE-seq case, suggesting an increased batch effect and a diminished biological signal. In contrast, the *wa* approach consistently reduced batch effects while maintaining biological variation. Incorporating cell-type embeddings under the *aa* mode did not improve either metric. However, combining cell-type embeddings with cell embeddings under the *wa* mode enhanced S_{bio} in both scenarios, underscoring the importance of a robust base embedding space for effectively leveraging cell-state information. We extended our evaluation to atlas-scale scRNA-seq datasets,[77–79] and as shown in Figure S7, scELMo outperformed GenePT in integrating large-scale transcriptomic datasets. A summary of our multi-omics data integration analysis[80] is provided in Note S4.

scELMo for cell-type annotation

Cell-type annotation is a fundamental task in single-cell data analysis.[81] In this work, we evaluated the performance of scELMo for cell-type classification under two different frameworks: zero-shot learning and fine-tuning. Under the zero-shot learning framework, we incorporated gene embeddings into both the training and testing datasets to generate cell embeddings and then used the kNN classifier implemented in GenePT to assign cell types in the testing set. However, this approach failed to generalize effectively when the training data originated from heterogeneous sources or displayed pronounced batch effects, as evidenced in the peripheral blood mononuclear cell (PBMC) dataset results shown in Table 1. To address this limitation, we designed a simple neural-network-based classifier enhanced with contrastive learning,[32] similar in spirit to adaptors in natural language processing (NLP) applications.[28] The architecture and training rationale are provided in Figure S8 and the accompanying methods description. The

Table 1. Scores of cell-type annotation task under different settings

hPancreas[82–85]	Accuracy (↑)	Precision (↑)	Recall (↑)	F1
Zero-shot settings				
GPT-2 query	0.000	0.000	0.000	0.000
GPT-4 query	0.000	0.000	0.000	0.000
scGPT (z)	0.770	0.610	0.560	0.550
Geneformer (z)	0.500	0.250	0.340	0.270
GenePT-w	0.940	0.720	0.630	0.650
GenePT-s	0.890	0.650	0.530	0.560
GPTCelltype	0.501	0.386	0.415	0.366
PCA	0.633	0.369	0.382	0.357
GPT3.5 *aa*	0.933	0.702	0.614	0.629
GPT3.5 *wa*	0.933	0.702	0.613	0.629
Fine-tuning settings				
scGPT	**0.970**	0.742	**0.758**	**0.741**
Geneformer	0.321	0.105	0.094	0.086
scELMo+GenePT	0.963	**0.790**	0.662	0.680
scELMo+random emb	0.968	0.683	0.693	0.680
scELMo+GPT-3.5	**0.970**	0.769	0.674	0.687
Aorta[86]	Accuracy	Precision	Recall	F1
Zero-shot settings				
GPT-2 query	0.000	0.000	0.000	0.000
GPT-4 query	0.000	0.000	0.000	0.000
scGPT (z)	0.938	0.921	0.894	0.901
Geneformer (z)	0.860	0.700	0.600	0.620
GenePT-w	0.870	0.910	0.680	0.720
GenePT-s	0.860	0.700	0.600	0.620
GPTCelltype	0.340	0.254	0.261	0.228
PCA	0.929	0.923	0.911	0.910
GPT3.5 *aa*	0.877	0.867	0.678	0.719
GPT3.5 *wa*	0.877	0.867	0.677	0.718
Fine-tuning settings				
scGPT	**0.962**	0.938	0.939	0.937
Geneformer	0.340	0.087	0.092	0.080
scELMo+GenePT	0.958	**0.952**	**0.940**	**0.946**
scELMo+random emb	0.951	0.913	0.874	0.882
scELMo+GPT-3.5	0.957	0.950	0.936	0.942
PBMC[87–90]	Accuracy	Precision	Recall	F1
Zero-shot settings				
GPT-2 query	0.000	0.000	0.000	0.000
GPT-4 query	0.000	0.000	0.000	0.000
scGPT (z)	0.915	0.781	0.803	0.789
Geneformer (z)	0.126	0.128	0.130	0.095
GenePT-w	0.286	0.607	0.270	0.315
GenePT-s	–	–	–	–
GPTCelltype	0.501	0.387	0.415	0.366
PCA	0.436	0.392	0.278	0.285
GPT3.5 *aa*	0.190	0.564	0.181	0.230
GPT3.5 *wa*	0.190	0.562	0.181	0.231

Table 1. *Continued*

hPancreas[82–85]	Accuracy (↑)	Precision (↑)	Recall (↑)	F1
Fine-tuning settings				
scGPT	0.933	0.798	0.822	0.807
Geneformer	0.235	0.146	0.147	0.131
scELMo+GenePT	0.919	0.785	0.823	0.801
scELMo+random emb	0.903	0.854	0.826	0.835
scELMo+GPT-3.5	**0.955**	**0.940**	**0.929**	**0.934**

Parts of the results are directly extracted from GenePT. Here, PCA represents principal-component analysis, and scELMo+random emb represents fine-tuning scELMo with random numbers as meaningless gene embeddings. Average ranks of all methods across datasets are summarized in Figure S6B. The highest score of each metric for each dataset is set in bold.

model was trained on combined gene expression profiles and LLM-derived gene embeddings—thus integrating both quantitative and semantic information. We evaluated this model across three datasets from distinct tissues. For the hPancreas and PBMC datasets, all but one batch were used for training, with the held-out batch used for testing. For the Aorta dataset, we adopted the 80/20 train-test split used in the original GenePT study.[18] These experiments enabled us to assess the effectiveness of LLM-enriched embeddings for cell-type annotation across varying tissue types and dataset complexities.

Table 1 demonstrates that GPT-3.5-derived embeddings exhibit strong zero-shot learning capabilities for cell-type annotation in both the hPancreas and PBMC datasets. Notably, scELMo outperformed GPTCelltype,[53] a method that relies on LLM-extracted marker genes for annotation. We attribute this improvement to GPTCelltype's dependency on pre-clustering and pseudo-labeling steps prior to LLM-based annotation, which can reduce marker quality—particularly in complex datasets with subtle or overlapping cell-type signatures.

In contrast, scELMo enhances cell representation by directly integrating gene-level LLM embeddings, leading to improved accuracy in cell-level tasks such as annotation. We also found that representing cells using only ranked gene expression or embeddings from GPT-2 or GPT-4 resulted in substantially inferior performance. These observations suggest that while zero-shot cell-type annotation is feasible using LLM-derived gene embeddings, its success is contingent upon the level of batch heterogeneity in the dataset. For example, in the PBMC dataset containing samples from multiple sources, the zero-shot performance lagged behind the results obtained via fine-tuning. Importantly, fine-tuning scELMo's adaptor with GPT-3.5 or GenePT embeddings yielded performance comparable to state-of-the-art FMs such as scGPT[7] and Geneformer.[8] However, both scGPT and Geneformer require substantially higher computational resources (e.g., NVIDIA A100 GPUs) and longer fine-tuning times,[16] as shown in Figures S10A and S10B. Across all tested datasets, the fine-tuning framework of scELMo consistently outperformed the zero-shot learning framework, with the added benefit of adapting to embeddings from various sources.

Overall, our results underscore that fine-tuning an adaptor model using gene embeddings from LLMs enables accurate, resource-efficient cell-type annotation, making scELMo both practical and scalable for diverse single-cell datasets.

scELMo for *in silico* treatment analysis

Computational methods for therapeutic target discovery and drug development have garnered significant interest.[91–93] In this section, we extend the functionality of scELMo by combining its adaptor-based cell-type annotation model with gene embeddings from GPT-3.5 or NCBI to model human diseases and identify candidate therapeutic targets. This represents a novel direction not previously explored in GenePT. Inspired by Geneformer,[8] we fine-tuned our adaptor model on a cell-condition classification task and used the resulting cell embeddings to reveal potential molecular targets. A train-validation split was employed to select the optimal adaptor configuration, and the model was then evaluated by systematically removing DEGs between disease and control conditions to observe changes in cell embedding profiles. A detailed discussion on the role of fine-tuning in this context is provided in Note S5. As shown in Figure 4C, embeddings derived from GPT-3.5 consistently yielded the most accurate results. Specifically, when a candidate therapeutic target was removed, cell embeddings under the disease condition became more similar to those under the control condition—an outcome quantified using cosine similarity between disease-specific embeddings and the mean embedding of control cells. This direct embedding-based metric enables *in silico* prioritization of therapeutic targets. We further validated the identified targets using GOEA and literature review, with the findings summarized in Data S2. Overall, these results illustrate how scELMo can facilitate computational disease modeling and target discovery by leveraging LLM-derived gene embeddings and fine-tuned adaptation mechanisms.

We first investigated hypertrophic and dilated cardiomyopathy (HCM and DCM, respectively)[94] using a scRNA-seq dataset derived from human heart tissue.[95] By simulating *in silico* gene deletions under disease conditions, we identified genes whose removal led to significant shifts in cell embeddings toward the non-failing (NF) control state. These changes were quantified by comparing cosine similarity values before and after *in silico* deletion. The results, summarized in Figure 4A, show that scELMo identified two putative therapeutic targets for DCM and four for HCM. Notably, gene embeddings derived from both GPT-3.5 and NCBI sources yielded consistent predictions. The *in silico* silencing of these genes resulted in embedding-level shifts resembling cells under control conditions, suggesting their potential as therapeutic targets. Literature review supports the involvement of several identified genes in cardiomyopathy pathology, including ANKRD1,[96] EXT1,[97] NPPB,[98] and TTTY10.[99] In addition, GSN has been validated as a therapeutic target using CRISPR-based approaches[100] and was also recovered by our model, consistent with prior findings.[8] We further validated the functional relevance of the predicted targets via GOEA and IPA. Figures S11A and S11B display enriched pathways—each with at least ten significantly enriched terms ranked by $-log$(adjusted p value)—while the IPA results are summarized in Figure S11C. These analyses highlight key roles of the selected genes in cardiac muscle contraction, ion transport, and other pathways central to heart physiology, providing biological plausibility for their role in disease modulation. Together, these findings demonstrate the potential of scELMo for *in silico* therapeutic target discovery by integrating LLM-derived gene embeddings with condition-specific cellular embeddings.

Next, we investigated ascending aortic aneurysm,[101] which comprises three distinct disease states. For this analysis, we used the scRNA-seq Aorta dataset and applied the same *in silico* gene deletion strategy as described above. The resulting changes in cosine similarity between disease and control embeddings are summarized in Figure 4B. Using scELMo, we identified six candidate therapeutic genes for the ascending-only state, two for the ascending-to-descending state, and three for the ascending-with-root-involvement state. These predictions are visualized in Figure 4B. Notably, MT-ATP6 was highlighted as a potential target across states. This mitochondrial gene has previously been linked to variable rates of cell death[102] and neurodegeneration,[103] suggesting that cells in distinct aneurysm states may exhibit differing susceptibilities to degeneration or stress. We further investigated the biological relevance of the selected genes using GOEA, with the results shown in Figures S11C–S11E, and IPA, summarized in Figure S12B. Consistent across analyses, the identified genes were significantly enriched in pathways associated with immune responses. These findings highlight the potential mechanistic links between immune dysregulation and aneurysm progression and underscore the value of scELMo in uncovering gene-disease relationships with clinical relevance.

scELMo for perturbation analysis

Analyzing the effects of perturbations on cell states using single-cell data is a critical task in modern biology. Since gene embeddings generated by LLMs encode functional information about potential perturbation targets, we sought to investigate how scELMo could be applied to perturbation modeling and analysis. To this end, we focused on three distinct tasks spanning both chemical perturbations[104] and gene-level perturbations (e.g., CRISPR-based perturb-seq).[105] Our strategy was to leverage cell or gene embeddings generated under the zero-shot learning framework of scELMo, either as replacements for or complements to the original inputs of existing perturbation modeling methods. We evaluated three representative tasks: (1) causal factor analysis using CINEMA-OT,[55] (2) gene expression prediction under chemical perturbation using CPA,[56] and (3) gene expression prediction under gene-level perturbation using GEARS.[57] In each case, we assessed the contribution of scELMo by comparing performance using the original model inputs vs. inputs augmented or replaced with scELMo-derived embeddings. Evaluation metrics were consistent with those defined by the respective methods.

CINEMA-OT is a causal learning framework based on optimal transport that separates perturbation effects from intrinsic cell-state effects in scRNA-seq datasets. To evaluate the contribution of our embeddings, we replaced CINEMA-OT's default input (PCA features) with cell embeddings from scELMo. First, in the intrinsic cell-state (confounder) space where perturbation effects are removed, embeddings from the same cell type should not differ by perturbation cases. Using our cell embeddings as input improved CINEMA-OT's performance compared with PCA, leading to reduced batch effects for cells of the same type, as shown in Figure 5A. Furthermore, in this setting, GenePT gene embeddings outperformed gene embeddings derived from GPT-3.5. Second, we examined whether gene embeddings alone are sufficient for causal factor analysis across different

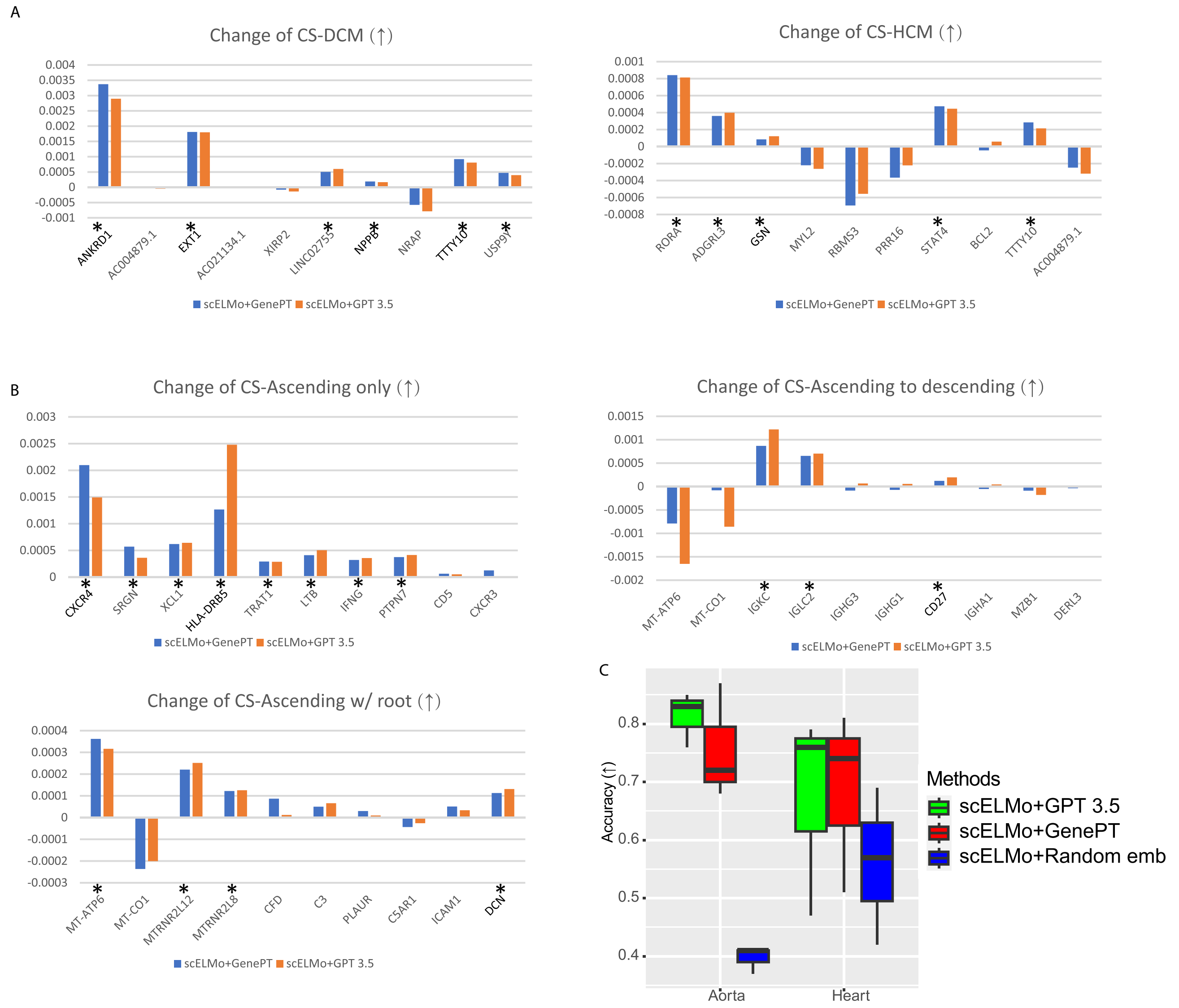

Figure 4. Results of *in silico* treatment analysis

A gene is treated as a potential therapeutic target if the change of cosine similarity (CS) by removing the gene is larger than 1e−4. The reason for setting such a threshold is discussed in the methods. We chose the top 10 DEGs as candidates.

(A) The change of CS by removing different genes in the expression space for hypertrophic (HCM) or dilated cardiomyopathy (DCM) states.

(B) The change of CS by removing different genes in the expression space for ascending aortic aneurysm (ascending only, ascending to descending, and ascending with root).

(C) The accuracy of disease-state annotation under different settings of scELMo. We highlighted the genes detected by both GenePT and scELMo using asterisks (*) and marked the genes that were discovered by previous research as therapeutic targets using bold type.

cell types. As shown in Figure 5B, relying solely on gene embeddings did not significantly improve the separation of perturbation effects and intrinsic cell states. In contrast, when we integrated both gene-level and cell-type embeddings, the modified CINEMA-OT achieved substantially better separation across perturbation cases. This improvement was supported by Wilcoxon rank sum tests comparing GenePT (default) and scELMo embeddings, yielding significant differences in two datasets ($p = 0.031$ for both cases).

Moreover, Figures S13A and S13B show that the visualization could also be improved by using the updated embeddings as model input. Figures S13C and S13D show that incorporating the cell embeddings into CINEMA-OT did not affect the analysis of the gene synergy effect, and the difference between the synergy of monocytes and the synergy of other cell types was also obvious. For the causal factor analysis task, using the *wa* mode did not improve the score. One possible reason is that CINEMA-OT can learn the best representation of cell embeddings with a good start for optimization, so the *aa* mode is adequate. Therefore, scELMo can improve the performance of CINEMA-OT on the causal factor analysis task by offering another candidate of input data.

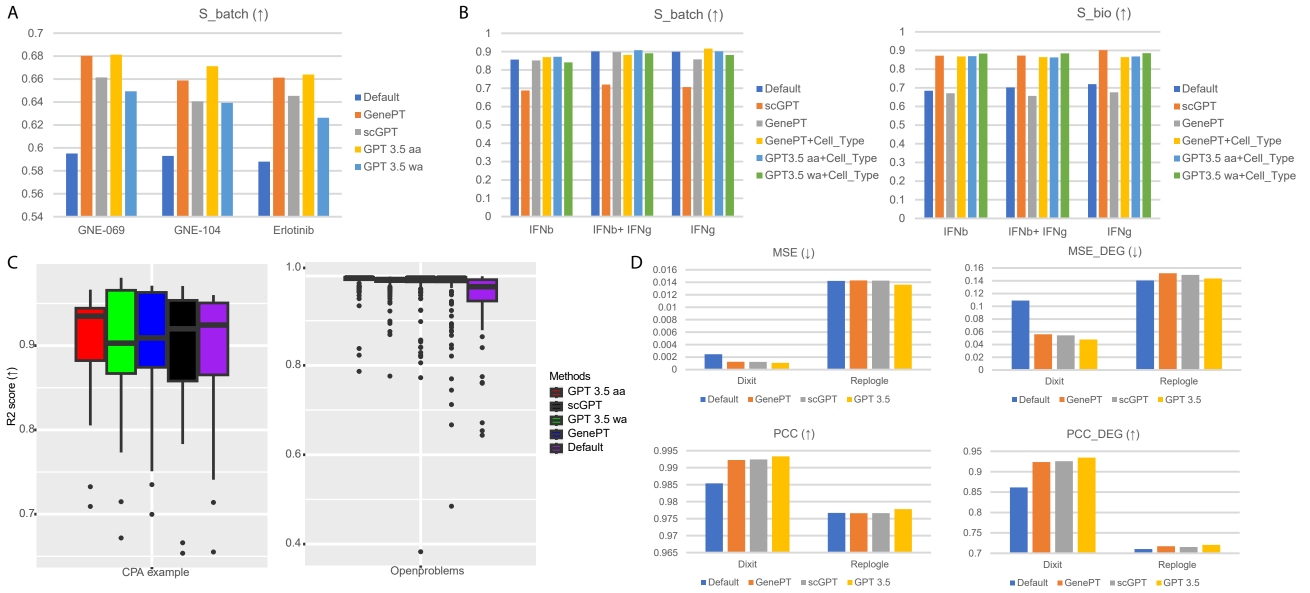

Figure 5. Results of perturbation analysis

(A) Scores of causal factor analysis for the Chang et al. dataset[106,107] based on CINEMA-OT across different input data. We considered four different types of extension. We still use S_{batch} to represent the levels of perturbation effect removal because we used the metrics for benchmarking batch integration.

(B) Scores of causal factor analysis for perturbed PBMC dataset[55] based on CINEMA-OT across different input data.

(C) Scores of gene expression prediction under different perturbation cases based on CPA. We considered four different methods and two datasets.

(D) Scores of gene expression prediction using perturb-seq datasets based on GEARS. We considered three different methods and three datasets.

CPA is a tool based on CVAE to predict the gene expression levels for the OOD samples of scRNA-seq data under chemical perturbations. Here, we combined the gene embeddings from scELMo with the original input dataset and learned a latent space for gene expression prediction. We investigated the contribution of gene embeddings by comparing the R2 score between these two different settings. We computed the R2 score based on the predicted gene expression levels and the observed gene expression levels. Figure 5C shows the performance of CPA under different methods for two datasets. For the CPA example dataset shown on the left, using the cell embeddings from GenePT and GPT-3.5 in the training process slightly improved the average R2 score, while its median value was still lower than the default mode. For the Openproblems dataset[108] shown on the right, using cell embeddings from scGPT, GPT-3.5, and GenePT improved the performance of CPA. Moreover, the R2 score based on combining cell embeddings with CPA had a higher average value and lower variance compared with the default mode. We further performed Wilcoxon rank sum tests for the R2 scores between different conditions and set the significant threshold for p values as 0.05. Based on our computation, the pairs of settings with significant differences include GPT-3.5 *aa* vs. default (p = 0.003) and GPT-3.5 *aa* vs. GenePT (p = 0.002) based on the CPA example dataset and GPT-3.5 *aa* vs. default (p = 7e−11) and GPT-3.5 *wa* vs. default (p = 6e−12) based on the Openproblems dataset. Therefore, scELMo could improve the performance of CPA on the prediction task by introducing the cell embeddings into the training process.

GEARS is a tool based on GNNs[109] to predict the gene expression levels for perturb-seq-based datasets. Here, we combined the gene embeddings from scELMo with the original gene embeddings of GEARS to learn the predicted value of target genes. We studied the contribution of gene embeddings by comparing the PCCs and MSEs between the default settings and updated settings when all genes were considered and when only DEGs were considered. The results are summarized in Figures 5D and S14A. For both Replogle[110] and Dixit[105] datasets, scELMo based on gene embeddings from GPT-3.5 outperformed the default settings as well as scELMo based on gene embeddings from GenePT and scGPT, supported by the comparisons based on all the metrics. For the Dixit dataset, using gene embeddings from GPT-3.5 obviously improved the gene expression prediction made by GEARS for both the all-genes case and the DEGs case. By considering the four datasets (Dixit, Norman,[111] Adamson,[112] and Replogle[110]) together, we performed a Wilcoxon rank sum test between the PCC and the PCC_DEG of scELMo and the default mode, and the improvement of scELMo was significant (p = 0.027). Furthermore, we also simulated datasets with different numbers of cells based on the Dixit dataset to test the robustness and consistency of our improvement, inspired by Wenteler et al.[113] Figure S14B shows that scELMo has an overall better performance as well as a lower variance compared with the default mode. Therefore, we believe that the introduction of gene embeddings from LLMs can also contribute to perturbation effect prediction by incorporating more knowledge about perturbed genes.

DISCUSSION

Modeling genetic and cellular functionality is a central challenge in computational biology. Traditionally, biological questions are

addressed by designing targeted experiments. However, the advent of FMs has introduced a complementary paradigm that offers generalized, data-driven tools capable of addressing a wide range of known and unforeseen biological questions. In the context of single-cell data analysis, many recent efforts have focused on developing large-scale pre-trained models, often referred to as FMs, and validating them across diverse downstream tasks. While such models demonstrate competitive performance, it is often difficult to identify tasks that truly require resource-intensive pre-training and fine-tuning pipelines.[16] This raises questions about the necessity and efficiency of such approaches. Motivated by GenePT, we explored an alternative strategy for building FMs in single-cell biology—leveraging the generative and representational capabilities of LLMs to produce meaningful feature- and cell-level embeddings. These embeddings can be used directly for unsupervised tasks, such as clustering and batch effect correction, or can be combined with task-specific models to enhance their performance in supervised settings. Both strategies are unified within the scELMo framework. Importantly, the gene embeddings generated by scELMo are robust to stochasticity, as demonstrated in Figure S15, where downstream performances exhibit low variance across different random seeds. Furthermore, accessing LLM-derived embeddings (e.g., via GPT-3.5) does not require substantial computational resources, making this approach highly cost effective. The comprehensive results presented here highlight the capability of scELMo to address a range of biological questions efficiently, without the need for extensive pre-training.

For scELMo under the zero-shot learning framework, we could utilize cell embeddings to perform clustering and batch effect correction. These contributions are based on the fact that embeddings from the text description of features in single-cell datasets are good for representing biological concepts or functions. We also discussed the factors that could affect the performance of generating meaningful cell embeddings, including the approach to computing the average embeddings, the number of cells in one dataset, the number of features we need, and other factors. We showed that such embeddings could be used for multi-omics data analysis, which illustrates the power of using LLMs as tools for incorporating prior information to enhance task-specific analysis.

Considering the limitations of the zero-shot learning framework, we also proposed a fine-tuning framework for scELMo. By combining the feature embeddings from GPT-3.5 with a light-structured neural network, we could use the embeddings to annotate cell types with performance similar to those of FMs that require many more resources for pre-training and fine-tuning. Moreover, scELMo can also be used for detecting novel therapeutic targets by examining the change of embeddings corresponding to the removal of certain genes, supported by related biological experiments. We could also directly incorporate the cell embeddings or feature embeddings with task-specific models for better performance in modeling the data with perturbation. *In silico* treatment analysis and perturbation analysis are two challenging and important tasks with cell-level knowledge, which further support the potential of scELMo and related work.

We provide the following guidelines for users interested in applying scELMo. For unsupervised tasks such as clustering and batch effect correction, we recommend using the *wa* mode, as it more effectively incorporates quantitative gene expression information. In contrast, the *aa* mode is better suited for perturbation analysis, where the contribution of individual features is more evenly distributed. The rationale behind these recommendations is elaborated in the methods. Both embedding strategies—*wa* and *aa*—can be effectively applied to cell-type annotation and *in silico* treatment analysis. We encourage users to consider enriching their analyses by integrating relevant metadata embeddings based on the specific biological context and task requirements. Finally, we encourage the community to further explore and expand the capabilities of scELMo, particularly in novel or underexplored domains of single-cell and systems biology, as its modular design lends itself well to broad and creative applications.

However, scELMo also has the following limitations. Firstly, the rapid development of LLMs likely leads to embeddings better than GPT-3.5. With a more powerful LLM, we will have a better representation of features and cells. Secondly, LLMs could not generate meaningful information for genes that were recently discovered or analyzed. Although GPT-3.5 does not make up concepts for genes, the lack of knowledge still presents a question for the application of scELMo. This shortcoming might limit the performance of applying scELMo for low-resource biological data, such as cells from patients with rare diseases. Finally, extracting features from other biomedical data, such as GWAS[114] or scATAC-seq[115] data, will be difficult since the number of features for these data is quite large.

Looking ahead, we aim to develop a comprehensive database containing textual descriptions and corresponding embeddings of biological features generated by multiple LLMs. Such a resource would enable broader application of scELMo, including its use in OOD prediction tasks, such as those handled by models such as CPA. To further address limitations observed in perturbation modeling, we plan to incorporate additional contextual information—such as perturbation-specific annotations of target genes—and explore improved evaluation metrics tailored to perturbation-related analyses. In parallel, we aim to investigate more efficient strategies for developing gene-specific LLMs, which may allow for even richer contextual embeddings with reduced resource demands. Given that scELMo is intrinsically compatible with arbitrary biomedical datasets in tabular formats, we believe its use can be generalized to a variety of domains beyond single-cell analysis. We anticipate that future extensions of scELMo will continue to unlock novel applications and insights across computational biology and related fields.

RESOURCE AVAILABILITY

Lead contact

Further information and requests for resources and reagents should be directed to and will be fulfilled by the lead contact, Prof. Hongyu Zhao (hongyu.zhao@yale.edu).

Materials availability

This study did not generate new or unique reagents.

Data and code availability

The code of scELMo can be found at https://github.com/HelloWorldLTY/scELMo and has been archived at Zenodo.[116] The license is a MIT license. To generate the text descriptions and embeddings, we rely on the API of

OpenAI. To run scELMo, we rely on the Yale High-performance Computing Center (YCRC) and utilize one NVIDIA A5000 GPU with up to 30 GB RAM for fine-tuning. We utilize the bigmem node with up to 1,000 GB RAM for analysis, and the minimal RAM requirement to analyze ~1,000,000 cells is 70 GB.

We did not generate new sequencing datasets in this project. The feature embeddings used in this manuscript can be downloaded through the scELMo embedding library.[117] All of the data used in this study can be downloaded[18,55,57,77–79,87–90,95,106,108,110,118,119] using the following links:

- https://github.com/JackieHanLab/TOSICA
- https://drive.google.com/drive/folders/1LgFvJqWNq9BqHbuxB2tYf62kXs9KqL4t
- https://academic.oup.com/bioinformatics/article/38/16/3942/6623406
- https://www.biorxiv.org/content/10.1101/2022.05.09.490241v2.abstract
- https://www.ncbi.nlm.nih.gov/geo/query/acc.cgi?acc=GSE139369
- https://www.ncbi.nlm.nih.gov/geo/query/acc.cgi?acc=GSE174072
- https://www.nature.com/articles/s41591-021-01329-2
- https://www.nature.com/articles/s41591-023-02327-2
- https://www.nature.com/articles/s41586-020-2797-4
- https://www.nature.com/articles/s41586-022-04817-8
- http://projects.sanderlab.org/scperturb/datavzrd/scPerturb_vzrd_v1/dataset_info/index_1.html
- https://github.com/vandijklab/CINEMA-OT/tree/main
- https://www.kaggle.com/competitions/open-problems-single-cell-perturbations
- https://cellxgene.cziscience.com/e/3faad104-2ab8-4434-816d-474d8d2641db.cxg/
- https://www.nature.com/articles/s41587-023-01905-6
- https://www.sciencedirect.com/science/article/pii/S0092867422005979?via%3Dihub

The download links and data statistics of all data are summarized in Data S3. The source data of the presented figures are available in Data S4.

ACKNOWLEDGMENTS

We thank Gefei Wang and Chen Lin for the suggestions on datasets. We thank Mingze Dong for helpful discussions. We also acknowledge ChatGPT for helping us revise the text. This project is supported in part by NIH grants U24HG012108 and U01HG013840.

AUTHOR CONTRIBUTIONS

T.L. designed this study. T.L., X.L., and Y.C. designed the model. T.L., T.C., and W.Z. ran all the experiments. T.L. and H.Z. wrote the manuscript. H.Z. supervised this work. We did not use AI tools to write this manuscript.

DECLARATION OF INTERESTS

The authors declare no competing interests.

SUPPLEMENTAL INFORMATION

Supplemental information can be found online at https://doi.org/10.1016/j.patter.2025.101431.

Received: May 2, 2025
Revised: August 20, 2025
Accepted: November 6, 2025
Published: January 30, 2026

REFERENCES

1. Wu, J., Yang, S., Zhan, R., Yuan, Y., Chao, L.S., and Wong, D.F. (2025). A Survey on LLM-Generated Text Detection: Necessity, Methods, and Future Directions. Comput. Linguist. *51*, 275–338.
2. Zhao, W.X., Zhou, K., Li, J., Tang, T., Wang, X., Hou, Y., Min, Y., Zhang, B., Zhang, J., Dong, Z., et al. (2023). A survey of large language modelspreprint at arXiv. https://doi.org/10.48550/arXiv.2303.18223.
3. Zhou, C., Li, Q., Li, C., Yu, J., Liu, Y., Wang, G., Zhang, K., Ji, C., Yan, Q., He, L., et al. (2025). A comprehensive survey on pretrained foundation models: A history from bert to chatgpt. Int. J. Mach. Learn. Cybern. *16*, 9851–9915.
4. Nguyen, E., Poli, M., Faizi, M., Thomas, A. W., Wornow, M., Birch-Sykes, C., Massaroli, S., Patel, A., Rabideau, C. M., Bengio, Y., et al. (2023). "HyenaDNA: Long-Range Genomic Sequence Modeling at Single Nucleotide Resolution". Thirty-seventh Conference on Neural Information Processing Systems.
5. Marin, F. I., Teufel, F., Horlacher, M., Madsen, D., Pultz, D., Winther, O., and Boomsma, W. (2024). "BEND: Benchmarking DNA Language Models on Biologically Meaningful Tasks". The Twelfth International Conference on Learning Representations.
6. Fan, Y., Li, Y., Ding, J., and Li, Y. (2024). GFETM: Genome Foundation-Based Embedded Topic Model for scATAC-seq Modeling. In Research in Computational Molecular Biology, J. Ma, ed. (Cham: Springer Nature Switzerland), pp. 314–319.
7. Cui, H., Wang, C., Maan, H., Pang, K., Luo, F., Duan, N., and Wang, B. (2024). scGPT: toward building a foundation model for single-cell multi-omics using generative AI. Nat. Methods *21*, 1470–1480.
8. Theodoris, C.V., Xiao, L., Chopra, A., Chaffin, M.D., Al Sayed, Z.R., Hill, M.C., Mantineo, H., Brydon, E.M., Zeng, Z., Liu, X.S., and Ellinor, P.T. (2023). Transfer learning enables predictions in network biology. Nature *618*, 616–624.
9. Yang, F., Wang, W., Wang, F., Fang, Y., Tang, D., Huang, J., Lu, H., and Yao, J. (2022). scBERT as a large-scale pretrained deep language model for cell type annotation of single-cell RNA-seq data. Nat. Mach. Intell. *4*, 852–866.
10. Han, X., Zhou, Z., Fei, L., Sun, H., Wang, R., Chen, Y., Chen, H., Wang, J., Tang, H., Ge, W., et al. (2020). Construction of a human cell landscape at single-cell level. Nature *581*, 303–309.
11. Saliba, A.-E., Westermann, A.J., Gorski, S.A., and Vogel, J. (2014). Single-cell RNA-seq: advances and future challenges. Nucleic Acids Res. *42*, 8845–8860.
12. Cheung, R.K., and Utz, P.J. (2011). CyTOF—the next generation of cell detection. Nat. Rev. Rheumatol. *7*, 502–503.
13. Stoeckius, M., Hafemeister, C., Stephenson, W., Houck-Loomis, B., Chattopadhyay, P.K., Swerdlow, H., Satija, R., and Smibert, P. (2017). Simultaneous epitope and transcriptome measurement in single cells. Nat. Methods *14*, 865–868.
14. Karemaker, I.D., and Vermeulen, M. (2018). Single-cell DNA methylation profiling: technologies and biological applications. Trends Biotechnol. *36*, 952–965.
15. Hao, M., Gong, J., Zeng, X., Liu, C., Guo, Y., Cheng, X., Wang, T., Ma, J., Zhang, X., and Song, L. (2024). Large-scale foundation model on single-cell transcriptomics. Nat. Methods *21*, 1481–1491.
16. Liu, T., Li, K., Wang, Y., Li, H., and Zhao, H. (2024). Evaluating the Utilities of Foundation Models in Single-cell Data Analysis. bioRxiv. https://doi.org/10.1101/2023.09.08.555192.
17. Kedzierska, K.Z., Crawford, L., Amini, A.P., and Lu, A.X. (2025). Zero-shot evaluation reveals limitations of single-cell foundation models. Genome Biol. *26*, 101.
18. Chen, Y., and Zou, J. (2025). Simple and effective embedding model for single-cell biology built from chatgpt. Nat. Biomed. Eng. *9*, 483–493.
19. Wheeler, D.L., Barrett, T., Benson, D.A., Bryant, S.H., Canese, K., Chetvernin, V., Church, D.M., DiCuccio, M., Edgar, R., Federhen, S., et al. (2007). Database resources of the national center for biotechnology information. Nucleic Acids Res. *35*, D5–D12.
20. Shevlane, T. (n.d.). "Structured Access: An Emerging Paradigm for Safe AI Deployment". The Oxford Handbook of AI Governance. Oxford

University Press. isbn: 9780197579329. https://doi.org/10.1093/oxfordhb/9780197579329.013.39.eprint: https://academic.oup.com/book/0/chapter/355438814/chapter-ag-pdf/54874439/book_41989_section_355438814.ag.pdf.

21. Brown, T., Mann, B., Ryder, N., Subbiah, M., Kaplan, J.D., Dhariwal, P., Neelakantan, A., Shyam, P., Sastry, G., Askell, A., et al. (2020). Language models are few-shot learners. Adv. Neural Inf. Process. Syst. *33*, 1877–1901.
22. OpenAI (2023). GPT-4 Technical Reportpreprint at arXiv. https://doi.org/10.48550/arXiv.2303.08774.
23. Touvron, H., Lavril, T., Izacard, G., Martinet, X., Lachaux, M.-A., Lacroix, T., Rozière, B., Goyal, N., Hambro, E., Azhar, F., et al. (2023). Llama: Open and Efficient Foundation Language Modelspreprint at arXiv. https://doi.org/10.48550/arXiv.2302.13971.
24. Xiao, L., and Chen, X. (2023). Enhancing LLM with Evolutionary Fine Tuning for News Summary Generation. Preprint at arXiv. https://doi.org/10.48550/arXiv.2307.02839.
25. Jawahar, G., Abdul-Mageed, M., Lakshmanan, L., and Ding, D. (2024). LLM Performance Predictors are good initializers for Architecture Search. In Findings of the Association for Computational Linguistics ACL 2024, L.-W. Ku, A. Martins, and V.S. Bangkok, eds. (Association for Computational Linguistics), pp. 10540–10560.
26. Kumar, V., Gleyzer, L., Kahana, A., Shukla, K., and Karniadakis, G.E. (2023). MYCRUNCHGPT: A LLM ASSISTED FRAMEWORK FOR SCIENTIFIC MACHINE LEARNING. J. Mach. Learn. Model. Comput. *4*, 41–72.
27. Varghese, J., and Chapiro, J. (2023). ChatGPT: The transformative influence of generative AI on science and healthcare. J. Hepatol. *80*, 977–980.
28. Peters, M., Neumann, M., Iyyer, M., Gardner, M., Clark, C., Lee, K., and Zettlemoyer, L. (2018). Deep Contextualized Word Representations. In Proceedings of the 2018 Conference of the North American Chapter of the Association for Computational Linguistics: Human Language Technologies, *1*, M. Walker, H. Ji, and A. Stent, eds. (New Orleans, Louisiana: Association for Computational Linguistics), pp. 2227–2237. Long Papers.
29. Wolf, F.A., Angerer, P., and Theis, F.J. (2018). SCANPY: large-scale single-cell gene expression data analysis. Genome Biol. *19*, 15.
30. Chu, S.-K., Zhao, S., Shyr, Y., and Liu, Q. (2022). Comprehensive evaluation of noise reduction methods for single-cell RNA sequencing data. Brief. Bioinform. *23*, bbab565.
31. Gao, Y., Myers, S., Chen, S., Dligach, D., Miller, T.A., Bitterman, D., Churpek, M., and Afshar, M. (2024). When Raw Data Prevails: Are Large Language Model Embeddings Effective in Numerical Data Representation for Medical Machine Learning Applications? In Findings of the Association for Computational Linguistics: EMNLP 2024, Y. Al-Onaizan, M. Bansal, and Y.-N. Chen, eds. (Miami, Florida, USA: Association for Computational Linguistics), pp. 5414–5428. https://doi.org/10.18653/v1/2024.findings-emnlp.311.
32. Chen, T., Kornblith, S., Norouzi, M., and Hinton, G. (2020). "A Simple Framework for Contrastive Learning of Visual Representations". International Conference on Machine Learning (PMLR), pp. 1597–1607.
33. Musgrave, K., Belongie, S., and Lim, S.-N. (2020). PyTorch Metric Learningpreprint at arXiv. https://doi.org/10.48550/arXiv.2008.09164.
34. Agarap, A.F. (2018). Deep Learning Using Rectified Linear Units (ReLU)preprint at arXiv. https://doi.org/10.48550/arXiv.1803.08375.
35. Harris, C.R., Millman, K.J., Van Der Walt, S.J., Gommers, R., Virtanen, P., Cournapeau, D., Wieser, E., Taylor, J., Berg, S., Smith, N.J., et al. (2020). Array programming with NumPy. Nature *585*, 357–362.
36. Ashburner, M., Ball, C.A., Blake, J.A., Botstein, D., Butler, H., Cherry, J.M., Davis, A.P., Dolinski, K., Dwight, S.S., Eppig, J.T., et al. (2000). Gene ontology: tool for the unification of biology. Nat. Genet. *25*, 25–29.
37. Gene Ontology Consortium, Aleksander, S.A., Balhoff, J., Carbon, S., Cherry, J.M., Drabkin, H.J., Ebert, D., Feuermann, M., Gaudet, P., Harris, N.L., et al. (2023). The gene ontology knowledgebase in 2023. Genetics *224*, iyad031.
38. Fang, Z., Liu, X., and Peltz, G. (2023). GSEApy: a comprehensive package for performing gene set enrichment analysis in Python. Bioinformatics *39*, btac757.
39. Krämer, A., Green, J., Pollard, J., Jr., and Tugendreich, S. (2014). Causal analysis approaches in ingenuity pathway analysis. Bioinformatics *30*, 523–530.
40. Gayoso, A., Steier, Z., Lopez, R., Regier, J., Nazor, K.L., Streets, A., and Yosef, N. (2021). Joint probabilistic modeling of single-cell multi-omic data with totalVI. Nat. Methods *18*, 272–282.
41. Zhu, B., Chen, S., Bai, Y., Chen, H., Liao, G., Mukherjee, N., Vazquez, G., McIlwain, D.R., Tzankov, A., Lee, I.T., et al. (2023). Robust single-cell matching and multimodal analysis using shared and distinct features. Nat. Methods *20*, 304–315.
42. Papineni, K., Roukos, S., Ward, T., and Zhu, W.-J. (2002). "Bleu: a method for automatic evaluation of machine translation". Proceedings of the 40th annual meeting of the Association for Computational Linguistics, 311–318.
43. Freitag, M., Foster, G., Grangier, D., Ratnakar, V., Tan, Q., and Macherey, W. (2021). Experts, errors, and context: A large-scale study of human evaluation for machine translation. Transactions of the Association for Computational Linguistics *9*, 1460–1474.
44. Luecken, M.D., Büttner, M., Chaichoompu, K., Danese, A., Interlandi, M., Müller, M.F., Strobl, D.C., Zappia, L., Dugas, M., Colomé-Tatché, M., and Theis, F.J. (2022). Benchmarking atlas-level data integration in single-cell genomics. Nat. Methods *19*, 41–50.
45. Pedregosa, F., Varoquaux, G., Gramfort, A., Michel, V., Thirion, B., Grisel, O., Blondel, M., Prettenhofer, P., Weiss, R., Dubourg, V., et al. (2011). Scikit-learn: Machine learning in Python. the Journal of machine Learning research *12*, 2825–2830.
46. Virtanen, P., Gommers, R., Oliphant, T.E., Haberland, M., Reddy, T., Cournapeau, D., Burovski, E., Peterson, P., Weckesser, W., Bright, J., et al. (2020). SciPy 1.0: fundamental algorithms for scientific computing in Python. Nat. Methods *17*, 261–272.
47. Suzgun, M., and Kalai, A.T. (2024). Meta-Prompting: Enhancing Language Models with Task-Agnostic Scaffoldingpreprint at arXiv. https://doi.org/10.48550/arXiv.2401.12954.
48. Kiselev, V.Y., Kirschner, K., Schaub, M.T., Andrews, T., Yiu, A., Chandra, T., Natarajan, K.N., Reik, W., Barahona, M., Green, A.R., and Hemberg, M. (2017). SC3: consensus clustering of single-cell RNA-seq data. Nat. Methods *14*, 483–486.
49. Lopez, R., Regier, J., Cole, M.B., Jordan, M.I., and Yosef, N. (2018). Deep generative modeling for single-cell transcriptomics. Nat. Methods *15*, 1053–1058.
50. Korsunsky, I., Millard, N., Fan, J., Slowikowski, K., Zhang, F., Wei, K., Baglaenko, Y., Brenner, M., Loh, P.-r., and Raychaudhuri, S. (2019). Fast, sensitive and accurate integration of single-cell data with Harmony. Nat. Methods *16*, 1289–1296.
51. Haghverdi, L., Lun, A.T.L., Morgan, M.D., and Marioni, J.C. (2018). Batch effects in single-cell RNA-sequencing data are corrected by matching mutual nearest neighbors. Nat. Biotechnol. *36*, 421–427.
52. Radford, A., Wu, J., Child, R., Luan, D., Amodei, D., and Sutskever, I. (2019). Language models are unsupervised multitask learners. OpenAI blog *1*, 9.
53. Hou, W., and Ji, Z. (2024). Assessing GPT-4 for cell type annotation in single-cell RNA-seq analysis. Nat. Methods *21*, 1462–1465.
54. Devlin, J., Chang, M.-W., Lee, K., and Toutanova, K. (2019). BERT: Pre-training of Deep Bidirectional Transformers for Language Understanding. In Proceedings of the 2019 Conference of the North American Chapter of the Association for Computational Linguistics: Human Language Technologies, Volume 1 (Long and Short Papers), J. Burstein, C. Doran, and T. Solorio, eds. (Minneapolis, Minnesota: Association for Computational Linguistics), pp. 4171–4186.

CellPress
OPEN ACCESS

55. Dong, M., Wang, B., Wei, J., de O Fonseca, A.H., Perry, C.J., Frey, A., Ouerghi, F., Foxman, E.F., Ishizuka, J.J., Dhodapkar, R.M., and van Dijk, D. (2023). Causal identification of single-cell experimental perturbation effects with CINEMA-OT. Nat. Methods *20*, 1769–1779.

56. Lotfollahi, M., Klimovskaia Susmelj, A., De Donno, C., Hetzel, L., Ji, Y., Ibarra, I.L., Srivatsan, S.R., Naghipourfar, M., Daza, R.M., Martin, B., et al. (2023). Predicting cellular responses to complex perturbations in high-throughput screens. Mol. Syst. Biol. *19*, e11517.

57. Roohani, Y., Huang, K., and Leskovec, J. (2023). Predicting transcriptional outcomes of novel multigene perturbations with gears. Nat. Biotechnol. *42*, 927–935.

58. Landa, B., Zhang, T.T.C.K., and Kluger, Y. (2022). Biwhitening reveals the rank of a count matrix. SIAM J. Math. Data Sci. *4*, 1420–1446.

59. Cuturi, M. (2013). Sinkhorn distances: Lightspeed computation of optimal transport. Adv. Neural Inf. Process. Syst. *26*.

60. Petukhova, A., Matos-Carvalho, J.P., and Fachada, N. (2025). Text clustering with large language model embeddings. International Journal of Cognitive Computing in Engineering *6*, 100–108.

61. Keraghel, I., Morbieu, S., and Nadif, M. (2024). "Beyond Words: A Comparative Analysis of LLM Embeddings for Effective Clustering". International Symposium on Intelligent Data Analysis (Springer), pp. 205–216.

62. Asudani, D.S., Nagwani, N.K., and Singh, P. (2023). Impact of word embedding models on text analytics in deep learning environment: a review. Artif. Intell. Rev. *56*, 1–81.

63. Cao, Z.-J., and Gao, G. (2022). Multi-omics single-cell data integration and regulatory inference with graph-linked embedding. Nat. Biotechnol. *40*, 1458–1466.

64. Huang, K., Lopez, R., Hütter, J.-C., Kudo, T., Rios, A., and Regev, A. (2024). "Sequential Optimal Experimental Design of Perturbation Screens Guided by Multi-Modal Priors". International Conference on Research in Computational Molecular Biology (Springer), pp. 17–37.

65. Zhang, Y., Li, Y., Cui, L., Cai, D., Liu, L., Fu, T., Huang, X., Zhao, E., Zhang, Y., Chen, Y., et al. (2023). Siren's Song in the AI Ocean: A Survey on Hallucination in Large Language Modelspreprint at arXiv. https://doi.org/10.48550/arXiv.2309.01219.

66. Jiang, A.Q., Sablayrolles, A., Mensch, A., Bamford, C., Chaplot, D.S., Casas, D. de las, Bressand, F., Lengyel, G., Lample, G., Saulnier, L., et al. (2023). Mistral 7Bpreprint at arXiv. https://doi.org/10.48550/arXiv.2310.06825.

67. Luo, R., Sun, L., Xia, Y., Qin, T., Zhang, S., Poon, H., and Liu, T.-Y. (2022). BioGPT: generative pre-trained transformer for biomedical text generation and mining. Brief. Bioinform. *23*, bbac409.

68. Anthropic, A. (2023). Model Card and Evaluations for Claude Models (Anthropic Blog).

69. Anil, R., Dai, A.M., Firat, O., Johnson, M., Lepikhin, D., Passos, A., Shakeri, S., Taropa, E., Bailey, P., Chen, Z., et al. (2023). PaLM 2 Technical Reportpreprint at arXiv. https://doi.org/10.48550/arXiv.2305.10403.

70. Hao, Y., Hao, S., Andersen-Nissen, E., Mauck, W.M., Zheng, S., Butler, A., Lee, M.J., Wilk, A.J., Darby, C., Zager, M., et al. (2021). Integrated analysis of multimodal single-cell data. Cell *184*, 3573–3587.e29.

71. Safran, M., Dalah, I., Alexander, J., Rosen, N., Iny Stein, T., Shmoish, M., Nativ, N., Bahir, I., Doniger, T., Krug, H., et al. (2010). GeneCards Version 3: the human gene integrator. Database *2010*, baq020.

72. Wei, J., Wang, X., Schuurmans, D., Bosma, M., Ichter, B., Xia, F., Chi, E., Le, Q.V., Zhou, D., et al. (2022). Chain-of-thought prompting elicits reasoning in large language models. Adv. Neural Inf. Process. Syst. *35*, 24824–24837.

73. Martin, F.J., Amode, M.R., Aneja, A., Austine-Orimoloye, O., Azov, A.G., Barnes, I., Becker, A., Bennett, R., Berry, A., Bhai, J., et al. (2023). Ensembl 2023. Nucleic Acids Res. *51*, D933–D941.

74. Soneson, C., and Robinson, M.D. (2018). Bias, robustness and scalability in single-cell differential expression analysis. Nat. Methods *15*, 255–261.

75. Armingol, E., Officer, A., Harismendy, O., and Lewis, N.E. (2021). Deciphering cell–cell interactions and communication from gene expression. Nat. Rev. Genet. *22*, 71–88.

76. McInnes, L., Healy, J., Saul, N., and Großberger, L. (2018). UMAP: Uniform Manifold Approximation and Projection. J. Open Source Softw. *3*, 861. https://doi.org/10.21105/joss.00861.

77. Stephenson, E., Reynolds, G., Botting, R.A., Calero-Nieto, F.J., Morgan, M.D., Tuong, Z.K., Bach, K., Sungnak, W., Worlock, K.B., Yoshida, M., et al. (2021). Single-cell multi-omics analysis of the immune response in COVID-19. Nat. Med. *27*, 904–916.

78. Sikkema, L., Ramírez-Suástegui, C., Strobl, D.C., Gillett, T.E., Zappia, L., Madissoon, E., Markov, N.S., Zaragosi, L.-E., Ji, Y., Ansari, M., et al. (2023). An integrated cell atlas of the lung in health and disease. Nat. Med. *29*, 1563–1577.

79. Litviňuková, M., Talavera-López, C., Maatz, H., Reichart, D., Worth, C.L., Lindberg, E.L., Kanda, M., Polanski, K., Heinig, M., Lee, M., et al. (2020). Cells of the adult human heart. Nature *588*, 466–472.

80. Miao, Z., Humphreys, B.D., McMahon, A.P., and Kim, J. (2021). Multi-omics integration in the age of million single-cell data. Nat. Rev. Nephrol. *17*, 710–724.

81. Zeng, H. (2022). What is a cell type and how to define it? Cell *185*, 2739–2755.

82. Baron, M., Veres, A., Wolock, S.L., Faust, A.L., Gaujoux, R., Vetere, A., Ryu, J.H., Wagner, B.K., Shen-Orr, S.S., Klein, A.M., et al. (2016). A single-cell transcriptomic map of the human and mouse pancreas reveals inter-and intra-cell population structure. Cell Syst. *3*, 346–360.e4.

83. Muraro, M.J., Dharmadhikari, G., Grün, D., Groen, N., Dielen, T., Jansen, E., Van Gurp, L., Engelse, M.A., Carlotti, F., de Koning, E.J.P., and van Oudenaarden, A. (2016). A single-cell transcriptome atlas of the human pancreas. Cell Syst. *3*, 385–394.e3.

84. Xin, Y., Kim, J., Okamoto, H., Ni, M., Wei, Y., Adler, C., Murphy, A.J., Yancopoulos, G.D., Lin, C., and Gromada, J. (2016). RNA sequencing of single human islet cells reveals type 2 diabetes genes. Cell Metab. *24*, 608–615.

85. Lawlor, N., George, J., Bolisetty, M., Kursawe, R., Sun, L., Sivakamasundari, V., Kycia, I., Robson, P., and Stitzel, M.L. (2017). Single-cell transcriptomes identify human islet cell signatures and reveal cell-type–specific expression changes in type 2 diabetes. Genome Res. *27*, 208–222.

86. Li, Y., Ren, P., Dawson, A., Vasquez, H.G., Ageedi, W., Zhang, C., Luo, W., Chen, R., Li, Y., Kim, S., et al. (2020). Single-cell transcriptome analysis reveals dynamic cell populations and differential gene expression patterns in control and aneurysmal human aortic tissue. Circulation *142*, 1374–1388.

87. Wang, Y., Liu, T., and Zhao, H. (2022). ResPAN: a powerful batch correction model for scRNA-seq data through residual adversarial networks. Bioinformatics *38*, 3942–3949.

88. Pullin, J.M., and McCarthy, D.J. (2024). A comparison of marker gene selection methods for single-cell RNA sequencing data. Genome Biol. *25*, 56.

89. Granja, J.M., Klemm, S., McGinnis, L.M., Kathiria, A.S., Mezger, A., Corces, M.R., Parks, B., Gars, E., Liedtke, M., Zheng, G.X.Y., et al. (2019). Single-cell multiomic analysis identifies regulatory programs in mixed-phenotype acute leukemia. Nat. Biotechnol. *37*, 1458–1465.

90. Wilk, A.J., Lee, M.J., Wei, B., Parks, B., Pi, R., Martínez-Colón, G.J., Ranganath, T., Zhao, N.Q., Taylor, S., Becker, W., et al. (2021). Multi-omic profiling reveals widespread dysregulation of innate immunity and hematopoiesis in COVID-19. J. Exp. Med. *218*, e20210582.

91. Li, Y.Y., An, J., and Jones, S.J.M. (2011). A computational approach to finding novel targets for existing drugs. PLoS Comput. Biol. *7*, e1002139.

92. Kumar, R., and Saha, P. (2022). A review on artificial intelligence and machine learning to improve cancer management and drug discovery. International Journal for Research in Applied Sciences and Biotechnology *9*, 149–156.

93. Abdelazim, M.A., Nasr, M.M., and Ead, W.M. (2020). A survey on classification analysis for cancer genomics: Limitations and novel opportunity in the era of cancer classification and Target Therapies. Ann. Trop. Med. Public Health *23*, 24.

94. Spudich, J.A. (2014). Hypertrophic and dilated cardiomyopathy: four decades of basic research on muscle lead to potential therapeutic approaches to these devastating genetic diseases. Biophys. J. *106*, 1236–1249.

95. Chaffin, M., Papangeli, I., Simonson, B., Akkad, A.-D., Hill, M.C., Arduini, A., Fleming, S.J., Melanson, M., Hayat, S., Kost-Alimova, M., et al. (2022). Single-nucleus profiling of human dilated and hypertrophic cardiomyopathy. Nature *608*, 174–180.

96. Pagiatakis, C., and Di Mauro, V. (2021). The emerging role of epigenetics in therapeutic targeting of cardiomyopathies. Int. J. Mol. Sci. *22*, 8721.

97. Fang, C., Lv, Z., Yu, Z., Wang, K., Xu, C., Li, Y., and Wang, Y. (2022). Exploration of dilated cardiomyopathy for biomarkers and immune microenvironment: Evidence from RNA-seq. BMC Cardiovasc. Disord. *22*, 320.

98. Feng, Y., Cai, L., Hong, W., Zhang, C., Tan, N., Wang, M., Wang, C., Liu, F., Wang, X., Ma, J., et al. (2022). Rewiring of 3D chromatin topology orchestrates transcriptional reprogramming and the development of human dilated cardiomyopathy. Circulation *145*, 1663–1683.

99. Balashanmugam, M.V., Shivanandappa, T.B., Nagarethinam, S., Vastrad, B., and Vastrad, C. (2019). Analysis of differentially expressed genes in coronary artery disease by integrated microarray analysis. Biomolecules *10*, 35.

100. Barrangou, R., and Doudna, J.A. (2016). Applications of CRISPR technologies in research and beyond. Nat. Biotechnol. *34*, 933–941.

101. Davies, R.R., Kaple, R.K., Mandapati, D., Gallo, A., Botta, D.M., Jr., Elefteriades, J.A., and Coady, M.A. (2007). Natural history of ascending aortic aneurysms in the setting of an unreplaced bicuspid aortic valve. Ann. Thorac. Surg. *83*, 1338–1344.

102. Moreno-Loshuertos, R., Movilla, N., Marco-Brualla, J., Soler-Agesta, R., Ferreira, P., Enríquez, J.A., and Fernández-Silva, P. (2023). A Mutation in Mouse MT-ATP6 Gene Induces Respiration Defects and Opposed Effects on the Cell Tumorigenic Phenotype. Int. J. Mol. Sci. *24*, 1300.

103. Stendel, C., Neuhofer, C., Floride, E., Yuqing, S., Ganetzky, R.D., Park, J., Freisinger, P., Kornblum, C., Kleinle, S., Schöls, L., et al. (2020). Delineating MT-ATP6-associated disease: From isolated neuropathy to early onset neurodegeneration. Neurol. Genet. *6*, e393.

104. Lotfollahi, M., Wolf, F.A., and Theis, F.J. (2019). scGen predicts single-cell perturbation responses. Nat. Methods *16*, 715–721.

105. Dixit, A., Parnas, O., Li, B., Chen, J., Fulco, C.P., Jerby-Arnon, L., Marjanovic, N.D., Dionne, D., Burks, T., Raychowdhury, R., et al. (2016). Perturb-Seq: dissecting molecular circuits with scalable single-cell RNA profiling of pooled genetic screens. cell *167*, 1853–1866.e17.

106. Green, T.D., Peidli, S., Shen, C., Gross, T., Min, J., Garda, S., Taylor-King, J.P., Marks, D.S., Luna, A., Blüthgen, N., et al. (2022). scPerturb: Information Resource for Harmonized Single-Cell Perturbation Data (NeurIPS 2022 Workshop on Learning Meaningful Representations of Life).

107. Chang, M.T., Shanahan, F., Nguyen, T.T.T., Staben, S.T., Gazzard, L., Yamazoe, S., Wertz, I.E., Piskol, R., Yang, Y.A., Modrusan, Z., et al. (2022). Identifying transcriptional programs underlying cancer drug response with TraCe-seq. Nat. Biotechnol. *40*, 86–93.

108. Szałata, A., Benz, A., Cannoodt, R., Cortes, M., Fong, J., Kuppasani, S., Lieberman, R., Liu, T., Mas-Rosario, J.A., Meinl, R., et al. (2024). A benchmark for prediction of transcriptomic responses to chemical perturbations across cell types. Adv. Neural Inf. Process. Syst. *37*, 20566–20616.

109. Kipf, T. N. and Welling, M. (2017). "Semi-Supervised Classification with Graph Convolutional Networks". International Conference on Learning Representations.

110. Replogle, J.M., Saunders, R.A., Pogson, A.N., Hussmann, J.A., Lenail, A., Guna, A., Mascibroda, L., Wagner, E.J., Adelman, K., Lithwick-Yanai, G., et al. (2022). Mapping information-rich genotype-phenotype landscapes with genome-scale Perturb-seq. Cell *185*, 2559–2575.e28.

111. Norman, T.M., Horlbeck, M.A., Replogle, J.M., Ge, A.Y., Xu, A., Jost, M., Gilbert, L.A., and Weissman, J.S. (2019). Exploring genetic interaction manifolds constructed from rich single-cell phenotypes. Science *365*, 786–793.

112. Adamson, B., Norman, T.M., Jost, M., Cho, M.Y., Nuñez, J.K., Chen, Y., Villalta, J.E., Gilbert, L.A., Horlbeck, M.A., Hein, M.Y., et al. (2016). A multiplexed single-cell CRISPR screening platform enables systematic dissection of the unfolded protein response. Cell *167*, 1867–1882.e21.

113. Wenteler, A., Occhetta, M., Branson, N., Curean, V., Huebner, M., Dee, W., Connell, W., Chung, S.P., Hawkins-Hooker, A., Ektefaie, Y., et al. (2025). PertEval-scFM: Benchmarking Single-Cell Foundation Models for Perturbation Effect Prediction. Forty-second International Conference on Machine Learning.

114. Visscher, P.M., Brown, M.A., McCarthy, M.I., and Yang, J. (2012). Five years of GWAS discovery. Am. J. Hum. Genet. *90*, 7–24.

115. Mimitou, E.P., Lareau, C.A., Chen, K.Y., Zorzetto-Fernandes, A.L., Hao, Y., Takeshima, Y., Luo, W., Huang, T.-S., Yeung, B.Z., Papalexi, E., et al. (2021). Scalable, multimodal profiling of chromatin accessibility, gene expression and protein levels in single cells. Nat. Biotechnol. *39*, 1246–1258.

116. Liu, T. (2025). scELMo. Zendo. https://doi.org/10.5281/zenodo.17298922.

117. (2025). scELMo embedding library. Zendo. https://doi.org/10.5281/zenodo.17517204.

118. Chen, J., Xu, H., Tao, W., Chen, Z., Zhao, Y., and Han, J.-D.J. (2023). Transformer for one stop interpretable cell type annotation. Nat. Commun. *14*, 223.

119. CZI Cell Science Program, Abdulla, S., Aevermann, B., Assis, P., Badajoz, S., Bell, S.M., Bezzi, E., Cakir, B., Chaffer, J., Chambers, S., et al. (2025). CZ CELLxGENE Discover: a single-cell data platform for scalable exploration, analysis and modeling of aggregated data. Nucleic Acids Res. *53*, D886–D900.

Patterns, Volume *7*

Supplemental information

Embeddings from language models are good learners for single-cell data analysis

Tianyu Liu, Tianqi Chen, Wangjie Zheng, Xiao Luo, Yiqun Chen, and Hongyu Zhao

Supplementary Figures

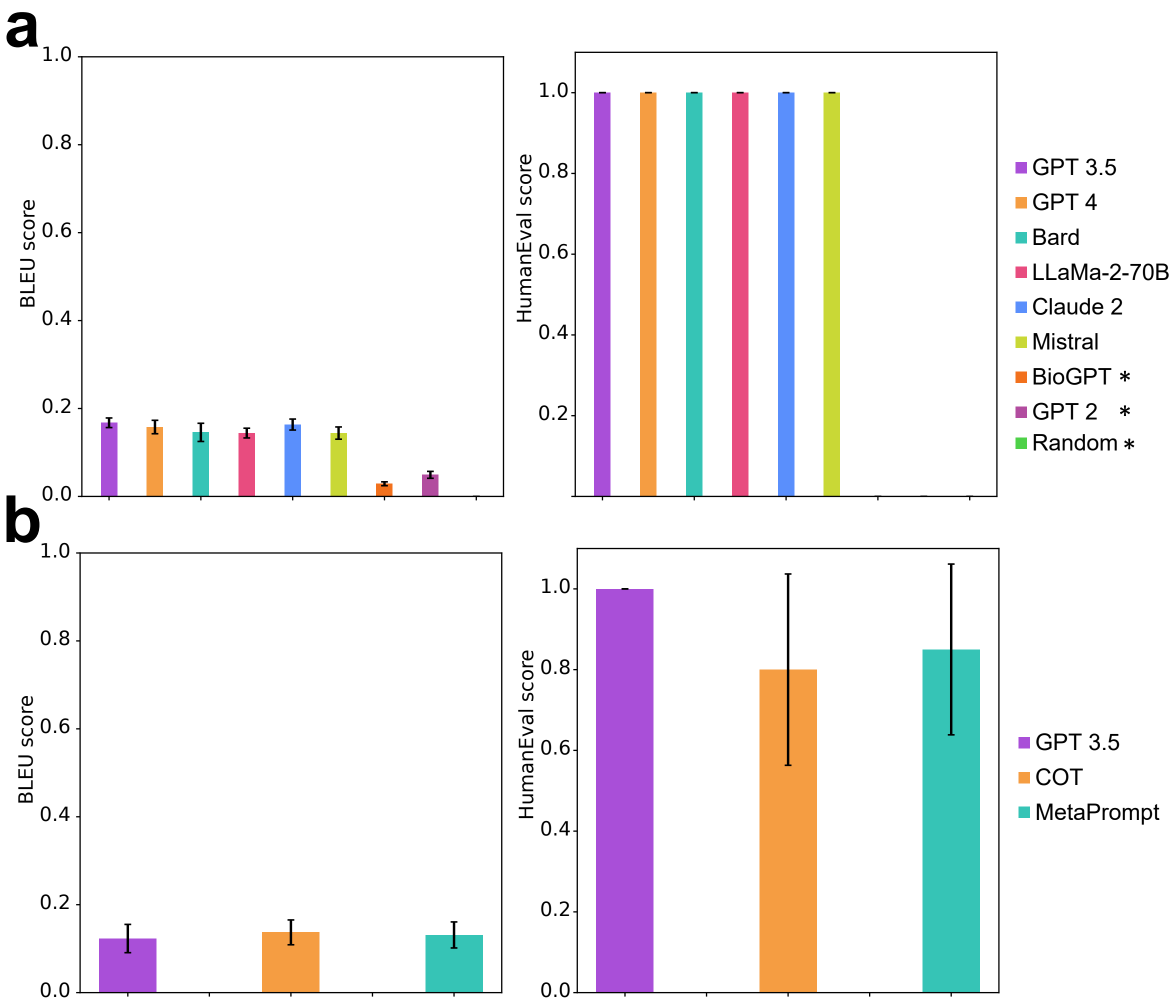

Supplementary Fig. 1. Evaluations of descriptions of cell types and prompt engineering approaches. (a) Metrics for evaluating meaningful outputs of cell types across different LLMs. (b) Metrics for evaluating meaningful outputs of cell types across different prompt engineering approaches. This figure is related to Figure 2.

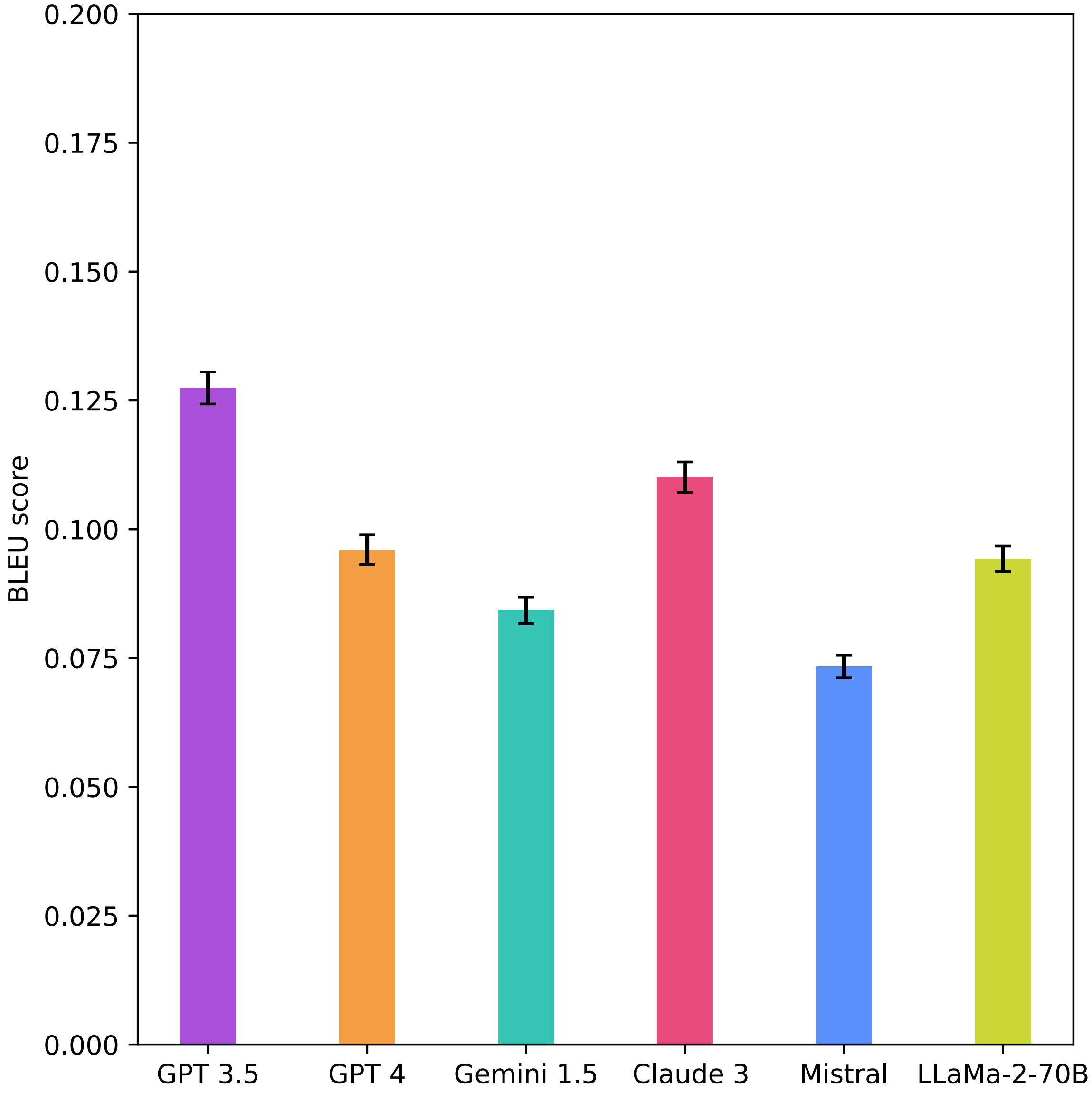

Supplementary Fig. 2. Evaluations of descriptions of 100 gene. The score is BLEU score and higher means better results. Due to model updates, we replace Brad with Gemini 1.5 and Claude 2 with Claude 3. This figure is related to Figure 2.

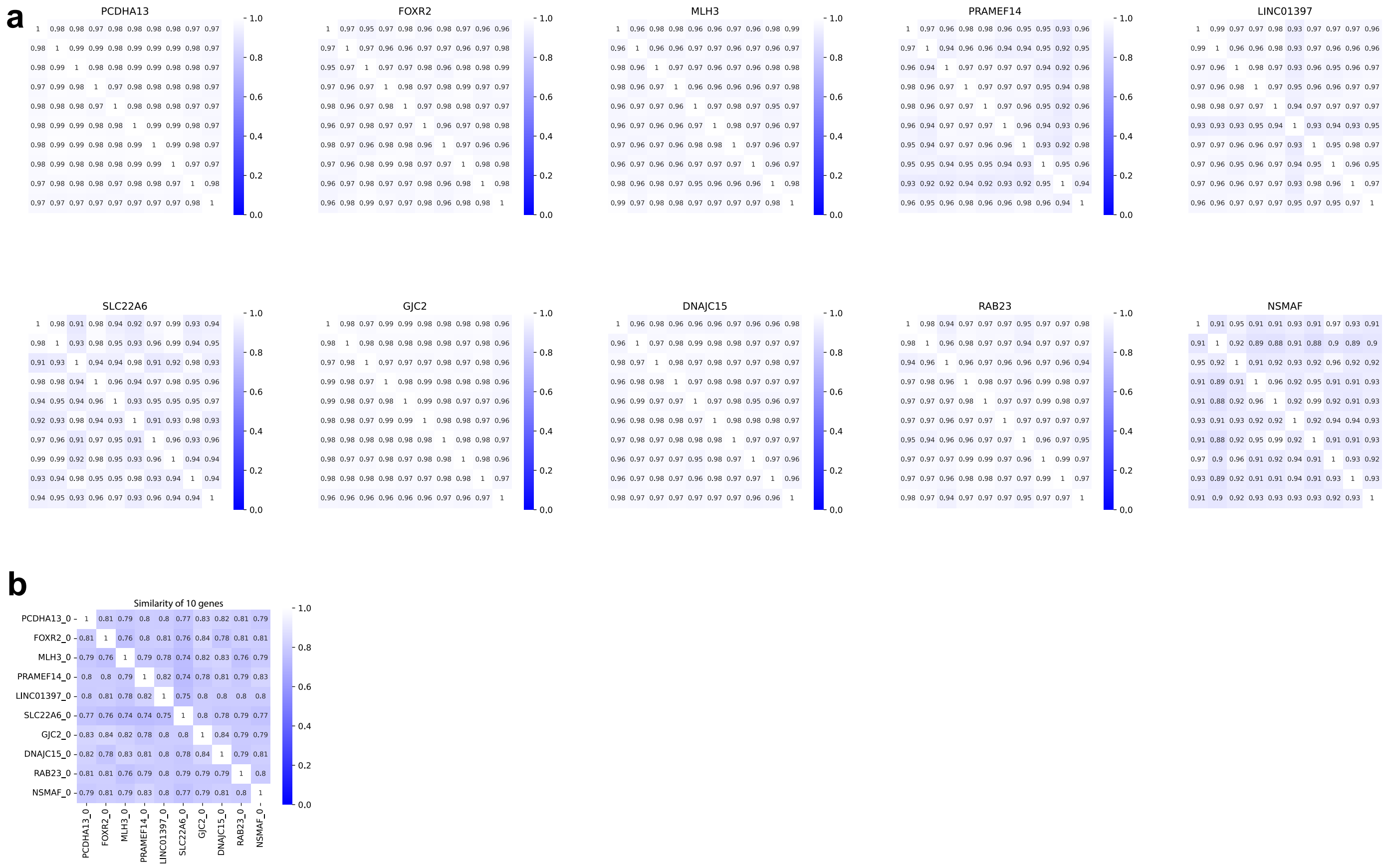

Supplementary Fig. 3. Evaluations of the similarity for embeddings under different conditions. (a) Heatmaps of cosine similarity of embeddings from the same gene under 10 different LLM outputs. We randomly selected 10 genes and generated the LLMs' outputs for these 10 genes based on the same set of prompts. We then computed the embeddings of these outputs and calculated the cosine similarity for these embeddings, hence we have 10 different heatmaps to represent the results for 10 different genes. (b) Heatmap of the cosine similarity of embeddings from 10 different genes. We computed the cosine similarity for the embeddings of different genes. The number 0 represents the index of embeddings we computed based on 10 different LLM outputs. This figure is related to Figure 2.

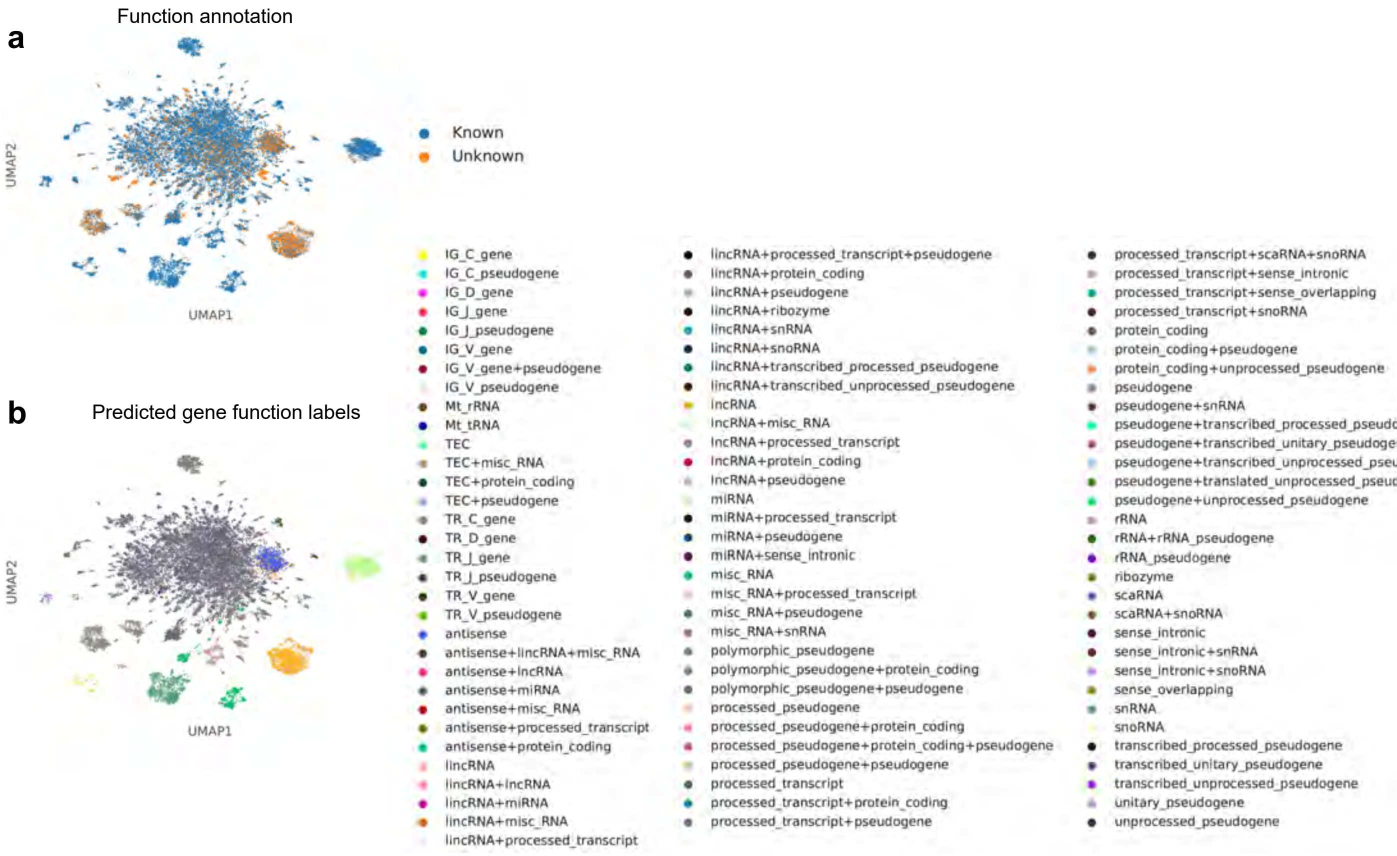

Supplementary Fig. 4. UMAPs for the visualization of gene functional information. (a) UMAPs for the genes with known functional information and unknown functional information. (b) UMAPs for the genes with annotated functional information based on a kNN classifier. For genes with multiple functional annotation, we combined the functions as a new label. This figure is related to Figure 2.

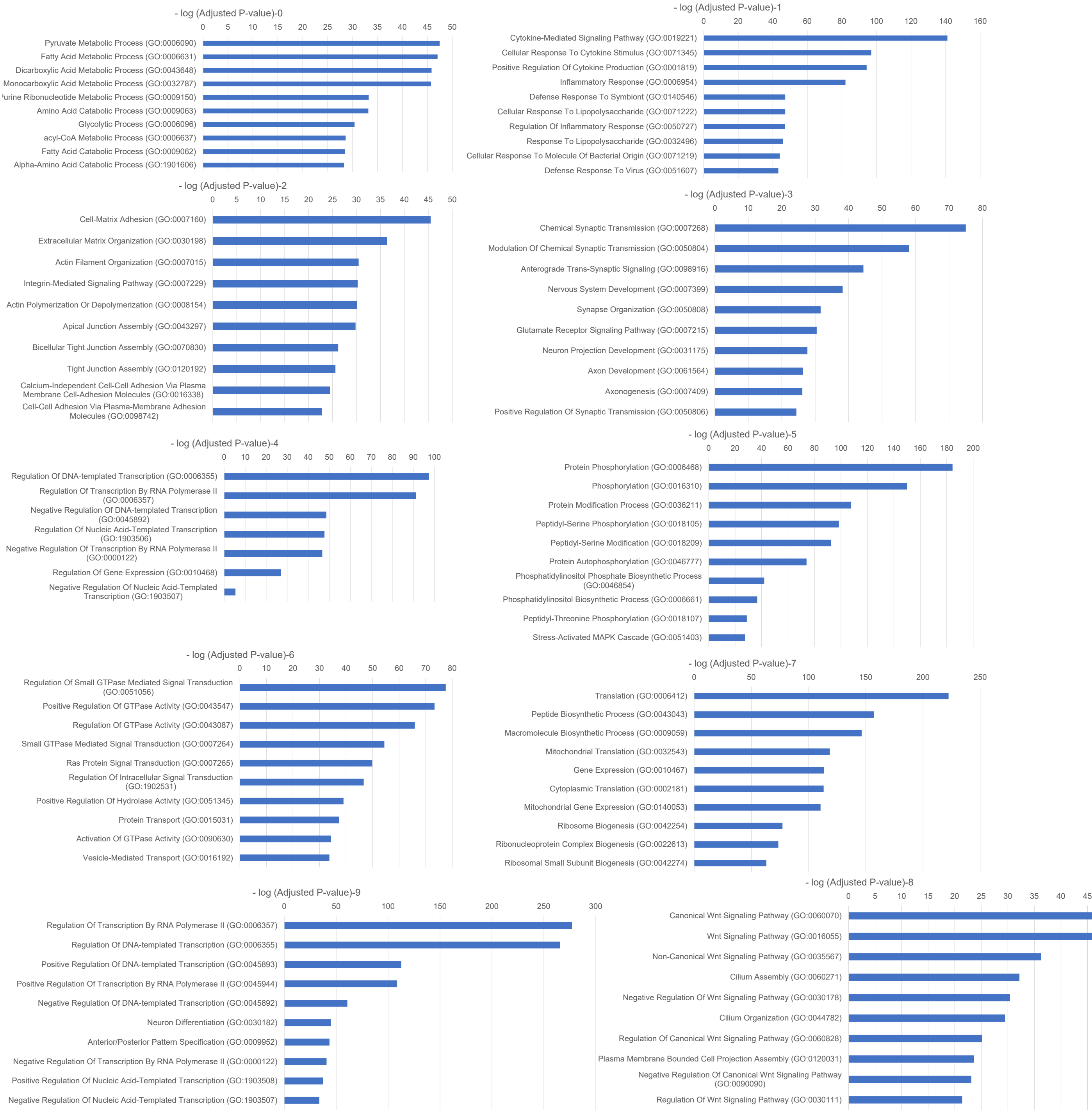

Supplementary Fig. 5. Visualizations of gene pathway information for different clusters based on generated gene embeddings. This figure is related to Figure 2.

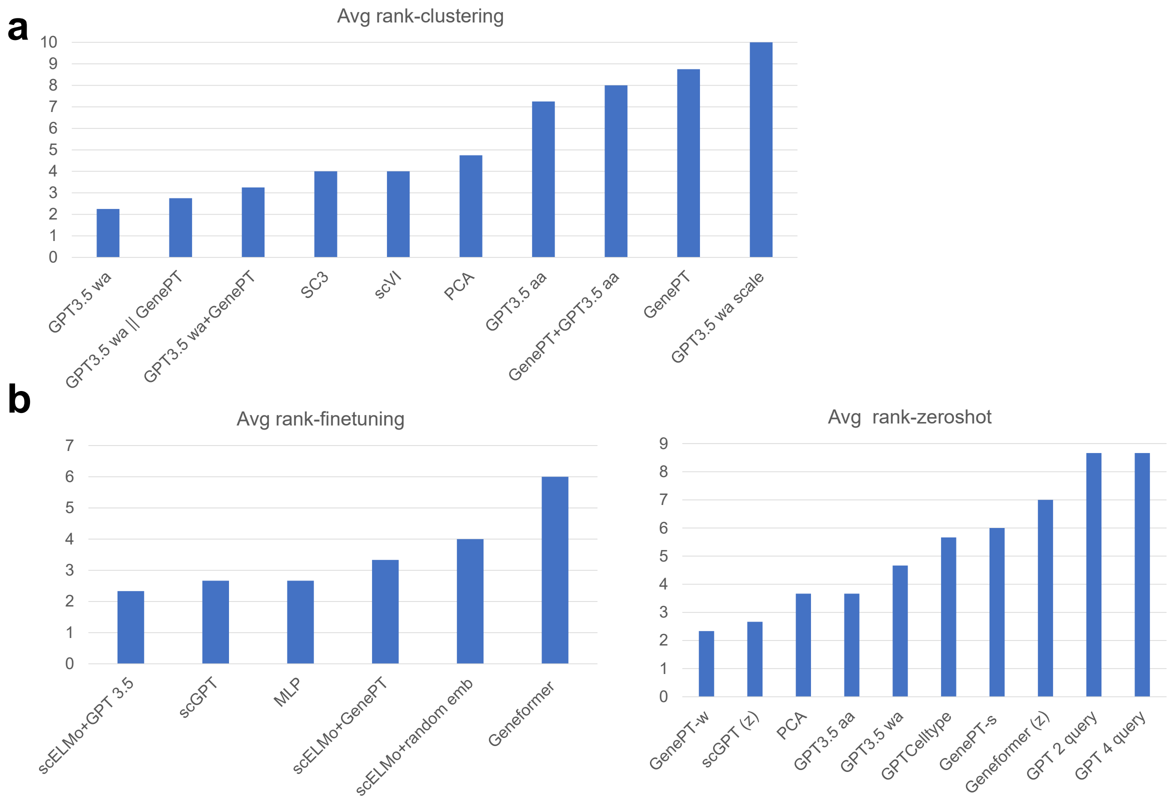

Supplementary Fig. 6. Average ranks for clustering and cell-type annotation. (a) Average-rank information for different methods across datasets. (b) The left panel represents the average-rank information of methods based on fine-tuning for cell-type annotation. The right panel represents the average-rank information of methods based on zero-shot learning for cell-type annotation. This figure is related to Table 1.

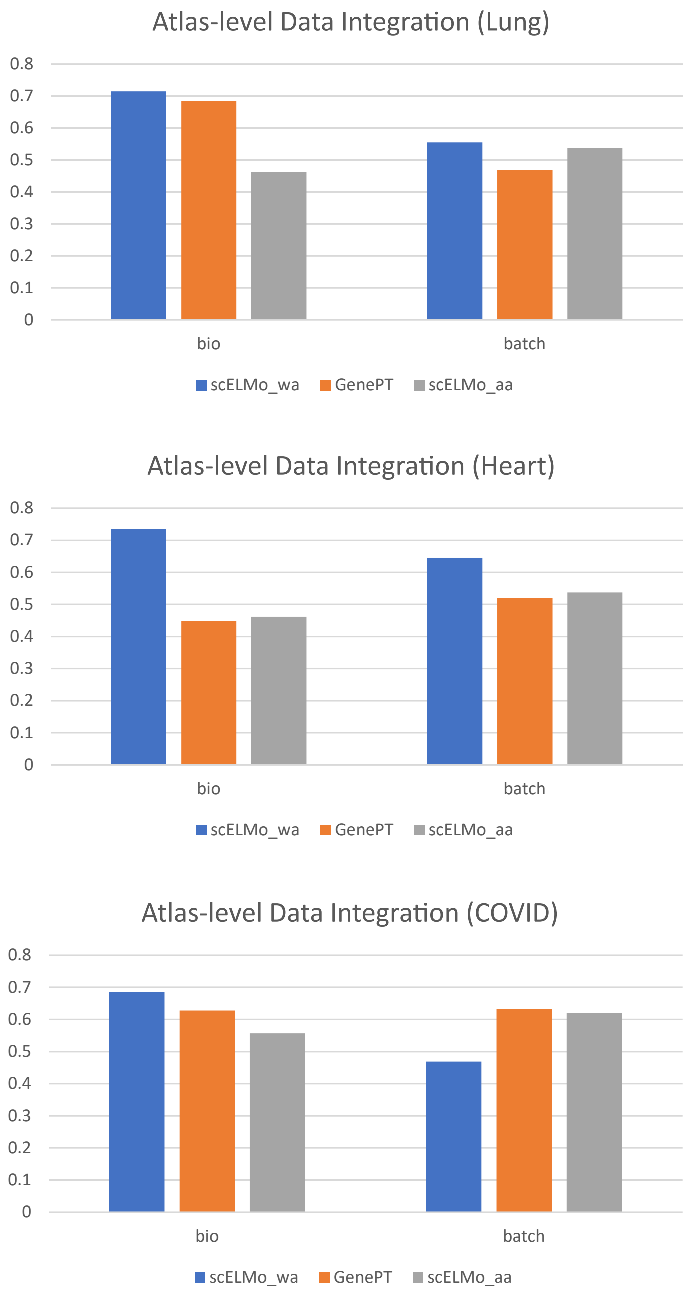

Supplementary Fig. 7. Batch effect correction scores of scELMo and GenePT for atlas-level scRNA-seq dataset. This figure is related to Figure 3.

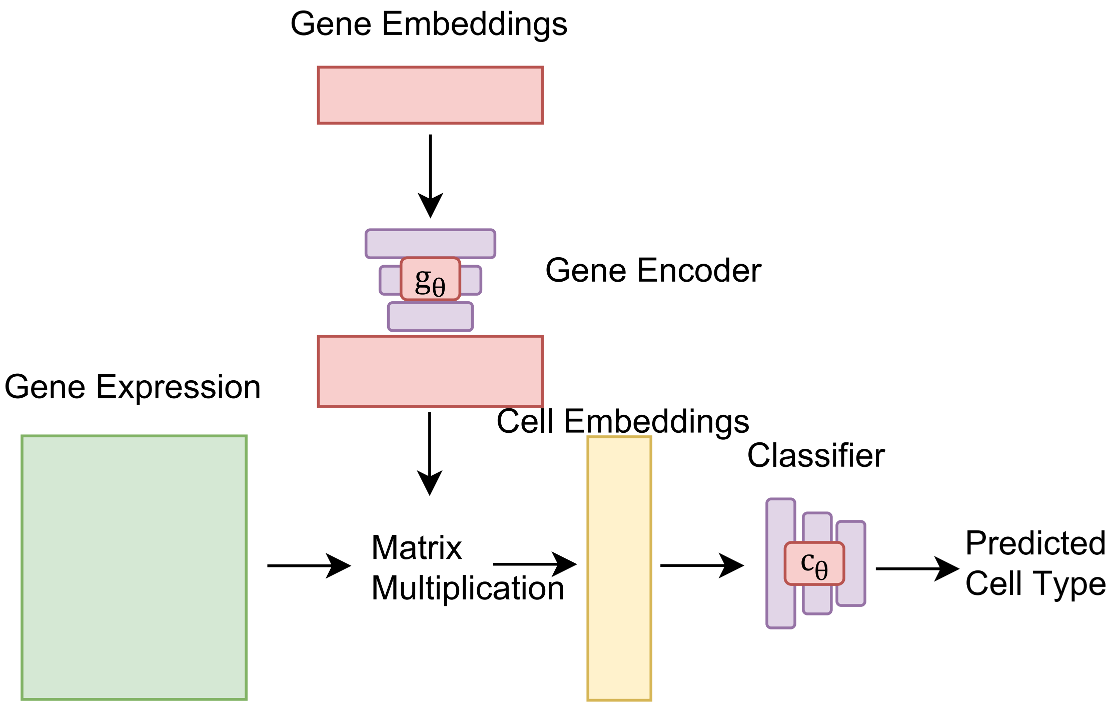

Supplementary Fig. 8. Model architecture of scELMo for learning cell states or disease states. Our workflow contains a gene encoder and a cell-type classifier, which are two neural networks. We train the gene encoder to generate dataset-specific gene embeddings from LLM-generated gene embeddings and multiply it with gene expression profiles to generate dataset-specific cell embeddings, and the classifier make prediction based on the cell embeddings. The whole process is trained with backpropagation. This figure is related to Figure 3.

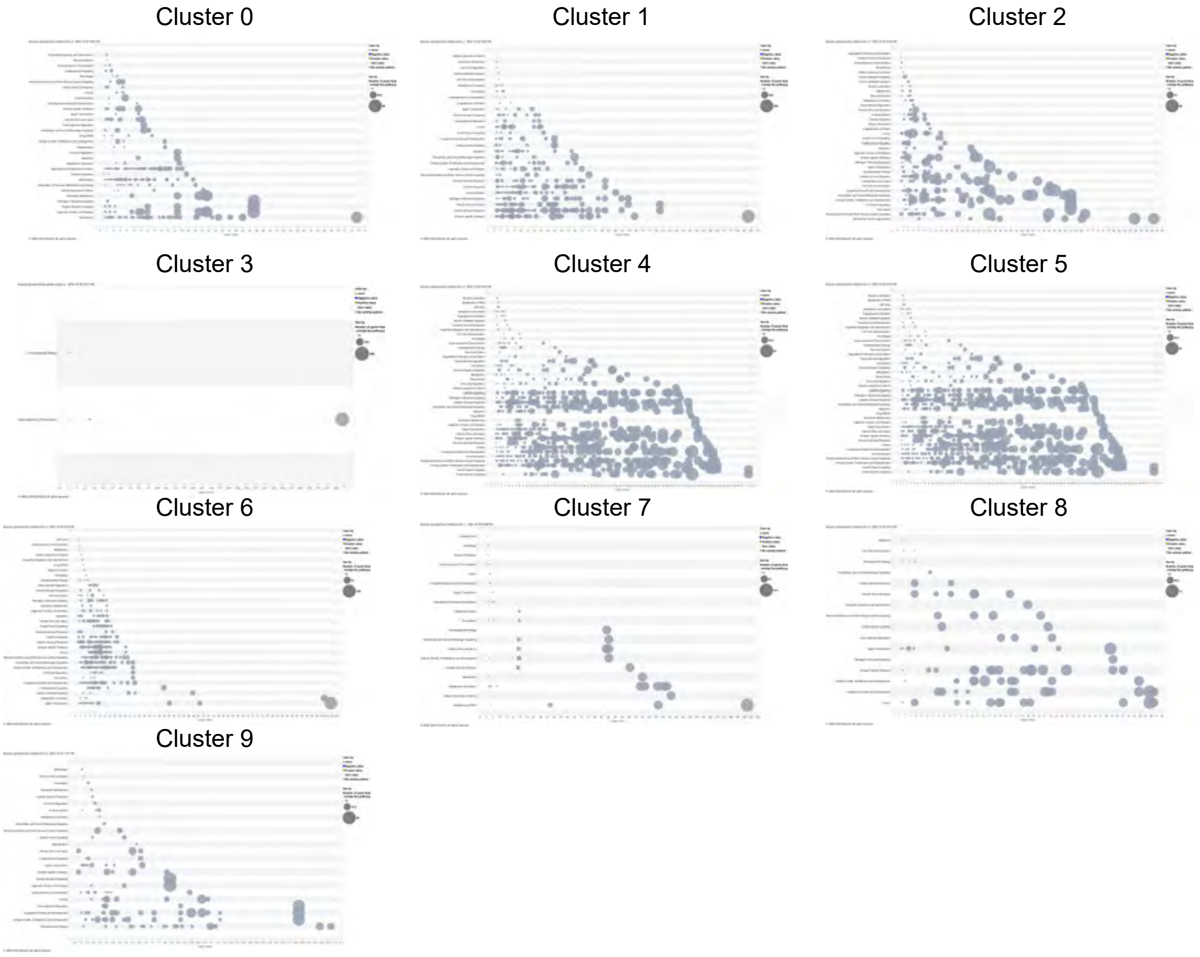

Supplementary Fig. 9. Bubble plots for the pathway information discovered by IPA for each protein-encoding gene cluster. The size of each bubble represents the number of genes in the given pathway. The z-score value can be ignored as we do not incorporate gene expression information. This figure is related to Figure 3.

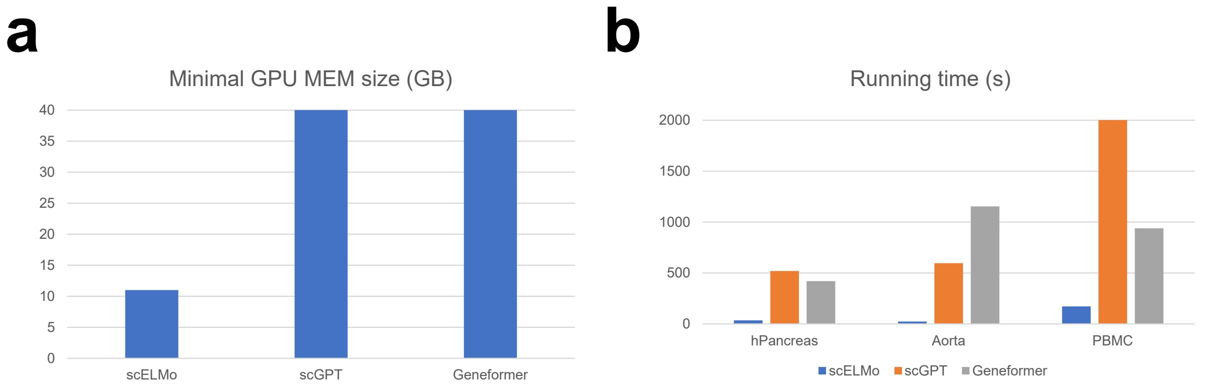

Supplementary Fig. 10. Comparisons of resources. (a) The plot for minimal GPU memory requirements across different FMs. (b) The plot for running time of the cell-type annotation task across different FMs. This figure is related to Figure 2-5.

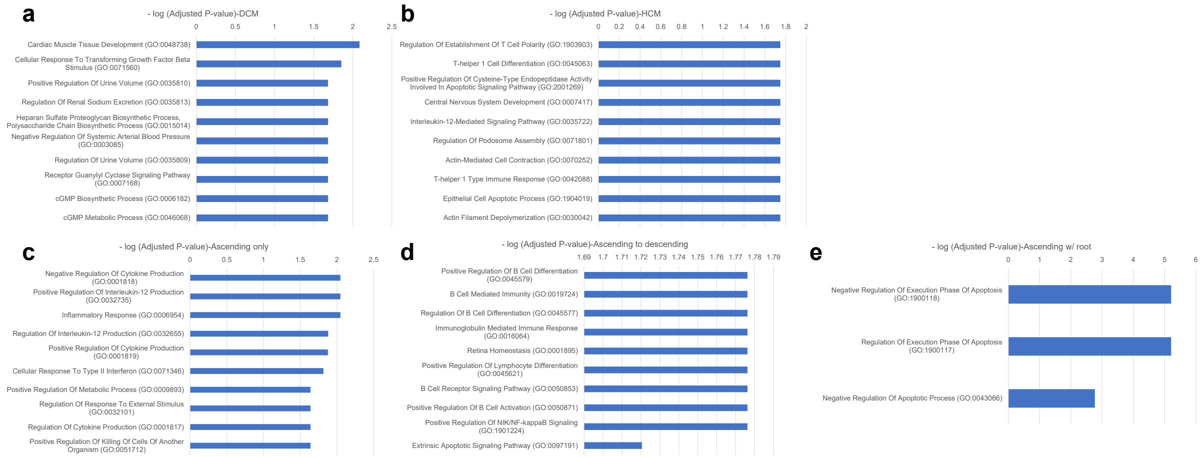

Supplementary Fig. 11. Visualizations of gene pathway information for different conditions based on selected genes. (a) The GO enrichment results of target therapies for DCM. (b) The GO enrichment results of target therapies for HCM. (c) The GO enrichment results of target therapies for Ascending only. (d) The GO enrichment results of target therapies for Ascending to descending. (e) The GO enrichment results of target therapies for Ascending w/ root. This figure is related to Figure 4.

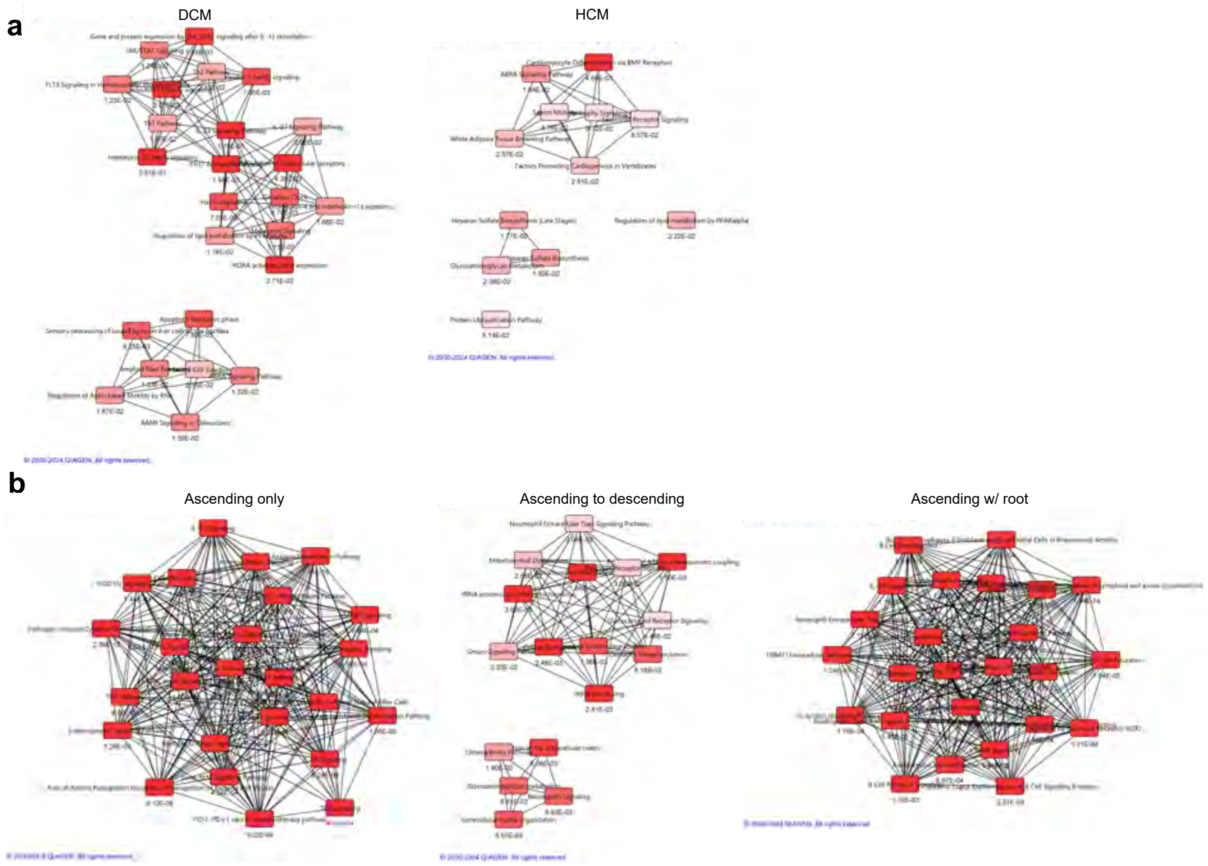

Supplementary Fig. 12. Visualizations of IPA information for different conditions based on selected genes for in-silico treatment analysis. (a) The pathway information from the Heart dataset. (b) The pathway information from the Aorta dataset. The line represents pathway interaction, and we also show the p-value for each selected pathway. This figure is related to Figure 4.

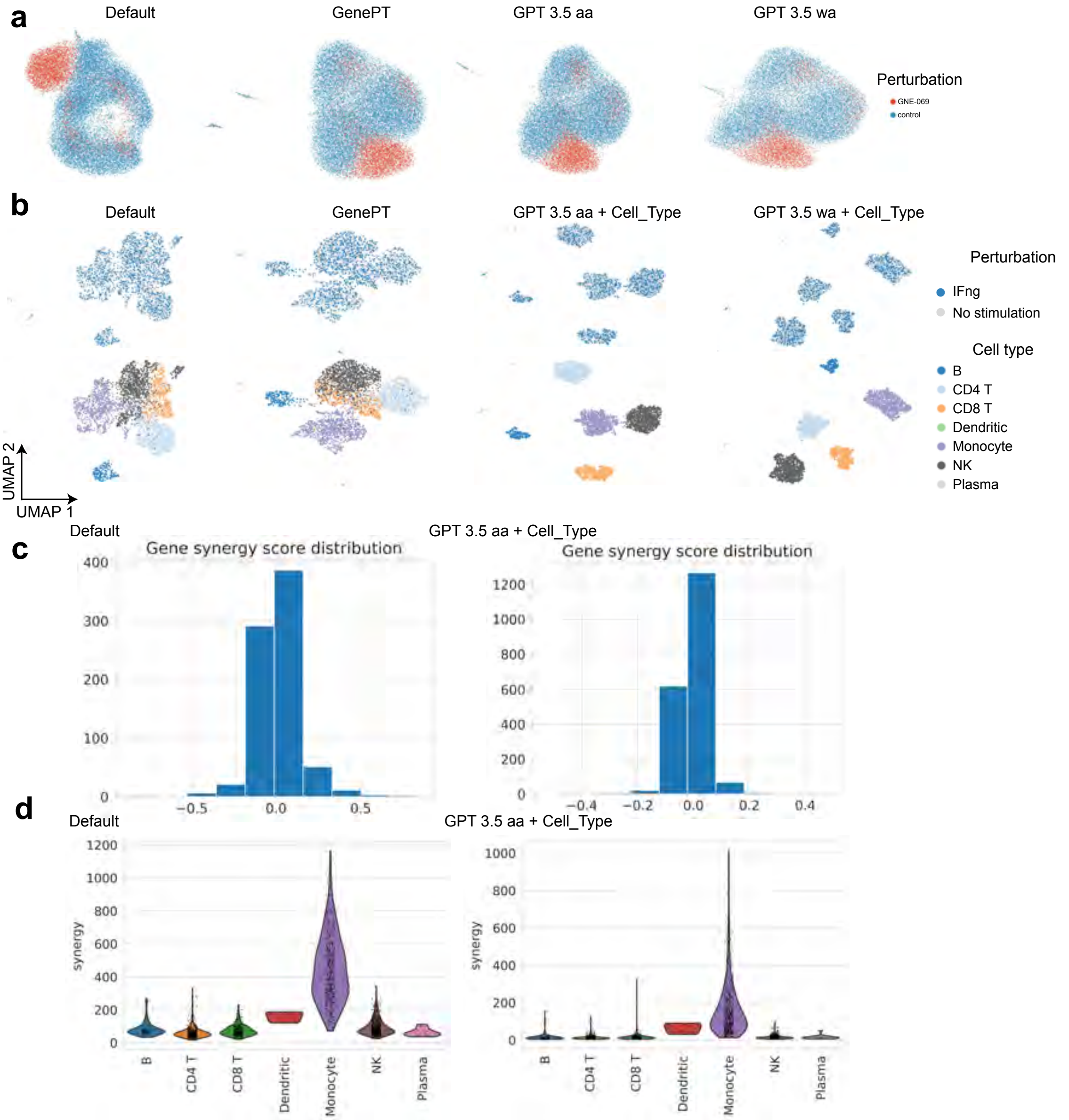

Supplementary Fig. 13. UMAPs for the results of CINEMA-OT under different input settings and datasets. (a) UMAPs visualization for the confounder space of CINEMA-OT under different methods based on the ChangYe2021 dataset. All cells in this dataset have the same cell type. (b) UMAPs visualization for the confounder space of CINEMA-OT under different methods based on perturbed PBMC dataset. The labels for the UMAPs include perturbation conditions (upper panel) and cell types (lower panel). (c) Plots for the gene synergy score distribution, labelled by different methods. (d) Plots for the gene synergy distribution across cell types, labelled by different methods. This figure is related to Figure 5.

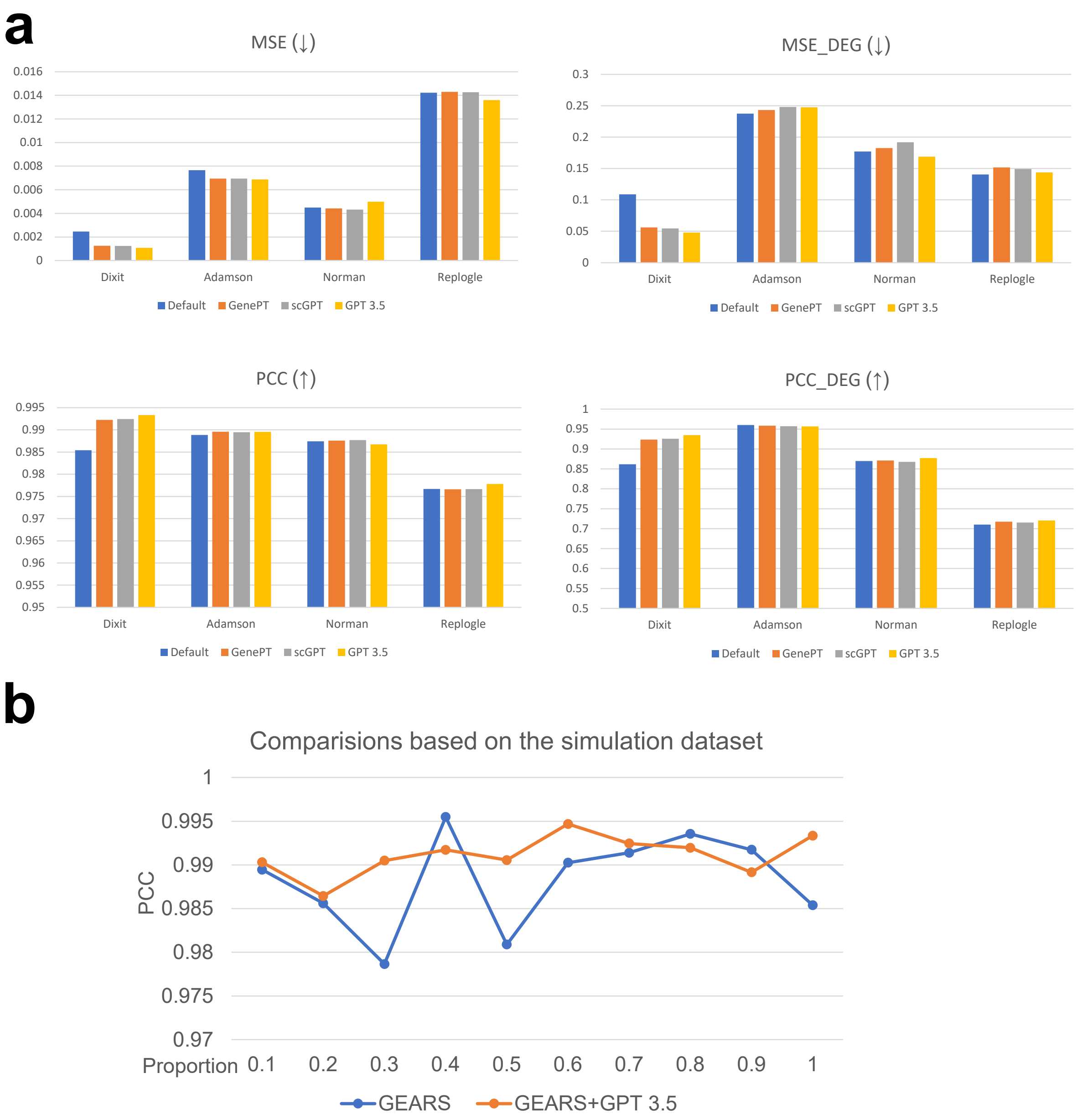

Supplementary Fig. 14. The results of perturbation prediction for all datasets. (a) The MSE, MSE_DEG, PCC and PCC_DEG of all benchmarked methods across all datasets. The direction of the arrow represents the direction of better results. (b) Prediction results under the simulation dataset by subsetting the Dixit dataset with different cell-level proportions. This figure is related to Figure 5.

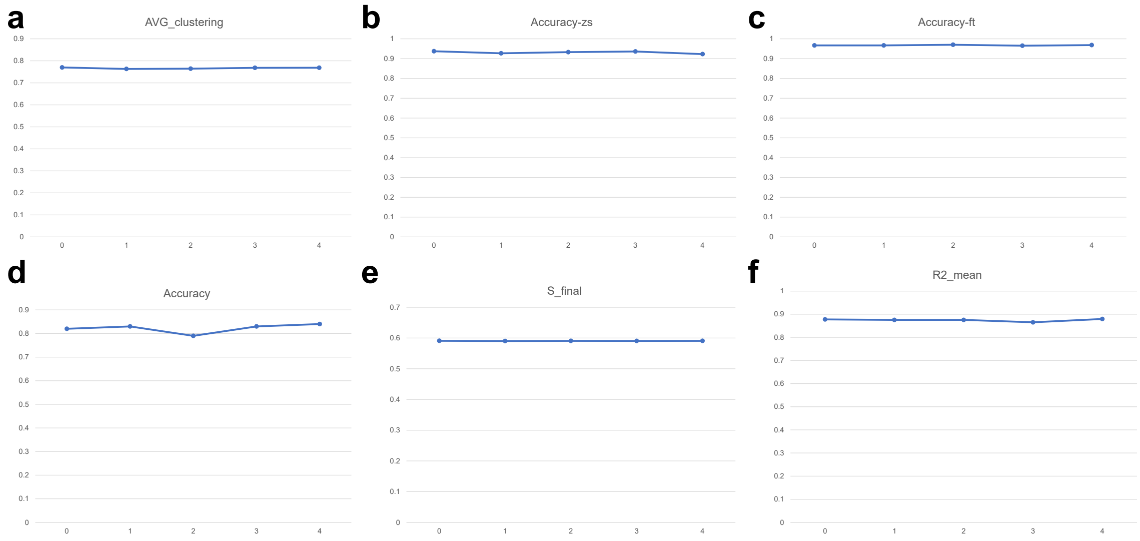

Supplementary Fig. 15. Results of all downstream applications with gene embeddings from different random seeds 0-4. Here we used different random seeds to generate the descriptions of genes. (a) Results of clustering metric based on hPancreas-train dataset. (b) Results of classification metric based on the hPancreas dataset with zero-shot framework. (c) Results of classification metric based on the hPancreas dataset with fine-tuning framework. (d) Results of classification metric based on the Heart dataset for in-silico treatment analysis. (e) Results of integration metric based on the Cytof-CITE-seq dataset for batch effect correction. (f) Results of regression metric based on the CPA example dataset for perturbation prediction. We record the average R2 scores of each seed and display them. This figure is related to Figure 5.

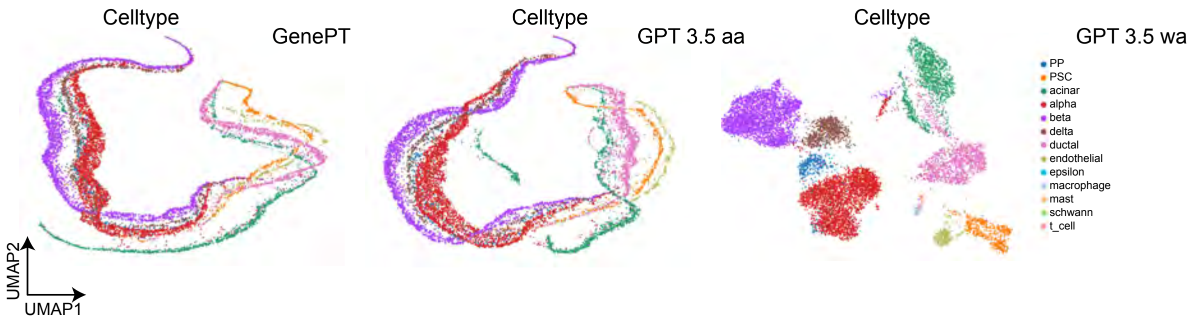

Supplementary Fig. 16. UMAPs for cell embeddings with different sources based on the hPancreas-train dataset. Each panel is colored by the cell types. (a) UMAPs for the cell embeddings based on GenePT. (b) UMAPS for the cell embeddings based on aa mode. (c) UMAPs for the cell embeddings based on wa mode. This figure is related to Figure 5.

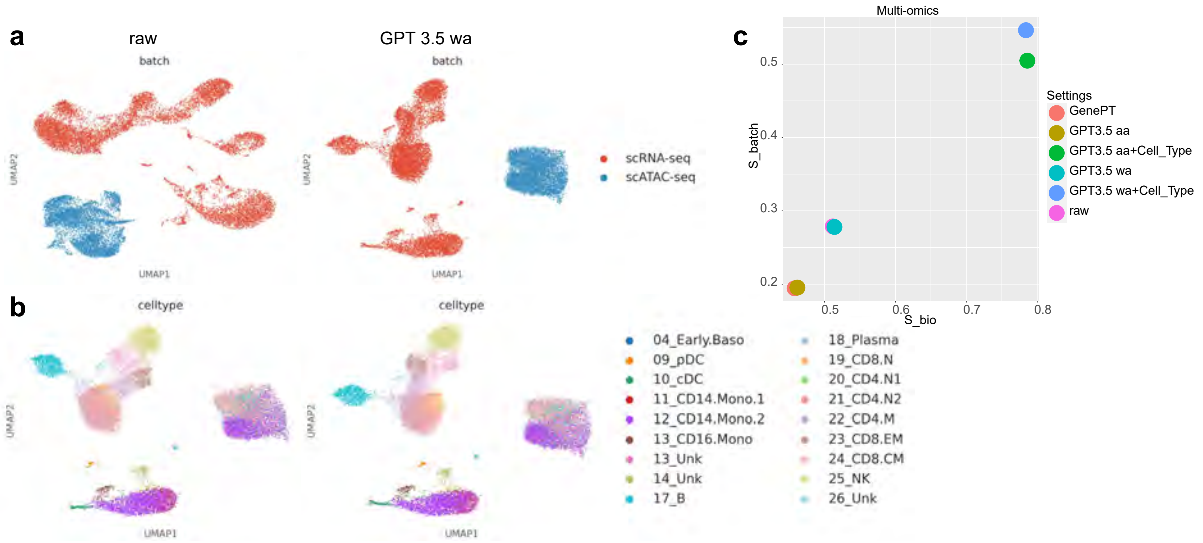

Supplementary Fig. 17. Results of batch effect correction for multi-omic data (scATAC-seq, scRNA-seq). (a) UMAPs of batch information for the raw data and cell embeddings from GPT 3.5 wa mode. (b) UMAPs of cell-type information for the raw data and cell embeddings from GPT 3.5 wa mode. (c) Evaluations of the batch effect correction for multi-omic datasets across different methods. This figure is related to Figure 5.

a

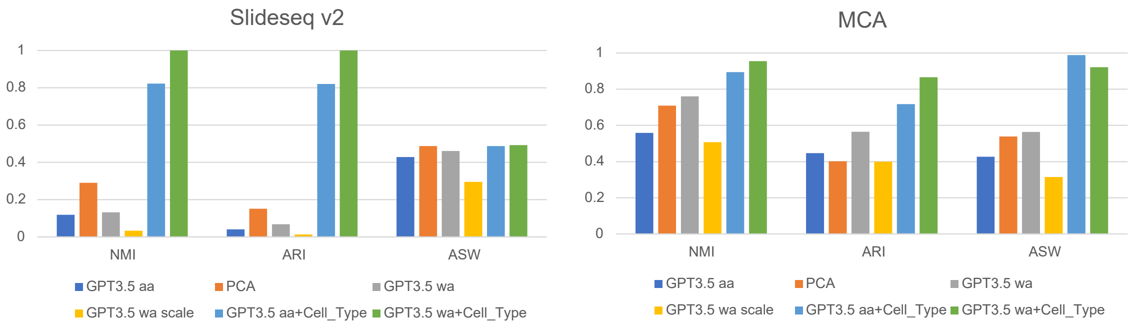

b

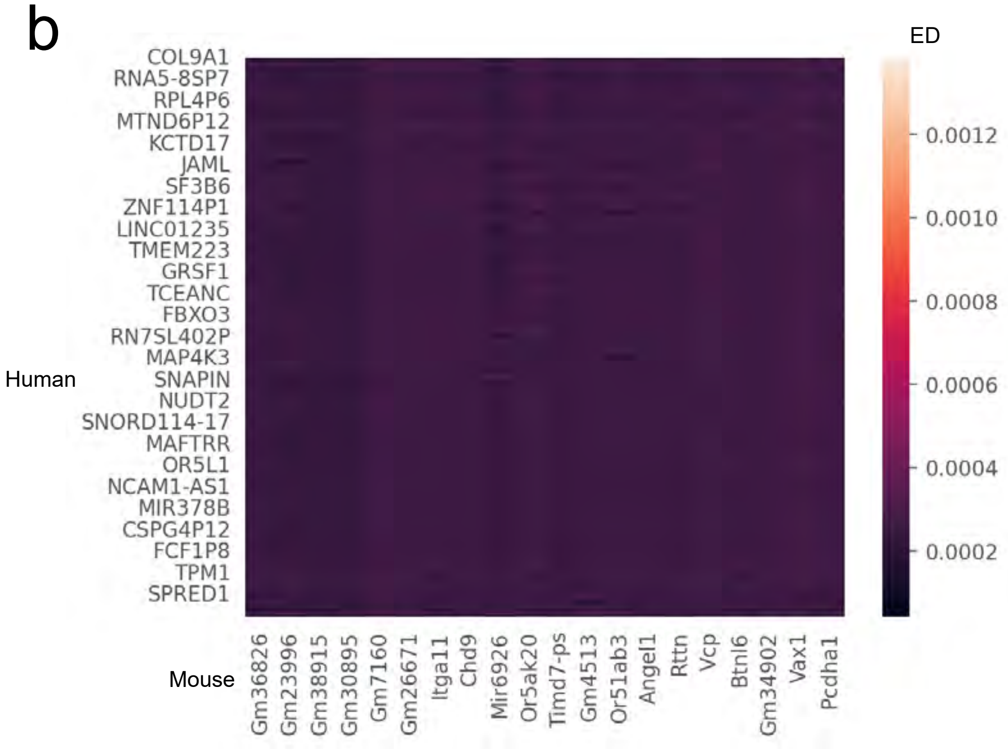

Supplementary Fig. 18. Results of our exploration for gene embeddings from Mouse. (a) Clustering performance for Mouse data. The left panel represents the clustering metrics based on Slide-seq v2 data. The right panel represents the clustering metrics based on MCA data. (b) A heatmap for gene-gene interaction colored by the value of ED. This figure is related to Figure 5.

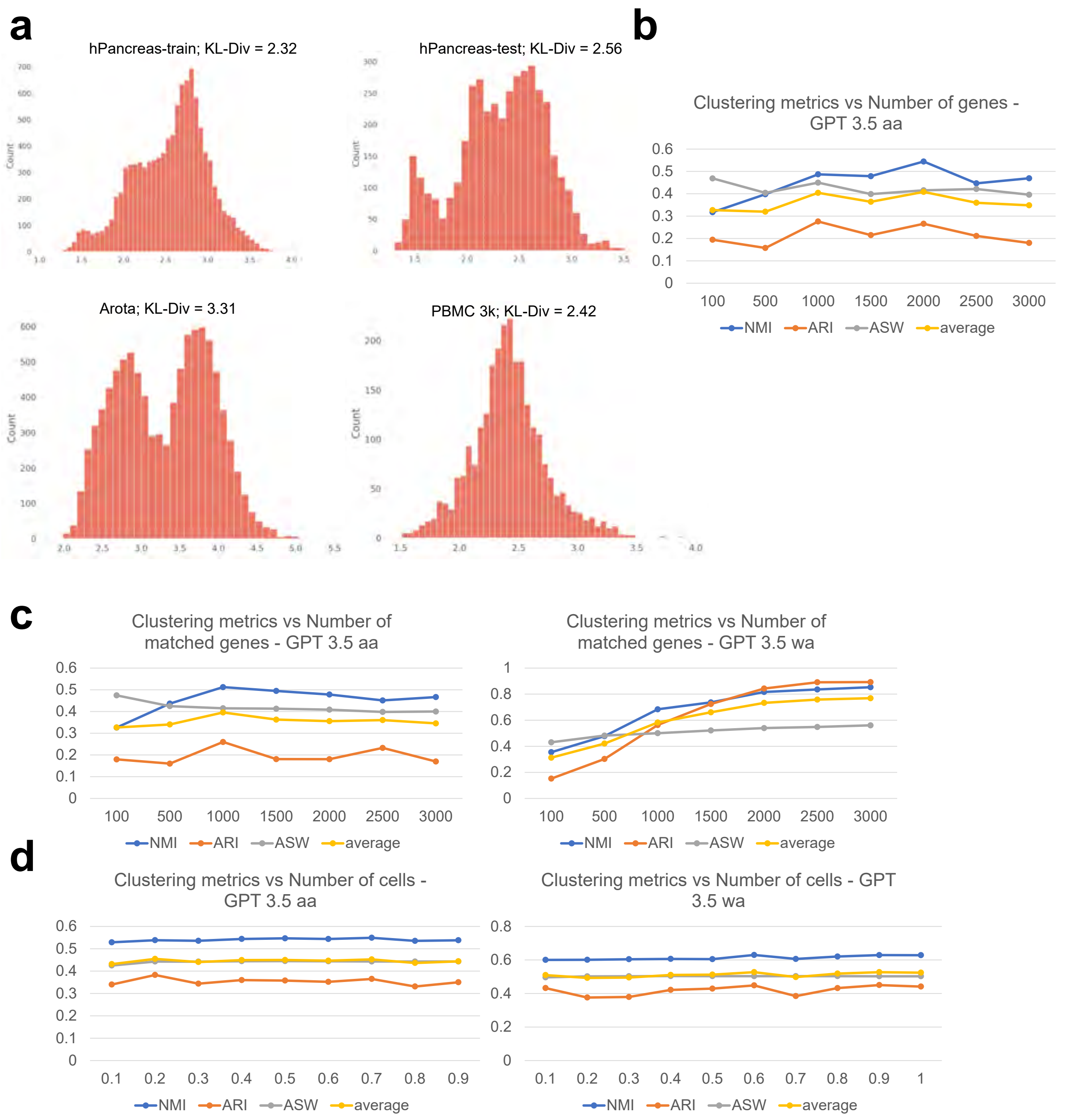

Supplementary Fig. 19. Results of our exploration for the cell clustering task. (a) KL-Div for the two distributions across different datasets. (b) The relation between the number of recorded genes and clustering metrics is based on the GPT 3.5 aa mode. (c) The relation between the number of matched genes and clustering metrics is based on the GPT 3.5 aa mode (left panel) and the GPT 3.5 wa mode (right panel). (d) The relation between the proportion of cells and clustering metrics is based on the GPT 3.5 aa mode (left panel) and the GPT 3.5 wa mode (right panel). This figure is related to Figure 2-5.

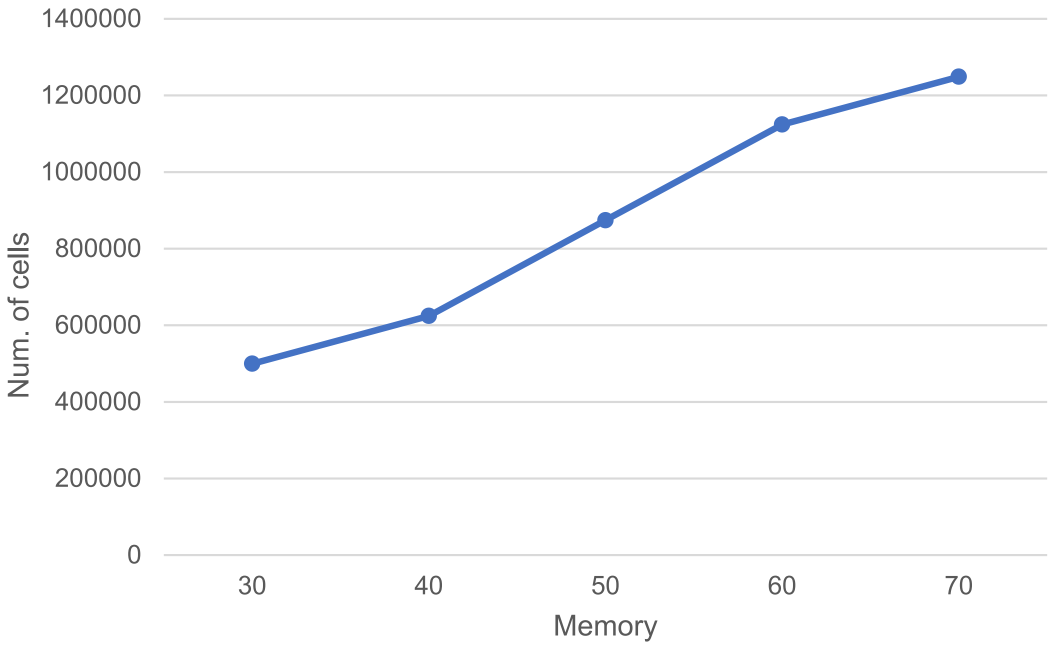

Supplementary Fig. 20. The relationship between memory usage and its corresponding peak number of cells. This figure is related to Figure 2-5.

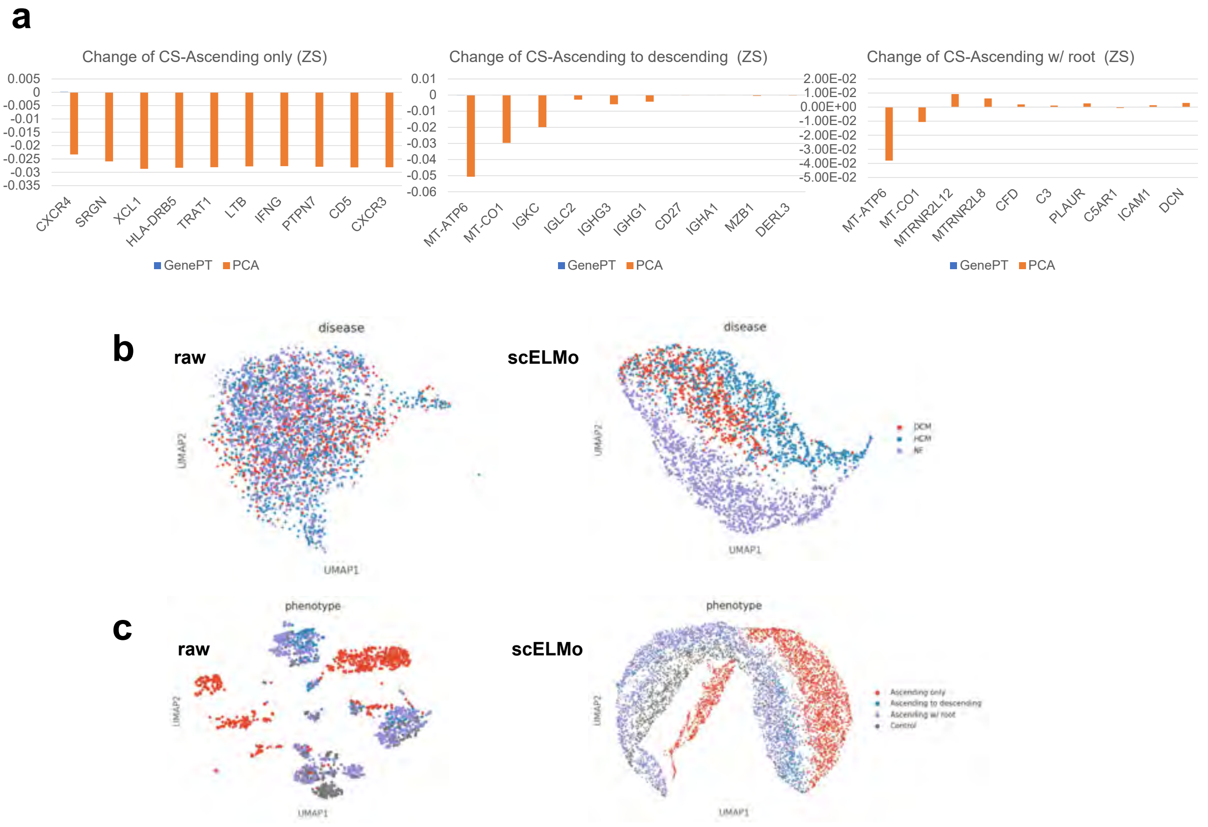

Supplementary Fig. 21. Change of CS under the zero-shot (ZS) learning framework and UMAPs for visualization. (a) The change of CS based on cell embeddings from GenePT or PCA for the Aorta dataset. We considered all three different disease states. (b) UMAPs visualization for the original gene expression space (left panel) and cell embeddings from finetuned scELMo (right panel) based on the Heart dataset. Figures are colored by cell conditions. (c) UMAPs visualization for the original gene expression space (left panel) and cell embeddings from finetuned scELMo (right panel) based on the Aorta dataset. Figures are colored by cell conditions. This figure is related to Figure 2-5.

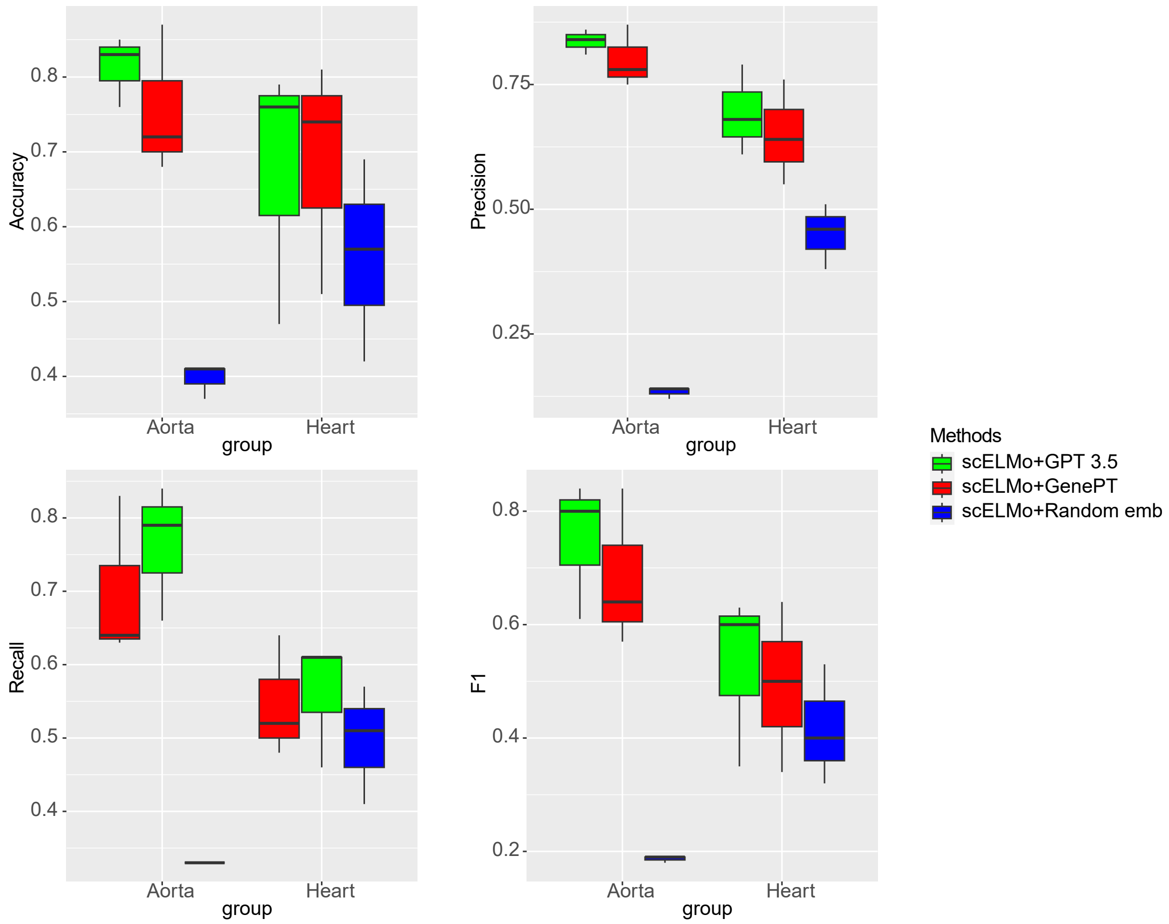

Supplementary Fig. 22. Metrics for disease classification under different gene embeddings across the two datasets. Different panels represent values from different metrics, and we have four metrics in this task. This figure is related to Figure 4.

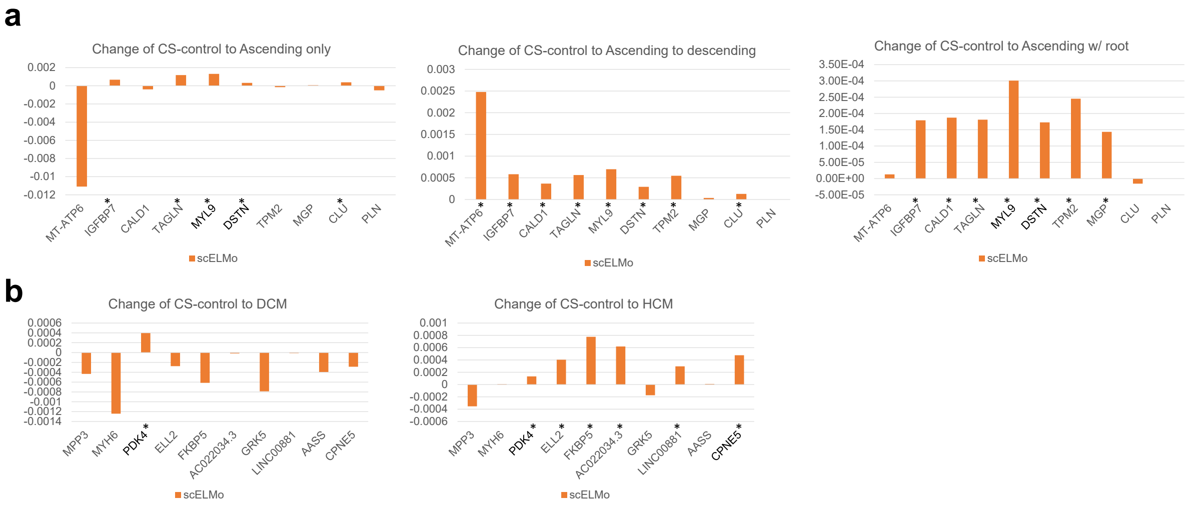

Supplementary Fig. 23. Change of CS for silencing DEGs in the control case. (a) The change of CS based on cell embeddings from scELMo for the Aorta dataset. (b) The change of CS based on the cell embeddings from scELMo for the Heart dataset. We highlighted the genes detected by both GenePT and scELMo using stars (*) and marked the genes that were discovered by previous research as genes related to disease pathway using **bold** type. This figure is related to Figure 4.

Supplementary Note 1: Examples of the text description outputs of different LLMs.

In this section, we present the differences between the text description from NCBI and the text description from GPT 3.5 for the same gene. We highlight the problematic output information of each text using red color, the description of functional information of each text using blue color, and the specific information contained in each text using **bold** text.

Here is an example for the text description of gene COL1A1 from NCBI:

Official Symbol COL1A1provided by HGNC Official Full Name collagen type I alpha 1 chain-provided by HGNC Primary source HGNC:HGNC:2197 See related ***Ensembl:ENSG00000108821 MIM:120150; AllianceGenome:HGNC:2197 Gene type protein coding RefSeq status REVIEWED Organism Homo sapiens Lineage Eukaryota; Metazoa; Chordata; Craniata; Vertebrata; Euteleostomi; Mammalia; Eutheria; Euarchontoglires; Primates; Haplorrhini; Catarrhini; Hominidae; Homo Also known as OI1; OI2; OI3; OI4; EDSC; CAFYD; EDSARTH1*** *Summary This gene encodes the pro-alpha1 chains of type I collagen whose triple helix comprises two alpha1 chains and one alpha2 chain. Type I is a fibril-forming collagen found in most connective tissues and is abundant in bone, cornea, dermis and tendon. Mutations in this gene are associated with osteogenesis imperfecta types I-IV, Ehlers-Danlos syndrome type VIIA, Ehlers-Danlos syndrome Classical type, Caffey Disease and idiopathic osteoporosis. Reciprocal translocations between chromosomes 17 and 22, where this gene and the gene for platelet-derived growth factor beta are located, are associated with a particular type of skin tumor called dermatofibrosarcoma protuberans, resulting from unregulated expression of the growth factor. Two transcripts, resulting from the use of alternate polyadenylation signals, have been identified for this gene. [provided by R. Dalgleish, Feb 2008] Expression Biased expression in gall bladder (RPKM 850.7), urinary bladder (RPKM 497.1) and 11 other tissues See more Orthologs mouse all.*

Here is an example for the text description of gene COL1A1 from GPT 3.5 using the prompt: *Please summarize the major function of a gene: COL1A1. Use academic language in one paragraph and include pathway information.*:

COL1A1 is a gene that encodes the alpha1 chain of type I collagen, ***which is the most abundant extracellular matrix protein and a major component of the bone, skin, tendon, and other connective tissues. The gene plays a crucial role in the synthesis and assembly of type I collagen fibers, contributing to the structural integrity and mechanical properties of these tissues. Additionally, type I collagen is involved in the regulation of signaling pathways, such as the TGF-β signaling pathway, which plays a key role in the regulation of cell growth, differentiation, and extracellular matrix synthesis. Mutations in COL1A1 are associated with various connective tissue disorders, including osteogenesis imperfecta and Ehlers-Danlos syndrome. Therefore, understanding the function of COL1A1 is important in elucidating the pathogenesis of these disorders and developing potential therapeutic strategies.***

For the text description from NCBI, we need to clean the format of the original data and transfer them into text representations. Therefore, the sentences and words in this text are incoherent and their format does not strictly follow the grammar. Such difference poses the problem of model alignment with human value. Moreover, text descriptions of NCBI focus specifically on the variety of gene names, symbols, refseq status, and other properties. Moreover, it contained detailed functional information of the given gene. Some of the information may be redundant. One advantage of the text representation from NCBI is its authority, which means the reliability of text descriptions from NCBI is generally greater than the outputs from LLMs for researchers.

For the text description from GPT 3.5, we can see that the sentences and words are coherent and formalized under the correct grammar framework. This text focuses more on the overview of functions for this given gene, including its major functional tissues and cell types (for cell-type marker genes like CD79). Moreover, this text also includes the relation between COLA1A1 and certain diseases, highlighting the potential of this gene as a therapeutic target. Such prior information will be helpful in the in-silico treatment analysis research. Moreover, different prompts can generate different types of descriptions for the same gene, exploring the diversity of the outputs from LLMs is also an interesting research track.

Therefore, these two different types of text descriptions have their own advantages and disadvantages, which might be the explanation for their different suitable scenarios. One interesting research topic will be how to combine the advantages of these two kinds of text descriptions to enhance their downstream applications.

Here is an example for the text description of B cells from GPT 3.5 using the prompt: *Please summarize the major function of cell type: B. Use academic language in one paragraph.*:

B cells, a crucial component of the adaptive immune system, play a pivotal role in ***humoral immunity by generating antibodies (immunoglobulins) in response to the recognition of specific antigens. Following activation, B cells undergo clonal expansion and differentiation into plasma cells, which are specialized antibody-producing cells. The antibodies secreted by plasma cells serve as effectors in immune responses, neutralizing pathogens, facilitating their removal by phagocytic cells, and promoting various immune processes such as opsonization and complement activation. Additionally, B cells can differentiate into memory B cells, which confer long-term immunity by enabling a rapid***

and robust antibody response upon re-exposure to the same pathogen. Overall, B cells are central to the adaptive immune system's ability to recognize and respond to a wide array of infectious agents and contribute significantly to immune memory and protection.

For the text description from GPT 3.5, we find that the text description is also coherent and follows the standard grammar. It summarizes the major functions of B cells as well as the cell-cell communication that B cells involve.

Supplementary Note 2: Analyzing mouse single-cell data.

In this section, we explored the contribution of LLM embeddings for analyzing omic data from Mouse. We first analyzed the ability of clustering in Figure 18 (a) for both single-cell data and spatial transcriptomic data. While gene embeddings from GPT 3.5 wa mode can generate clustering performance better than PCA for scRNA-seq data (MCA)[120], such embeddings cannot improve the clustering for spatial data sequenced by Slide-seq v2[121]. Moreover, cell-type embeddings from GPT 3.5 can also carry information from Mouse, proved by high scores for evaluating clustering performance.

We also considered the cross-species analysis for the similarity of embeddings from Human genes and embeddings from Mouse genes, which can enrich the study of gene similarity in addition to orthologous genes. We computed the Euclidean distance (ED) for all gene pairs based on gene embeddings from Mouse and Human, shown in Figure 18 (b). We then ranked the ED to retrieve most similar genes. To verify the similarity, we figured out that the closet gene pairs were the orthologous genes, thus the gene embeddings from LLMs could also reflect the common information from two species.

Supplementary Note 3: Understanding the effect of the number of cells and the number of genes towards GenePT and scELMo.

In this section, we further investigated the difference between the two averaging modes and the application scenarios and analyzed the relationship between the attributes of raw data and clustering effects.

First, we directly plotted the visualization results of three different methods for the hPancreas-train dataset in Figure S16. From this figure, we found that using neither gene embeddings from GenePT nor GPT 3.5 with the aa mode could preserve the cell-type-specific clusters in the space of UMAPs. However, using gene embeddings from GPT 3.5 with wa mode could preserve the major cell-type-specific clustering information. Based on this interesting observation, we further compared the weights used for these two averaging modes based on KL-divergence (KL-Div)[45], which could reflect the difference for the distribution of gene expression levels in each cell and a uniform distribution with $P = \frac{1}{m}$. For each dataset, the row sum is one for both these two cases. Based on Figure S19 (a), we found that for all the four datasets we compared in the cell clustering task, the KL-div was larger than two and none of them had cells with zero divergence. Therefore, the distribution of gene expression levels carried more information compared with the weights based on uniform distribution. Moreover, we also showed that using

wa mode was better for batch effect correction. Therefore, the wa mode is more suitable to handle tasks under the zero-shot learning framework.

Second, we analyzed the relation between the clustering performance and the number of genes. The first scenario we intended to investigate is the relation between the number of recorded genes and the clustering performance. In Figure S19 (b), we display the change of clustering metrics with respect to the change of recorded genes based on the hPancreas-train dataset using aa mode. We noticed that the wa mode was not suitable for this research because we might have cells with zero expression by filtering some genes. There is no obvious correlation between the number of recorded genes and the clustering performance. Moreover, since the sources of GenePT or scELMo do not match all of the genes for every scRNA-seq dataset, sometimes we need to fill the gene embeddings of missing genes as zero. Therefore, we also investigated the relation between the number of matched genes and the clustering performance, shown in Figure S19 (c). From this figure, we still did not observe a strong correlation between the number of matched genes and the clustering performance under the aa mode. However, for the wa mode, we found an obvious correlation between these two values. Therefore, having more matched genes can contribute to cell clustering under the wa mode of scELMo. Such conclusion also demonstrates the importance of extending our databases of feature embeddings.

Third, we analyzed the relation between the clustering performance and the number of cells. We subsampled different proportions of cells from the large-scale Onek1k PBMC dataset and computed the clustering results under different numbers of cells. Based on Figure S19 (d), we found that there was no obvious correlation between the number of cells and the clustering performance for Onek1k PBMC dataset. Based on this dataset, we also explore the relationship between memory usage and cell numbers, shown in Figure S20 20. The minimal requirement for loading the Onek1k PBMC dataset is 30 GB, and the growth is linear (O(n) level). Therefore, cell number may not be a factor that can affect the performance of gene embeddings in this task. Moreover, scELMo is also capable of the analysis of large-scale scRNA-seq datasets.

Supplementary Note 4: Analysis of multi-omic data integration.

In this section, we explored the possibility of utilizing gene embeddings from GPT 3.5 to resolve multi-omic data integration task. Here we consider datasets from scRNA-seq and scATAC-seq without paired information. To reduce the dimensions of the scATAC-seq dataset, we transfer the feature information of such dataset from the space of peaks to the space of gene activity scores. The visualization results are summarized in Figures S17 (a) and (b). According to these figures, we can still observe significant batch effect or the difference of cell embeddings from the cells with same cell types. Therefore, the function of scELMo for multi-omic data integration under the zero-shot learning framework is not good. Moreover, based on Figure S17 (c), neither the wa mode nor the aa mode can improve the S_{batch} score and the s_{bio} score significantly. Incorporating the cell-type information into the cell embeddings space can significantly improve the averaged scores, but for metrics like iLISI to evaluate the mixture of batch information, such embeddings still had zero score. Therefore, scELMo is not capable of multi-omic data integration under the zero-shot learning framework.

Supplementary Note 5: The contribution of finetuned model in the in-silico treatment analysis.

In this section, we demonstrated the necessity of using a finetuned model rather than zero-shot learning for in-silico treatment analysis. In Figure S23 (a), we display the change of CS for the same group of DEGs under the ascending aortic aneurysm disease and all genes were not significant for the Ascending only state. Moreover, the change of CS under PCA was nearly constant by varying different genes for removal, and the change of CS was very small for cell embeddings based on GenePT across all three states. Therefore, we concluded that the cell embeddings from either GenePT or PCA were not capable of modeling this disease without the knowledge of the intercellular variability due to diseases. Moreover, based on Figures S23 (b) and (c), such variability was covered by the noise in the original expression space. Therefore, we need to learn a model that can distinguish the cells under different conditions as well as generate representative cell embeddings for the inference of novel therapeutic targets. Our ideas aligned with the strategy adopted by Geneformer.

Moreover, we compared the classification metrics for the cell-level disease condition under different gene embeddings for these two datasets and displayed the results in Figure S22. From this figure, we found using gene embeddings containing information from genes generally had better performance than using embeddings from random numbers. Moreover, the classification results of scELMo based on gene embeddings from GPT 3.5 were slightly better than the results based on gene embeddings from GenePT. Therefore, using gene embeddings from GenePT and GPT 3.5 all contributed to generating representative latent space for different diseases.

We also considered genes whose silence might shift the cell embeddings from the control condition to diseased conditions. Therefore, our candidate genes became DEGs for the control case and we reversed our score to keep its direction (higher score means that the removal of this gene contributes to the change of cells from the healthy condition to diseased conditions). Our results are summarized in Figures S23 (a) and (b). Based on our results, we identified different number of genes for different diseases. Moreover, there existed gene overlap across three states of the given disease, which implied that the removal or silence of such gene might have different contributions for different disease. Because the function of genes is closely related to the pathway[122], our findings can help analyze the pathogenesis of some diseases.

References

1. Wu, J., Yang, S., Zhan, R., Yuan, Y., Chao, L. S., and Wong, D. F. (2025). A Survey on LLM-Generated Text Detection: Necessity, Methods, and Future Directions. Computational Linguistics *51*, 275–338.

2. Zhao, W. X., Zhou, K., Li, J., Tang, T., Wang, X., Hou, Y., Min, Y., Zhang, B., Zhang, J., Dong, Z., et al. (2023). *A survey of large language models*. Preprint at arXiv, https://doi.org/10.48550/arXiv.2303.18223.

3. Zhou, C., Li, Q., Li, C., Yu, J., Liu, Y., Wang, G., Zhang, K., Ji, C., Yan, Q., He, L., et al. (2024). A comprehensive survey on pretrained foundation models: A history from bert to chatgpt. International Journal of Machine Learning and Cybernetics, 1–65.

4. Nguyen, E., Poli, M., Faizi, M., Thomas, A. W., Wornow, M., Birch-Sykes, C., Massaroli, S., Patel, A., Rabideau, C. M., Bengio, Y., et al. (2023). "HyenaDNA: Long-Range Genomic Sequence Modeling at Single Nucleotide Resolution". *Thirty-seventh Conference on Neural Information Processing Systems*.

5. Marin, F. I., Teufel, F., Horlacher, M., Madsen, D., Pultz, D., Winther, O., and Boomsma, W. (2024). "BEND: Benchmarking DNA Language Models on Biologically Meaningful Tasks". *The Twelfth International Conference on Learning Representations*.

6. Fan, Y., Li, Y., Ding, J., and Li, Y. (2024). "GFETM: Genome Foundation-Based Embedded Topic Model for scATAC-seq Modeling". *Research in Computational Molecular Biology*. Ed. by J. Ma. Cham: Springer Nature Switzerland, 314–319. ISBN: 978-1-0716-3989-4.

7. Cui, H., Wang, C., Maan, H., Pang, K., Luo, F., Duan, N., and Wang, B. (2024). scGPT: toward building a foundation model for single-cell multi-omics using generative AI. Nature Methods, 1–11.

8. Theodoris, C. V., Xiao, L., Chopra, A., Chaffin, M. D., Al Sayed, Z. R., Hill, M. C., Mantineo, H., Brydon, E. M., Zeng, Z., Liu, X. S., et al. (2023). Transfer learning enables predictions in network biology. Nature, 1–9.

9. Yang, F., Wang, W., Wang, F., Fang, Y., Tang, D., Huang, J., Lu, H., and Yao, J. (2022). scBERT as a large-scale pretrained deep language model for cell type annotation of single-cell RNA-seq data. Nature Machine Intelligence *4*, 852–866.

10. Han, X., Zhou, Z., Fei, L., Sun, H., Wang, R., Chen, Y., Chen, H., Wang, J., Tang, H., Ge, W., et al. (2020). Construction of a human cell landscape at single-cell level. Nature *581*, 303–309.

11. Saliba, A.-E., Westermann, A. J., Gorski, S. A., and Vogel, J. (2014). Single-cell RNA-seq: advances and future challenges. Nucleic acids research *42*, 8845–8860.

12. Cheung, R. K. and Utz, P. J. (2011). CyTOF—the next generation of cell detection. Nature Reviews Rheumatology *7*, 502–503.

13. Stoeckius, M., Hafemeister, C., Stephenson, W., Houck-Loomis, B., Chattopadhyay, P. K., Swerdlow, H., Satija, R., and Smibert, P. (2017). Simultaneous epitope and transcriptome measurement in single cells. Nature methods *14*, 865–868.

14. Karemaker, I. D. and Vermeulen, M. (2018). Single-cell DNA methylation profiling: technologies and biological applications. Trends in biotechnology *36*, 952–965.

15. Hao, M., Gong, J., Zeng, X., Liu, C., Guo, Y., Cheng, X., Wang, T., Ma, J., Zhang, X., and Song, L. (2024). Large-scale foundation model on single-cell transcriptomics. Nature methods *21*, 1481–1491.

16. Liu, T., Li, K., Wang, Y., Li, H., and Zhao, H. (2023). *Evaluating the Utilities of Foundation Models in Single-cell Data Analysis*. Preprint at BioRxiv, https://doi.org/10.1101/2023.09.08.555192.

17. Kedzierska, K. Z., Crawford, L., Amini, A. P., and Lu, A. X. (2025). Zero-shot evaluation reveals limitations of single-cell foundation models. Genome Biology *26*, 101.

18. Chen, Y. and Zou, J. (2025). Simple and effective embedding model for single-cell biology built from chatgpt. Nature biomedical engineering *9*, 483–493.

19. Wheeler, D. L., Barrett, T., Benson, D. A., Bryant, S. H., Canese, K., Chetvernin, V., Church, D. M., DiCuccio, M., Edgar, R., Federhen, S., et al. (2007). Database resources of the national center for biotechnology information. Nucleic acids research *35*, D5–D12.

20. Shevlane, T. (n.d.). "Structured Access: An Emerging Paradigm for Safe AI Deployment". *The Oxford Handbook of AI Governance*. Oxford University Press. ISBN: 9780197579329. https://doi.org/10.1093/oxfordhb/9780197579329.013.39. eprint: https://academic.oup.com/book/0/chapter/355438814/chapter-ag-pdf/54874439/book_41989_section_355438814.ag.pdf.

21. Brown, T., Mann, B., Ryder, N., Subbiah, M., Kaplan, J. D., Dhariwal, P., Neelakantan, A., Shyam, P., Sastry, G., Askell, A., et al. (2020). Language models are few-shot learners. Advances in neural information processing systems *33*, 1877–1901.

22. OpenAI (2023). *GPT-4 Technical Report*. Preprint at arXiv, https://doi.org/10.48550/arXiv.2303.08774.

23. Touvron, H., Lavril, T., Izacard, G., Martinet, X., Lachaux, M.-A., Lacroix, T., Rozière, B., Goyal, N., Hambro, E., Azhar, F., et al. (2023). *Llama: Open and Efficient Foundation Language Models*. Preprint at arXiv, https://doi.org/10.48550/arXiv.2302.13971.

24. Xiao, L. and Chen, X. (2023). *Enhancing LLM with Evolutionary Fine Tuning for News Summary Generation*. Preprint at arXiv, https://doi.org/10.48550/arXiv.2307.02839.

25. Jawahar, G., Abdul-Mageed, M., Lakshmanan, L., and Ding, D. (2024). "LLM Performance Predictors are good initializers for Architecture Search". *Findings of the Association for Computational Linguistics ACL 2024*. Ed. by L.-W. Ku, A. Martins, and V. Srikumar. Bangkok, Thailand and virtual meeting: Association for Computational Linguistics, 10540–10560.

26. Kumar, V., Gleyzer, L., Kahana, A., Shukla, K., and Karniadakis, G. E. (2023). MYCRUNCHGPT: A LLM ASSISTED FRAMEWORK FOR SCIENTIFIC MACHINE LEARNING. Journal of Machine Learning for Modeling and Computing *4*.

27. Varghese, J. and Chapiro, J. (2023). ChatGPT: The transformative influence of generative AI on science and healthcare. Journal of Hepatology.

28. Peters, M. E., Neumann, M., Iyyer, M., Gardner, M., Clark, C., Lee, K., and Zettlemoyer, L. (2018). "Deep Contextualized Word Representations". *Proceedings of the 2018 Conference of the North American Chapter of the Association for Computational Linguistics:*

Human Language Technologies, Volume 1 (Long Papers). Ed. by M. Walker, H. Ji, and A. Stent. New Orleans, Louisiana: Association for Computational Linguistics, 2227–2237.

29. Wolf, F. A., Angerer, P., and Theis, F. J. (2018). SCANPY: large-scale single-cell gene expression data analysis. Genome biology *19*, 1–5.

30. Chu, S.-K., Zhao, S., Shyr, Y., and Liu, Q. (2022). Comprehensive evaluation of noise reduction methods for single-cell RNA sequencing data. Briefings in bioinformatics *23*, bbab565.

31. Gao, Y., Myers, S., Chen, S., Dligach, D., Miller, T. A., Bitterman, D., Churpek, M., and Afshar, M. (2024). "When Raw Data Prevails: Are Large Language Model Embeddings Effective in Numerical Data Representation for Medical Machine Learning Applications?" *Findings of the Association for Computational Linguistics: EMNLP 2024*. Ed. by Y. Al-Onaizan, M. Bansal, and Y.-N. Chen. Miami, Florida, USA: Association for Computational Linguistics, 5414–5428. https://doi.org/10.18653/v1/2024.findings-emnlp.311.

32. Chen, T., Kornblith, S., Norouzi, M., and Hinton, G. (2020). "A simple framework for contrastive learning of visual representations". *International conference on machine learning*. PMLR, 1597–1607.

33. Musgrave, K., Belongie, S., and Lim, S.-N. (2020). *PyTorch Metric Learning*. Preprint at arXiv, https://doi.org/10.48550/arXiv.2008.09164.

34. Agarap, A. F. (2018). *Deep Learning Using Rectified Linear Units (ReLU)*. Preprint at arXiv, https://doi.org/10.48550/arXiv.1803.08375.

35. Harris, C. R., Millman, K. J., Van Der Walt, S. J., Gommers, R., Virtanen, P., Cournapeau, D., Wieser, E., Taylor, J., Berg, S., Smith, N. J., et al. (2020). Array programming with NumPy. Nature *585*, 357–362.

36. Ashburner, M., Ball, C. A., Blake, J. A., Botstein, D., Butler, H., Cherry, J. M., Davis, A. P., Dolinski, K., Dwight, S. S., Eppig, J. T., et al. (2000). Gene ontology: tool for the unification of biology. Nature genetics *25*, 25–29.

37. Aleksander, S. A., Balhoff, J., Carbon, S., Cherry, J. M., Drabkin, H. J., Ebert, D., Feuermann, M., Gaudet, P., Harris, N. L., et al. (2023). The gene ontology knowledgebase in 2023. Genetics *224*, iyad031.

38. Fang, Z., Liu, X., and Peltz, G. (2023). GSEApy: a comprehensive package for performing gene set enrichment analysis in Python. Bioinformatics *39*, btac757.

39. Krämer, A., Green, J., Pollard Jr, J., and Tugendreich, S. (2014). Causal analysis approaches in ingenuity pathway analysis. Bioinformatics *30*, 523–530.

40. Gayoso, A., Steier, Z., Lopez, R., Regier, J., Nazor, K. L., Streets, A., and Yosef, N. (2021). Joint probabilistic modeling of single-cell multi-omic data with totalVI. Nature methods *18*, 272–282.

41. Zhu, B., Chen, S., Bai, Y., Chen, H., Liao, G., Mukherjee, N., Vazquez, G., McIlwain, D. R., Tzankov, A., Lee, I. T., et al. (2023). Robust single-cell matching and multimodal analysis using shared and distinct features. Nature Methods *20*, 304–315.

42. Papineni, K., Roukos, S., Ward, T., and Zhu, W.-J. (2002). “Bleu: a method for automatic evaluation of machine translation”. *Proceedings of the 40th annual meeting of the Association for Computational Linguistics*, 311–318.

43. Freitag, M., Foster, G., Grangier, D., Ratnakar, V., Tan, Q., and Macherey, W. (2021). Experts, errors, and context: A large-scale study of human evaluation for machine translation. Transactions of the Association for Computational Linguistics *9*, 1460–1474.

44. Luecken, M. D., Büttner, M., Chaichoompu, K., Danese, A., Interlandi, M., Müller, M. F., Strobl, D. C., Zappia, L., Dugas, M., Colomé-Tatché, M., et al. (2022). Benchmarking atlas-level data integration in single-cell genomics. Nature methods *19*, 41–50.

45. Pedregosa, F., Varoquaux, G., Gramfort, A., Michel, V., Thirion, B., Grisel, O., Blondel, M., Prettenhofer, P., Weiss, R., Dubourg, V., et al. (2011). Scikit-learn: Machine learning in Python. the Journal of machine Learning research *12*, 2825–2830.

46. Virtanen, P., Gommers, R., Oliphant, T. E., Haberland, M., Reddy, T., Cournapeau, D., Burovski, E., Peterson, P., Weckesser, W., Bright, J., et al. (2020). SciPy 1.0: fundamental algorithms for scientific computing in Python. Nature methods *17*, 261–272.

47. Suzgun, M. and Kalai, A. T. (2024). *Meta-Prompting: Enhancing Language Models with Task-Agnostic Scaffolding*. Preprint at arXiv, https://doi.org/10.48550/arXiv.2401.12954.

48. Kiselev, V. Y., Kirschner, K., Schaub, M. T., Andrews, T., Yiu, A., Chandra, T., Natarajan, K. N., Reik, W., Barahona, M., Green, A. R., et al. (2017). SC3: consensus clustering of single-cell RNA-seq data. Nature methods *14*, 483–486.

49. Lopez, R., Regier, J., Cole, M. B., Jordan, M. I., and Yosef, N. (2018). Deep generative modeling for single-cell transcriptomics. Nature methods *15*, 1053–1058.

50. Korsunsky, I., Millard, N., Fan, J., Slowikowski, K., Zhang, F., Wei, K., Baglaenko, Y., Brenner, M., Loh, P.-r., and Raychaudhuri, S. (2019). Fast, sensitive and accurate integration of single-cell data with Harmony. Nature methods *16*, 1289–1296.

51. Haghverdi, L., Lun, A. T., Morgan, M. D., and Marioni, J. C. (2018). Batch effects in single-cell RNA-sequencing data are corrected by matching mutual nearest neighbors. Nature biotechnology *36*, 421–427.

52. Radford, A., Wu, J., Child, R., Luan, D., Amodei, D., Sutskever, I., et al. (2019). Language models are unsupervised multitask learners. OpenAI blog *1*, 9.

53. Hou, W. and Ji, Z. (2024). Assessing GPT-4 for cell type annotation in single-cell RNA-seq analysis. Nature Methods, 1–4.

54. Devlin, J., Chang, M.-W., Lee, K., and Toutanova, K. (2019). “BERT: Pre-training of Deep Bidirectional Transformers for Language Understanding”. *Proceedings of the 2019 Conference of the North American Chapter of the Association for Computational Linguistics:*

Human Language Technologies, Volume 1 (Long and Short Papers). Ed. by J. Burstein, C. Doran, and T. Solorio. Minneapolis, Minnesota: Association for Computational Linguistics, 4171–4186.

55. Dong, M., Wang, B., Wei, J., O. Fonseca, A. H. de, Perry, C. J., Frey, A., Ouerghi, F., Foxman, E. F., Ishizuka, J. J., Dhodapkar, R. M., et al. (2023). Causal identification of single-cell experimental perturbation effects with CINEMA-OT. Nature Methods, 1–11.

56. Lotfollahi, M., Klimovskaia Susmelj, A., De Donno, C., Hetzel, L., Ji, Y., Ibarra, I. L., Srivatsan, S. R., Naghipourfar, M., Daza, R. M., Martin, B., et al. (2023). Predicting cellular responses to complex perturbations in high-throughput screens. Molecular Systems Biology, e11517.

57. Roohani, Y., Huang, K., and Leskovec, J. (2023). Predicting transcriptional outcomes of novel multigene perturbations with gears. Nature Biotechnology, 1–9.

58. Landa, B., Zhang, T. T., and Kluger, Y. (2022). Biwhitening reveals the rank of a count matrix. SIAM Journal on Mathematics of Data Science *4*, 1420–1446.

59. Cuturi, M. (2013). Sinkhorn distances: Lightspeed computation of optimal transport. Advances in neural information processing systems *26*.

60. Petukhova, A., Matos-Carvalho, J. P., and Fachada, N. (2025). Text clustering with large language model embeddings. International Journal of Cognitive Computing in Engineering *6*, 100–108.

61. Keraghel, I., Morbieu, S., and Nadif, M. (2024). “Beyond words: a comparative analysis of LLM embeddings for effective clustering”. *International Symposium on Intelligent Data Analysis*. Springer, 205–216.

62. Asudani, D. S., Nagwani, N. K., and Singh, P. (2023). Impact of word embedding models on text analytics in deep learning environment: a review. Artificial intelligence review *56*, 10345–10425.

63. Cao, Z.-J. and Gao, G. (2022). Multi-omics single-cell data integration and regulatory inference with graph-linked embedding. Nature Biotechnology *40*, 1458–1466.

64. Huang, K., Lopez, R., Hütter, J.-C., Kudo, T., Rios, A., and Regev, A. (2024). “Sequential Optimal Experimental Design of Perturbation Screens Guided by Multi-modal Priors”. *International Conference on Research in Computational Molecular Biology*. Springer, 17–37.

65. Zhang, Y., Li, Y., Cui, L., Cai, D., Liu, L., Fu, T., Huang, X., Zhao, E., Zhang, Y., Chen, Y., et al. (2023). *Siren’s Song in the AI Ocean: A Survey on Hallucination in Large Language Models*. Preprint at arXiv, https://doi.org/10.48550/arXiv.2309.01219.

66. Jiang, A. Q., Sablayrolles, A., Mensch, A., Bamford, C., Chaplot, D. S., Casas, D. de las, Bressand, F., Lengyel, G., Lample, G., Saulnier, L., et al. (2023). *Mistral 7B*. Preprint at arXiv, https://doi.org/10.48550/arXiv.2310.06825.

67. Luo, R., Sun, L., Xia, Y., Qin, T., Zhang, S., Poon, H., and Liu, T.-Y. (2022). BioGPT: generative pre-trained transformer for biomedical text generation and mining. Briefings in Bioinformatics *23*, bbac409.

68. Anthropic, A. (2023). Model card and evaluations for claude models. Anthropic Blog.

69. Anil, R., Dai, A. M., Firat, O., Johnson, M., Lepikhin, D., Passos, A., Shakeri, S., Taropa, E., Bailey, P., Chen, Z., et al. (2023). *PaLM 2 Technical Report*. Preprint at arXiv, https://doi.org/10.48550/arXiv.2305.10403.

70. Hao, Y., Hao, S., Andersen-Nissen, E., Mauck, W. M., Zheng, S., Butler, A., Lee, M. J., Wilk, A. J., Darby, C., Zager, M., et al. (2021). Integrated analysis of multimodal single-cell data. Cell *184*, 3573–3587.

71. Safran, M., Dalah, I., Alexander, J., Rosen, N., Iny Stein, T., Shmoish, M., Nativ, N., Bahir, I., Doniger, T., Krug, H., et al. (2010). GeneCards Version 3: the human gene integrator. Database *2010*.

72. Wei, J., Wang, X., Schuurmans, D., Bosma, M., Xia, F., Chi, E., Le, Q. V., Zhou, D., et al. (2022). Chain-of-thought prompting elicits reasoning in large language models. Advances in neural information processing systems *35*, 24824–24837.

73. Martin, F. J., Amode, M. R., Aneja, A., Austine-Orimoloye, O., Azov, A. G., Barnes, I., Becker, A., Bennett, R., Berry, A., Bhai, J., et al. (2023). Ensembl 2023. Nucleic acids research *51*, D933–D941.

74. Soneson, C. and Robinson, M. D. (2018). Bias, robustness and scalability in single-cell differential expression analysis. Nature methods *15*, 255–261.

75. Armingol, E., Officer, A., Harismendy, O., and Lewis, N. E. (2021). Deciphering cell–cell interactions and communication from gene expression. Nature Reviews Genetics *22*, 71–88.

76. Stephenson, E., Reynolds, G., Botting, R. A., Calero-Nieto, F. J., Morgan, M. D., Tuong, Z. K., Bach, K., Sungnak, W., Worlock, K. B., Yoshida, M., et al. (2021). Single-cell multi-omics analysis of the immune response in COVID-19. Nature medicine *27*, 904–916.

77. Sikkema, L., Ramírez-Suástegui, C., Strobl, D. C., Gillett, T. E., Zappia, L., Madissoon, E., Markov, N. S., Zaragosi, L.-E., Ji, Y., Ansari, M., et al. (2023). An integrated cell atlas of the lung in health and disease. Nature Medicine, 1–15.

78. Litviňuková, M., Talavera-López, C., Maatz, H., Reichart, D., Worth, C. L., Lindberg, E. L., Kanda, M., Polanski, K., Heinig, M., Lee, M., et al. (2020). Cells of the adult human heart. Nature *588*, 466–472.

79. Miao, Z., Humphreys, B. D., McMahon, A. P., and Kim, J. (2021). Multi-omics integration in the age of million single-cell data. Nature Reviews Nephrology *17*, 710–724.

80. Zeng, H. (2022). What is a cell type and how to define it? Cell *185*, 2739–2755.

81. Li, Y. Y., An, J., and Jones, S. J. (2011). A computational approach to finding novel targets for existing drugs. PLoS computational biology *7*, e1002139.

82. Kumar, R. and Saha, P. (2022). A review on artificial intelligence and machine learning to improve cancer management and drug discovery. International Journal for Research in Applied Sciences and Biotechnology *9*, 149–156.

83. Abdelazim, M. A., Nasr, M. M., and Ead, W. M. (2020). A survey on classification analysis for cancer genomics: Limitations and novel opportunity in the era of cancer classification and Target Therapies. Annals of Tropical Medicine and Public Health *23*, 24.

84. Spudich, J. A. (2014). Hypertrophic and dilated cardiomyopathy: four decades of basic research on muscle lead to potential therapeutic approaches to these devastating genetic diseases. Biophysical journal *106*, 1236–1249.

85. Chaffin, M., Papangeli, I., Simonson, B., Akkad, A.-D., Hill, M. C., Arduini, A., Fleming, S. J., Melanson, M., Hayat, S., Kost-Alimova, M., et al. (2022). Single-nucleus profiling of human dilated and hypertrophic cardiomyopathy. Nature *608*, 174–180.

86. Pagiatakis, C. and Di Mauro, V. (2021). The emerging role of epigenetics in therapeutic targeting of cardiomyopathies. International Journal of Molecular Sciences *22*, 8721.

87. Fang, C., Lv, Z., Yu, Z., Wang, K., Xu, C., Li, Y., and Wang, Y. (2022). Exploration of dilated cardiomyopathy for biomarkers and immune microenvironment: Evidence from RNA-seq. BMC Cardiovascular Disorders *22*, 320.

88. Feng, Y., Cai, L., Hong, W., Zhang, C., Tan, N., Wang, M., Wang, C., Liu, F., Wang, X., Ma, J., et al. (2022). Rewiring of 3D chromatin topology orchestrates transcriptional reprogramming and the development of human dilated cardiomyopathy. Circulation *145*, 1663–1683.

89. Balashanmugam, M. V., Shivanandappa, T. B., Nagarethinam, S., Vastrad, B., and Vastrad, C. (2019). Analysis of differentially expressed genes in coronary artery disease by integrated microarray analysis. Biomolecules *10*, 35.

90. Barrangou, R. and Doudna, J. A. (2016). Applications of CRISPR technologies in research and beyond. Nature biotechnology *34*, 933–941.

91. Davies, R. R., Kaple, R. K., Mandapati, D., Gallo, A., Botta Jr, D. M., Elefteriades, J. A., and Coady, M. A. (2007). Natural history of ascending aortic aneurysms in the setting of an unreplaced bicuspid aortic valve. The Annals of thoracic surgery *83*, 1338–1344.

92. Moreno-Loshuertos, R., Movilla, N., Marco-Brualla, J., Soler-Agesta, R., Ferreira, P., Enríquez, J. A., and Fernández-Silva, P. (2023). A Mutation in Mouse MT-ATP6 Gene Induces Respiration Defects and Opposed Effects on the Cell Tumorigenic Phenotype. International Journal of Molecular Sciences *24*, 1300.

93. Stendel, C., Neuhofer, C., Floride, E., Yuqing, S., Ganetzky, R. D., Park, J., Freisinger, P., Kornblum, C., Kleinle, S., Schöls, L., et al. (2020). Delineating MT-ATP6-associated disease: From isolated neuropathy to early onset neurodegeneration. Neurology Genetics *6*.

94. Lotfollahi, M., Wolf, F. A., and Theis, F. J. (2019). scGen predicts single-cell perturbation responses. Nature methods *16*, 715–721.

95. Dixit, A., Parnas, O., Li, B., Chen, J., Fulco, C. P., Jerby-Arnon, L., Marjanovic, N. D., Dionne, D., Burks, T., Raychowdhury, R., et al. (2016). Perturb-Seq: dissecting molecular circuits with scalable single-cell RNA profiling of pooled genetic screens. cell *167*, 1853–1866.

96. Szałata, A., Benz, A., Cannoodt, R., Cortes, M., Fong, J., Kuppasani, S., Lieberman, R., Liu, T., Mas-Rosario, J. A., Meinl, R., et al. (2024). A benchmark for prediction of transcriptomic responses to chemical perturbations across cell types. Advances in Neural Information Processing Systems *37*, 20566–20616.

97. Kipf, T. N. and Welling, M. (2017). "Semi-Supervised Classification with Graph Convolutional Networks". *International Conference on Learning Representations*.

98. Replogle, J. M., Saunders, R. A., Pogson, A. N., Hussmann, J. A., Lenail, A., Guna, A., Mascibroda, L., Wagner, E. J., Adelman, K., Lithwick-Yanai, G., et al. (2022). Mapping information-rich genotype-phenotype landscapes with genome-scale Perturb-seq. Cell *185*, 2559–2575.

99. Norman, T. M., Horlbeck, M. A., Replogle, J. M., Ge, A. Y., Xu, A., Jost, M., Gilbert, L. A., and Weissman, J. S. (2019). Exploring genetic interaction manifolds constructed from rich single-cell phenotypes. Science *365*, 786–793.

100. Adamson, B., Norman, T. M., Jost, M., Cho, M. Y., Nuñez, J. K., Chen, Y., Villalta, J. E., Gilbert, L. A., Horlbeck, M. A., Hein, M. Y., et al. (2016). A multiplexed single-cell CRISPR screening platform enables systematic dissection of the unfolded protein response. Cell *167*, 1867–1882.

101. Wenteler, A., Occhetta, M., Branson, N., Curean, V., Huebner, M., Dee, W., Connell, W., Chung, S. P., Hawkins-Hooker, A., Ektefaie, Y., et al. (2025). "PertEval-scFM: Benchmarking Single-Cell Foundation Models for Perturbation Effect Prediction". *Forty-second International Conference on Machine Learning*.

102. Visscher, P. M., Brown, M. A., McCarthy, M. I., and Yang, J. (2012). Five years of GWAS discovery. The American Journal of Human Genetics *90*, 7–24.

103. Mimitou, E. P., Lareau, C. A., Chen, K. Y., Zorzetto-Fernandes, A. L., Hao, Y., Takeshima, Y., Luo, W., Huang, T.-S., Yeung, B. Z., Papalexi, E., et al. (2021). Scalable, multimodal profiling of chromatin accessibility, gene expression and protein levels in single cells. Nature biotechnology *39*, 1246–1258.

104. Liu, T. (2025a). scELMo. Zendo. https://doi.org/10.5281/zenodo.17298922.

105. — (2025b). *scELMo embedding library. Zendo*. https://doi.org/10.5281/zenodo.17517204.

106. Chen, J., Xu, H., Tao, W., Chen, Z., Zhao, Y., and Han, J.-D. J. (2023). Transformer for one stop interpretable cell type annotation. Nature Communications *14*, 223.

107. Wang, Y., Liu, T., and Zhao, H. (2022). ResPAN: a powerful batch correction model for scRNA-seq data through residual adversarial networks. Bioinformatics *38*, 3942–3949.

108. Pullin, J. M. and McCarthy, D. J. (2024). A comparison of marker gene selection methods for single-cell RNA sequencing data. Genome Biology *25*, 56.

109. Granja, J. M., Klemm, S., McGinnis, L. M., Kathiria, A. S., Mezger, A., Corces, M. R., Parks, B., Gars, E., Liedtke, M., Zheng, G. X., et al. (2019). Single-cell multiomic analysis identifies regulatory programs in mixed-phenotype acute leukemia. Nature biotechnology *37*, 1458–1465.

110. Wilk, A. J., Lee, M. J., Wei, B., Parks, B., Pi, R., Martínez-Colón, G. J., Ranganath, T., Zhao, N. Q., Taylor, S., Becker, W., et al. (2021). Multi-omic profiling reveals widespread dysregulation of innate immunity and hematopoiesis in COVID-19. Journal of Experimental Medicine *218*, e20210582.

111. Green, T. D., Peidli, S., Shen, C., Gross, T., Min, J., Garda, S., Taylor-King, J. P., Marks, D. S., Luna, A., Blüthgen, N., et al. (2022). "scPerturb: Information Resource for Harmonized Single-Cell Perturbation Data". *NeurIPS 2022 Workshop on Learning Meaningful Representations of Life*.

112. Program, C. C. S., Abdulla, S., Aevermann, B., Assis, P., Badajoz, S., Bell, S. M., Bezzi, E., Cakir, B., Chaffer, J., Chambers, S., et al. (2025). CZ CELLxGENE Discover: a single-cell data platform for scalable exploration, analysis and modeling of aggregated data. Nucleic acids research *53*, D886–D900.

113. McInnes, L., Healy, J., Saul, N., and Großberger, L. (2018). UMAP: Uniform Manifold Approximation and Projection. Journal of Open Source Software *3*, 861. https://doi.org/10.21105/joss.00861.

114. Chang, M. T., Shanahan, F., Nguyen, T. T. T., Staben, S. T., Gazzard, L., Yamazoe, S., Wertz, I. E., Piskol, R., Yang, Y. A., Modrusan, Z., et al. (2022). Identifying transcriptional programs underlying cancer drug response with TraCe-seq. Nature Biotechnology *40*, 86–93.

115. Baron, M., Veres, A., Wolock, S. L., Faust, A. L., Gaujoux, R., Vetere, A., Ryu, J. H., Wagner, B. K., Shen-Orr, S. S., Klein, A. M., et al. (2016). A single-cell transcriptomic map of the human and mouse pancreas reveals inter-and intra-cell population structure. Cell systems *3*, 346–360.

116. Muraro, M. J., Dharmadhikari, G., Grün, D., Groen, N., Dielen, T., Jansen, E., Van Gurp, L., Engelse, M. A., Carlotti, F., De Koning, E. J., et al. (2016). A single-cell transcriptome atlas of the human pancreas. Cell systems *3*, 385–394.

117. Xin, Y., Kim, J., Okamoto, H., Ni, M., Wei, Y., Adler, C., Murphy, A. J., Yancopoulos, G. D., Lin, C., and Gromada, J. (2016). RNA sequencing of single human islet cells reveals type 2 diabetes genes. Cell metabolism *24*, 608–615.

118. Lawlor, N., George, J., Bolisetty, M., Kursawe, R., Sun, L., Sivakamasundari, V., Kycia, I., Robson, P., and Stitzel, M. L. (2017). Single-cell transcriptomes identify human islet cell

signatures and reveal cell-type–specific expression changes in type 2 diabetes. Genome research *27*, 208–222.

119. Li, Y., Ren, P., Dawson, A., Vasquez, H. G., Ageedi, W., Zhang, C., Luo, W., Chen, R., Li, Y., Kim, S., et al. (2020). Single-cell transcriptome analysis reveals dynamic cell populations and differential gene expression patterns in control and aneurysmal human aortic tissue. Circulation *142*, 1374–1388.

120. Han, X., Wang, R., Zhou, Y., Fei, L., Sun, H., Lai, S., Saadatpour, A., Zhou, Z., Chen, H., Ye, F., et al. (2018). Mapping the mouse cell atlas by microwell-seq. Cell *172*, 1091–1107.

121. Stickels, R. R., Murray, E., Kumar, P., Li, J., Marshall, J. L., Di Bella, D. J., Arlotta, P., Macosko, E. Z., and Chen, F. (2021). Highly sensitive spatial transcriptomics at near-cellular resolution with Slide-seqV2. Nature biotechnology *39*, 313–319.

122. Cordero, P., Parikh, V. N., Chin, E. T., Erbilgin, A., Gloudemans, M. J., Shang, C., Huang, Y., Chang, A. C., Smith, K. S., Dewey, F., et al. (2019). Pathologic gene network rewiring implicates PPP1R3A as a central regulator in pressure overload heart failure. Nature communications *10*, 2760.
